# Conserved chromosomal clustering of genes governed by chromatin regulators in *Drosophila*

**DOI:** 10.1186/gb-2008-9-9-r134

**Published:** 2008-09-10

**Authors:** Enrique Blanco, Miguel Pignatelli, Sergi Beltran, Adrià Punset, Silvia Pérez-Lluch, Florenci Serras, Roderic Guigó, Montserrat Corominas

**Affiliations:** 1 Departament de Genètica and Institut de Biomedicina de la Universitat de Barcelona (IBUB), Universitat de Barcelona, Diagonal 645, 08028 Barcelona, Catalonia, Spain; 2 Centre de Regulació Genòmica, Parc de Recerca Biomèdica de Barcelona, Dr. Aiguader 88, 08003 Barcelona, Catalonia, Spain; 3 Grup de Recerca en Informàtica Biomèdica, Institut Municipal d'Investigació Mèdica - Universitat Pompeu Fabra Barcelona, Catalonia, Spain; 4 Current address: Instituto Cavanilles of Biodiversity and Evolutionary Biology, University of Valencia, Apdo 22085, 46071 Valencia, Spain and CIBER of Epidemiology and Public Health (CIBERESP)

## Abstract

**Background:**

The trithorax group (trxG) and Polycomb group (PcG) proteins are responsible for the maintenance of stable transcriptional patterns of many developmental regulators. They bind to specific regions of DNA and direct the post-translational modifications of histones, playing a role in the dynamics of chromatin structure.

**Results:**

We have performed genome-wide expression studies of *trx *and *ash2 *mutants in *Drosophila melanogaster*. Using computational analysis of our microarray data, we have identified 25 clusters of genes potentially regulated by TRX. Most of these clusters consist of genes that encode structural proteins involved in cuticle formation. This organization appears to be a distinctive feature of the regulatory networks of TRX and other chromatin regulators, since we have observed the same arrangement in clusters after experiments performed with ASH2, as well as in experiments performed by others with NURF, dMyc, and ASH1. We have also found many of these clusters to be significantly conserved in *D. simulans*, *D. yakuba*, *D. pseudoobscura *and partially in *Anopheles gambiae*.

**Conclusion:**

The analysis of genes governed by chromatin regulators has led to the identification of clusters of functionally related genes conserved in other insect species, suggesting this chromosomal organization is biologically important. Moreover, our results indicate that TRX and other chromatin regulators may act globally on chromatin domains that contain transcriptionally co-regulated genes.

## Abstract 2

Transcriptional analysis of chromatin regulator mutants in *Drosophila melanogaster* identified clusters of functionally related genes conserved in other insect species.

## Background

Differential gene expression is essential to the cellular diversity required for adequate pattern formation and organogenesis during the first stages of development in multicellular organisms. Thereafter, epigenetic regulatory systems must ensure the maintenance of these gene expression patterns to preserve cell identity in adulthood [1]. Regulation of transcription is, therefore, crucial to proper temporal and spatial gene expression throughout development. The complex transcriptional regulatory code that governs the different gene expression programs of an organism involves many different actors, such as transcription factors, regulatory sequences in the genome, chromatin structure and modification states [2]. Chromatin packaging plays a central role during gene transcription by controlling the access of the RNA polymerase II transcriptional machinery and other gene regulatory elements (such as transcription factors) to the promoter region of the genes [3,4]. The dynamics of chromatin structure is controlled through multiple mechanisms, such as nucleosome positioning, chromatin remodeling and histone post-translational modifications [5].

Gene regulation can occur in the genome at distinct levels of organization: individual genes, chromosomal domains and entire chromosomes [6]. Thus, a set of transcriptionally active genes and the regulatory elements necessary for their correct expression are generally associated with open chromatin domains, while silent genes are embedded in more compact chromatin regions [7]. The main effect of such domains on genome organization is observed in the non-random distribution of genes in a genome, which can favor coordinated gene expression. In fact, the interplay of genome rearrangements, gene expression mechanisms and evolutionary forces could explain the complex landscape of gene regulation [8].

Since the publication of the sequence of many eukaryotic genomes [9-12], several whole-genome studies about genome organization have established the existence of clusters of co-expressed genes, in some cases functionally related (see [8] for a comprehensive review). Examples have been found in many species such as yeast [13,14], worm [15,16] or human [17,18]. In *D. melanogaster*, the presence of clusters has been studied by several groups. Ueda *et al*. [19] found that genes controlling circadian rhythms tend to be grouped in local clusters on chromosomes, suggesting this is due to higher order chromatin structures. Spellman and Rubin [20] analyzed the chromosomal position of gene expression profiles from 88 different experimental conditions and found that over 20% of all genes were clustered into co-regulated groups of 10-30 genes of unrelated function. Boutanaev *et al*. [21] identified 1,661 testes-specific genes, one-third of which were clustered on chromosomes in groups of three or more genes. The effect of chromatin structure on a particular cluster of five genes in the previous screening [21] was successfully validated by Kalmykova *et al*. [22]. Belyakin *et al*. [23] reported 1,036 genes that are arranged in clusters located in 52 underreplication regions of the larval salivary gland polytene chromosomes.

Epigenetic regulation of gene expression is necessary for the correct deployment of developmental programs and for the maintenance of cell fates. The Polycomb and Trithorax epigenetic system, initially discovered in *D. melanogaster*, is responsible for the maintenance of gene expression throughout late development and adulthood. Polycomb group (PcG) proteins are required to prevent inappropriate expression of homeotic genes, while trithorax group (trxG) proteins seem to work antagonistically as anti-repressors. Recent studies have identified and characterized several multiprotein complexes containing these transcriptional regulators. They control transcription through multistep mechanisms that involve histone modification, chromatin remodeling, and interaction with general transcription factors. In flies, PcG and trxG complexes are recruited to certain regulatory sequence response elements of the genome denominated PRE/TREs (see [24-27] for a review on trxG and PcG proteins).

Systematic examination of gene expression patterns using microarrays can provide a global picture of the distinct regulatory networks of different genomes [28-31]. In particular, several genome-wide expression experiments involving members of trxG have recently been published [32-34]. Trithorax (*trx*), the first isolated member of the trxG, is required throughout embryonic and larval development for the correct differentiation in the adult [35]. The *trx *gene encodes a histone methyltransferase that can modify lysine 4 of histone 3 (H3K4). This methylation is an epigenetic mark associated with transcriptionally active genes [36]. In the work presented here we have combined the expression profiles obtained from microarray experiments with exhaustive bioinformatic analyses that include gene clustering, comparative genomics and functional annotation to gain insight into the role of trxG proteins. Our results show the existence of evolutionarily conserved chromosomal clusters with most of the genes being also regulated by other chromatin regulators, and functionally annotated as components of the cuticle.

## Results

### Whole-genome expression analysis of *trx *mutants

In order to investigate the molecular signature of the *trx *mutants in *Drosophila melanogaster*, we have compared whole-genome expression profiles of *trx *mutant third instar larvae and wild-type larvae (see Materials and methods). We designed two-color cDNA microarrays containing 12,120 genes annotated in RefSeq from *D. melanogaster *[37]. The analysis of the microarray experiments identified 535 genes showing a statistically significant change (at least 2-fold change, *p*-value <0.05) in expression between mutant and wild-type samples (see Materials and methods). Of these, 260 were over-expressed and 275 were under-expressed in mutant larvae (Additional data file 1).

We mapped these deregulated genes to the fly genome (assembly dm2, April 2004) using the RefSeq [37] track of the UCSC genome browser [38], and the chromosomal distribution is shown in Table 1. The number of RefSeq genes annotated on each chromosome is also displayed. We mapped more co-expressed genes on chromosome 3L than on any other chromosome (30% of 535 deregulated genes; Table 1): 69 up-regulated genes (*p*-value <10^-2^) and 94 down-regulated genes (*p*-value <10^-8^). Chromosomes 2R and 3R are, however, richer in number of annotated genes (3,993 and 4,843 genes respectively, compared to 3,775 genes in chromosome 3L in Table 1).

**Table 1:** Genome distribution of genes and clusters deregulated in *trx *mutants

Chromosome	Length	Genes	TRX ↑	TRX ↓	TRX ↑+↓	Clusters ↑	Clusters ↓	Clusters ↑+↓
2L	22,855,998	3,594	39	26	65	0	1	1
2R	21,182,128	3,993	54	51	105	2	2	4
3L	24,247,342	3,775	69	94	163	6	9	15
3R	28,463,162	4,843	59	71	130	2	2	4
X	22,668,884	3,238	39	32	71	0	1	1
4	1,307,279	227	0	1	1	0	0	0
								
Total	120,724,793	19,670	260	275	535	10	15	25

The following information is displayed for each chromosome from *D. melanogaster*: length, number of genes, number of up-regulated genes, number of down-regulated genes, total number of deregulated genes in the microarray, number of up-regulated clusters, number of down-regulated clusters, and total number of clusters.

### Chromosomal clustering of genes deregulated in *trx *mutants

Since chromatin modifications are typically associated with the coordinated expression of groups of nearby genes [3] and the analysis of different transcriptome datasets has shown that genes with a similar expression pattern are frequently located next to one another in the linear genome [21,39], our next step was to determine whether deregulated genes in our *trx *mutants are located in close proximity in the fly genome (chromosomal clusters). There are many possible definitions of what a cluster of genes is (see [8] for a review). Here, we define a cluster as a group of genes located close to each other on the same chromosome in the genome, but not necessarily adjacent, that showed the same expression pattern (up-regulation or down-regulation) in the microarray experiment (see Materials and methods).

**Figure 1:**
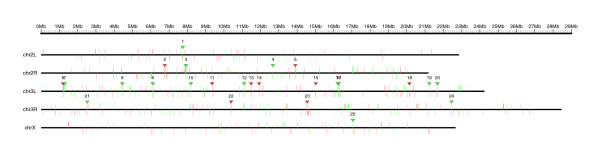
Genomic map of clusters of genes deregulated in *trx *mutants. The location of each gene significantly deregulated in the microarray is indicated with a vertical line (up-regulated genes in red, down-regulated genes in green). Genes in the forward strand are displayed above the chromosome line; genes in the reverse strand are displayed below. Clusters of genes are indicated with a triangle in red or green according to their expression. The genome map was produced using the program GFF2PS [102].

Chromosomal clusters can be identified computationally [20,40]. We detected 97 genes, organized in 25 genomic clusters, that are deregulated in *trx *deficient larvae (10 clusters of up-regulated genes and 15 clusters of down-regulated genes; Table 1), using the program REEF [41] with the following parameters: window length, 25,000 bp; window step, 1,000 bp; minimal number of co-expressed genes, 3; q-value ≤0.05. The chromosomal distribution of clusters and genes along the genome of *D. melanogaster *is shown in Figure 1 (up-regulated genes are depicted in red, down-regulated genes in green; the genomic position of each cluster is represented with the corresponding red or green triangle and each cluster is labeled with the same identifier used in Table 2). Clusters of genes deregulated in *trx *mutant larvae are not uniformly distributed along the genome: 15 out of 25 clusters (60%) are located on chromosome 3L (Table 1). Remarkably, the proportion of genes in clusters increases dramatically in chromosome 3L: 62 genes out of 163 deregulated genes mapped to this chromosome are clustered (38%), as opposed to only 35 genes out of 372 deregulated genes mapped to the other chromosomes (9%) (Additional data file 2).

**Table 2:** Clusters of genes deregulated in *trx *mutants

ID	Chromosome	Start	End	Regulation	Deregulated genes	No deregulated genes
1	2L	7,740,552	7,753,160	↓	*Acp1*, CG7214, CG7203	CG7211
2	2R	6,757,647	6,771,890	↑	CG9080, CG30029, CG7738, CG13224	CG13226, *Or47a*, CG9079
3	2R	7,906,941	7,941,371	↓	CG8836, CG8505, CG8510, CG8511, CG8520	*Or49a*, CG30048, CG30050, CG33626, CG33627, CG8515, CG13157, CG8834
4	2R	12,673,194	12,681,364	↓	CG30458, CG30457, CG10953	
5	2R	13,899,427	13,905,472	↑	CG18107, CG16836, CG15068	CG15067, *IM2*, *IM3*, CG15065
6	3L	1,190,902	1,211,770	↑	*LysB*, *LysC*, *LysD*, *LysP*, *LysS*	*LysE*
7	3L	1,286,480	1,296,909	↓	CG9149, CG2469, CG9186	CG2277
8	3L	4,429,174	4,447,704	↓	CG12607, CG11345, CG32241	CG15022, CG15023, CG15024
9	3L	6,097,832	6,125,586	↓	l(3)mbn, CG18779, CG18778, *Lcp65Ag2*, *Lcp65Ae*, CG32405, C32404, *Lcp65Ac*, *Lcp65Ab2*, *Lcp65Aa*	*Lcp65Ag1*, *Lcp65Af*, *Lcp65Ad*, *Lcp65Ab1*, CG18777
10	3L	8,189,385	8,196,898	↓	CG8012, CG13674, CG13678	
11	3L	9,350,364	9,359,229	↑	*Hsp26*, *Hsp23*, *Hsp27*	*Hsp67Ba*
12	3L	11,103,295	11,109,286	↓	CG7628, CG32074, CG14143	*nol*
13	3L	11,482,913	11,487,349	↑	*Sgs8*, *Sgs7*, *Sgs3*	CG33272
14	3L	11,917,595	11,926,172	↑	CG5883, CG7252, CG17826	
15	3L	15,017,322	15,026,980	↑	CG13461, CG18649, CG13460	CG13463
16	3L	16,230,688	16,260,799	↓	CG13069, CG13068, CG13067, CG13047, CG4962	CG4950, CG13066, CG13065, CG13050, CG13064, CG13049, CG13048, CG13046, CG13045
17	3L	16,266,728	16,289,521	↓	CG4982, CG13063, CG13042, CG13041, CG13060, CG13059	CG13044, CG13043, CG32160, CG13062, *Nplp3*
18	3L	20,138,463	20,154,238	↑	CG7290, CG7017, CG6933	CG6996, CG32224
19	3L	21,226,060	21,235,012	↓	CG11310, *Edg78E*, CG7658	CG7663
20	3L	21,664,564	21,691,822	↓	CG14569, CG14568, CG14566, CG14572, CG14565, CG14564	CG14573, CG14567, *Syn1*
21	3R	2,512,449	2,530,625	↓	*Ccp84Ag*, *Ccp84Ad*, *Ccp84Ab*, *Ccp84Aa*	*Ccp84Af*, *Ccp84Ae*, *Ccp84Ac*
22	3R	10,386,703	10,392,190	↑	CG14850, CG8087, CG14852	CG14851
23	3R	14,551,120	14,558,756	↑	CG7714, CG7715, CG14302	
24	3R	22,444,844	22,458,678	↓	CG5468, CG14240, CG6452, CG5476	CG6478, CG6447, CG6460, CG5471
25	X	17,040,356	17,065,159	↓	CG32564, CG10598, CG10597	CG32563, CG12995, CG18258, CG5162, CG12998, CG5172, CG12997

For each cluster we display: identifier, chromosome, genomic coordinates (start and end), regulation (up or down), co-expressed genes, and no co-expressed genes in the microarray.

The clusters reported here contain a total of 162 genes (97 deregulated genes and 65 genes whose change in expression was not significant), comprising in total 372,967 nucleotides, with an average gene density of 4.3 genes per 10 Kb. In contrast, the average gene density in the fruit fly genome is 1.6 genes per 10 Kb. The average length of the genes in clusters is 946 bp, while the length of the deregulated genes that are not clustered is 3,416 bp (the overall average for *D. melanogaster *is 6,976 bp). Since the REEF program approach is based on genomic proximity measured in number of nucleotides, this could favor artifactual cluster definition in gene-rich regions of the genome. To rule out this possibility, we have designed an alternative clustering algorithm based on measuring the number of co-expressed genes within a window containing a fixed number of annotated genes, rather than a fixed number of nucleotides (see Materials and methods for further details). Results obtained with our clustering strategy are highly concordant with those produced by the REEF program (Additional data file 3): 27 clusters were detected (22 identical clusters, 2 clusters with additional genes, 3 new clusters and 1 missing cluster). Therefore, the high gene density observed in our clusters is not the consequence of any computational limitation in the clustering method. Given the high concordance of the two clustering approaches and since REEF is the more standard approach, we have based our subsequent analysis and experiments on the REEF results (the list of the clusters and the genes that constitute each cluster are shown in Table 2).

**Figure 2:**
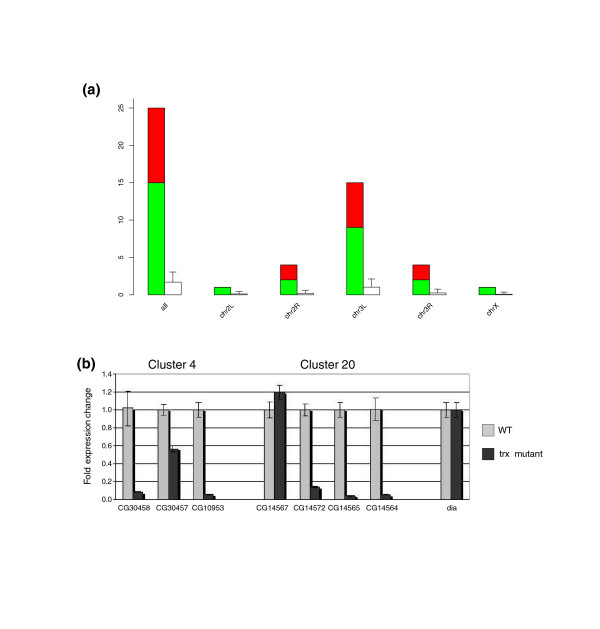
Specificity controls in the clustering process. **(a) **Statistical significance of clusters. Bar plots representing the number of clusters observed in the set of genes regulated by TRX (up-regulated clusters in red, down-regulated clusters in green) and the number of clusters expected by chance (in white). The number of *trx *clusters observed in each chromosome was highly significant (Z-score >2). Error bars represent the standard deviation of the random samples. **(b) **Quantitative RT-PCR of target expression (clusters 4 and 20) in third instar wild-type (WT) and *trx *mutant larvae. Error bars represent variability between replicates.

As a control test to assess the statistical significance of the clustering, we repeated the analysis on 100 sets of genes that were randomly selected from the fly genome, preserving the gene distribution in the chromosomes that we observed in the set of genes deregulated in *trx *mutant larvae (see Materials and methods). The number of clusters identified on the random sets was very small (on average 1.7 clusters compared with the 25 clusters observed from the experimental data) despite containing the same proportion of genes on every chromosome (Figure 2a). In addition, we computed the Z-score of the number of clusters observed in our microarray, using the distribution of number of clusters found in the random sets as background distribution (see Materials and methods). This score is highly significant for *trx *clusters: 17.25 (Additional data file 4). Because of the small size of clustered genes, one could argue that the clustering described here is due to specific properties of short and active genes, and not related to a trxG characteristic. Therefore, we retrieved all small genes of the fly genome (that is, genes with the same range of sizes as the ones found in this work) and repeated the previous test (see Materials and methods). The number of clusters observed in the whole collection of fly small genes was significant: 107 clusters (including 21 of the 25 *trx *clusters; Z-score 9.75; Additional data file 4). The existence of clusters of small sized and active genes has already been established for many genomes and it is thought that this organization could favor coordinated and efficient gene expression [42,43]. However, the clustering tendency of genes regulated by TRX is stronger as the Z-score for *trx *clusters (17.25) clearly contrasts with the one measured in the whole fly genome (9.75). As an additional control, we generated 100 random sets of genes preserving the same size distribution observed in up-regulated and down-regulated genes (see Materials and methods). The number of clusters detected in *trx *deregulated genes is highly significant (10 and 15 clusters, respectively) in comparison to the average number of clusters identified on these random gene sets (0.9 and 1.4 clusters). This is strongly indicative that the clustering tendency observed here is a specific characteristic of TRX regulated genes, and not a general feature of short genes (Additional data file 5).

In the analysis presented here, we have used no information about homology between genes within clusters to control for overrepresentation of gene families. Many genomic clusters corresponding to gene families have indeed been previously identified [44,45]. Such genomic clusters could cause spurious co-expression because of probe cross-hybridization between highly similar genes. In fact, some of the clusters that we have computationally identified do contain members of the same gene family (Table 2). We have searched for regions of similarity between the sequences of the genes within each cluster but no significant pairwise sequence alignments were found for any cluster (see Materials and methods). Furthermore, we confirmed the reported change in the expression of these genes by quantitative real-time RT-PCR in two clusters (Figure 2b).

Finally, we used the specific set of 445 genes (302 RefSeq genes) that are basally expressed in larvae described by Arbeitman *et al*. [28] to measure the specificity of our results (see Materials and methods). We were not able to reproduce in this data set the organization in clusters found in genes regulated by TRX (only one potential cluster was found), indicating that this is not a general feature of the larval stage in *D. melanogaster *development.

### Chromosomal clustering of genes controlled by other chromatin regulators

To determine whether the chromosomal organization in clusters is also common to genes regulated by other proteins involved in chromatin dynamics, we performed a second microarray expression experiment with mutant larvae for another such factor, ASH2, and compared the results obtained in this experiment, as well as previous published results on the transcriptomes of NURF [46], dMyc [47] and ASH1 [34], with the results obtained in the microarray analysis of the *trx *mutant. In all experiments, deregulated genes have been clustered on the *D. melanogaster *genome using the REEF program (Additional data file 6).

The *ash2 *gene (*absent*, *small*, *or homeotic discs 2*) is another member of the trxG involved in chromatin-mediated maintenance of transcription [48,49]. The microarray analysis identified 244 genes showing a statistically significant change (at least 2-fold change, *p*-value <0.05) in their expression between mutant and wild-type samples (see Materials and methods). According to their pattern of regulation, we identified 123 over-expressed genes and 121 under-expressed genes in the mutant larvae (Additional data file 7). As in previous studies [32,33], we found the same proportion of up-regulated and down-regulated genes in the *ash2 *mutants. We also mapped these genes to the genome of *D. melanogaster *according to the RefSeq annotations in the UCSC genome browser, and identified eight clusters of co-expressed genes (six clusters of up-regulated genes and two clusters of down-regulated genes) using the program REEF (Table 3).

**Table 3:** Clusters of genes regulated by different chromatin regulators

Microarray	Genes ↑	Genes ↓	Clusters ↑	Clusters ↓	Clusters ↑+↓	Clusters 3L	Reference
Trithorax	260	275	10	15	25	15	-
ASH2	123	121	6	2	8	4	-
NURF	-	265	-	7	7	4	[46]
dMyc	203	-	6	-	6	4	[47]
ASH1	239	159	7	1	8	2	[34]
							
Rovers	127	38	2	0	2	0	[57]
Sitters	131	112	2	1	3	1	[57]

For each microarray we display: number of up-regulated genes, number of down-regulated genes, number of up-regulated clusters, number of down-regulated clusters, total number of clusters, number of clusters located in chromosome 3L, bibliographical reference.

NURF is an ISWI-containing ATP-dependent chromatin remodeling complex [50]. Badenhorst *et al*. [46] performed a microarray analysis using larvae from *D. melanogaster *lacking the NURF specific subunit NURF301. We mapped the list of 274 genes (265 RefSeq genes) that require NURF301 according to this experiment (the list of up-regulated genes has not been published) to the genome. We then identified seven clusters of down-regulated genes using the program REEF (Table 3).

Goodliffe *et al*. [47] reported that the Polycomb protein (Pc), a member of PcG, mediates Myc autorepression and its transcriptional control at many loci. In this study the authors used the Gal4 UAS system to express ectopic *dmyc *in embryos and performed microarray analysis to examine the effect on gene expression. We mapped the list of 272 genes (203 RefSeq genes) up-regulated in this experiment (the list of down-regulated genes is unavailable) and then identified 6 clusters of co-expressed genes using the program REEF (Table 3).

More recently, Goodliffe *et al*. [34] extended the studies on Myc function and reported a coordinated regulation of Myc trans-activation targets by Pc and ASH1. The *ash1 *gene (*absent*, *small*, *or homeotic discs 1*) is also a member of the trxG [48]. In this work, the authors used RNAi to reduce the levels of *ash1 *and conducted microarray experiments [34]. The analysis of these microarrays identified 398 genes with a substantial change in their expression (239 over-expressed RefSeq genes and 159 under-expressed RefSeq genes). We mapped these genes to the fly genome and identified eight clusters of co-expressed genes (seven clusters of up-regulated genes and one cluster of down-regulated genes) using the program REEF (Table 3).

**Figure 3:**
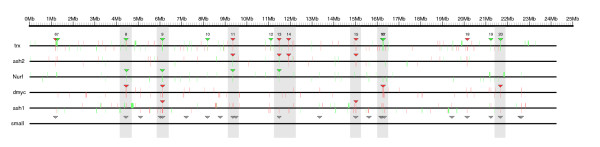
Genomic map of clusters of genes on chromosome 3L that are regulated by several chromatin regulators. The location of each gene reported on every microarray is indicated with a vertical line (up-regulated genes in red, down-regulated genes in green). Genes in the forward strand are displayed above the chromosome line, genes in the reverse strand are displayed below. Clusters of genes in each experiment are indicated with a triangle in red or green according to their expression. Clusters present in two or more microarrays are highlighted by gray bands. Clusters of small genes identified along the fly genome are denoted with a triangle in gray.

Together, these results suggest that chromosomal organization in clusters is a distinctive feature of some genes controlled by chromatin regulators. To elaborate more on this hypothesis, we compared the clusters identified in the microarray experiments of *trx *with those identified in the experiments of the other factors at three different levels: common clusters, common genes in clusters and common genes in the transcriptome maps (see Materials and methods for further details). We consider that two clusters from two different microarrays are matching if and only if they are overlapping in at least one commonly deregulated gene. The results of the comparison are shown in Table 4 and, as an example, the regulatory gene profiles of *trx*, *ash2*, *Nurf*, *dmyc *and *ash1 *along the chromosome 3L and the clusters containing these genes are shown in Figure 3 (the regions of the chromosome harboring the same cluster at the same time in both the *trx *experiment and another microarray are indicated with gray).

**Table 4:** Comparison between the clusters identified in different microarrays

Microarray 1	Microarray 2	Common genes	Common genes in clusters	Common genes in common clusters	Common clusters	Common clusters 3L
Trithorax	ASH2	76 (20%)	17 (27%)	17 (75%)	6 (75%)	4 (100%)
Trithorax	NURF	55 (14%)	10 (17%)	10 (45%)	4 (57%)	3 (75%)
Trithorax	dMyc	43 (12%)	13 (20%)	13 (41%)	6 (100%)	4 (100%)
Trithorax	ASH1	52 (11%)	9 (15%)	9 (38%)	4 (50%)	2 (100%)
						
Average		14%	20%	50%	71%	94%

Each line contains the following information about the comparison between the *trx *microarray and a second microarray: number of up- and down-regulated genes reported in common, number of common genes in clusters, number of common genes in common clusters, number of common clusters, number of common clusters in chromosome 3L.

Overall, between 50% (ASH1) and 100% (dMyc) of the *trx *clusters are also detected in the other chromatin regulators (71% on average; Table 4). This strongly suggests that there is high concordance between the *trx *clusters and the clusters inferred for the other chromatin regulators. There is not, however, an exact equivalence: clusters from different regulators that overlap in genome space with *trx *clusters may contain different regulated genes. Thus, the intersection between the genes deregulated by TRX and the genes regulated by other factors in the common clusters ranges from 38% (ASH1) to 75% (ASH2) of the genes (50% on average; Table 4). Nevertheless, this value dramatically decreases when the whole transcriptomes of each experiment are taken into account. In this case, the intersection between the set of genes deregulated in *trx *mutant larvae and any other set of genes whose expression was significantly affected by other chromatin regulators is lower than 20% on average (Table 4). These results suggest that the clusters identified in common form a group of gene targets directly or indirectly regulated by these chromatin regulators. In addition, this clustering is a specific feature of short and active genes: the average length of deregulated genes in these clusters is 1,135 bp, while the size of deregulated genes in these microarrays that are not clustered is, on average, 4,204 bp (Additional data file 8). These clusters overlap with clusters of small genes identified along the fly genome in the previous section: 75% of them for ASH2, 57% for NURF, 83% for dMyc, 75% for ASH1 (see Figure 3 for a graphical comparison on chromosome 3L).

The clustering organization reported here might be general for transcription factor target genes, and not a feature of genes regulated by chromatin remodeling factors. To rule out this hypothesis, we have collected microarray data for six transcription factors to extend the clustering analysis: *fkh *(fork head) [51], *ey *(eyeless) [52], *spdk *(spotted-dick) [53], *gcm *(glial cells missing) [54], *Otd *(Orthodenticle) [55] and *lab *(labial) [56]. We mapped each set of genes to the fly genome, using the program REEF to identify putative clusters. In most cases, however, no clusters were detected (Additional data file 9), indicating that clustering is not a general characteristic of transcription factor target genes. The lack of clustering in these microarrays does not merely reflect the larger gene size for the targets of these genes (Additional data file 10).

Finally, we used the expression data published by Riedl *et al*. [57] as a negative control to qualitatively assess the significance of our results. The information has been obtained from two microarray experiments involving rover and sitter larvae to study foraging locomotion in the fruit fly [57]. The intersection between these transcriptomes and the *trx *transcriptome is only slightly lower than that observed between TRX and the other chromatin regulators (6% and 9% for rover and sitter, respectively). However, only five clusters in total were detected among the genes regulated in the rover and sitter microarrays (2 and 3 clusters, respectively). Of these, only one mapped to chromosome 3L and none overlapped the *trx *clusters (Table 3).

### Analysis of co-expressed genes that constitute the clusters

**Figure 4:**
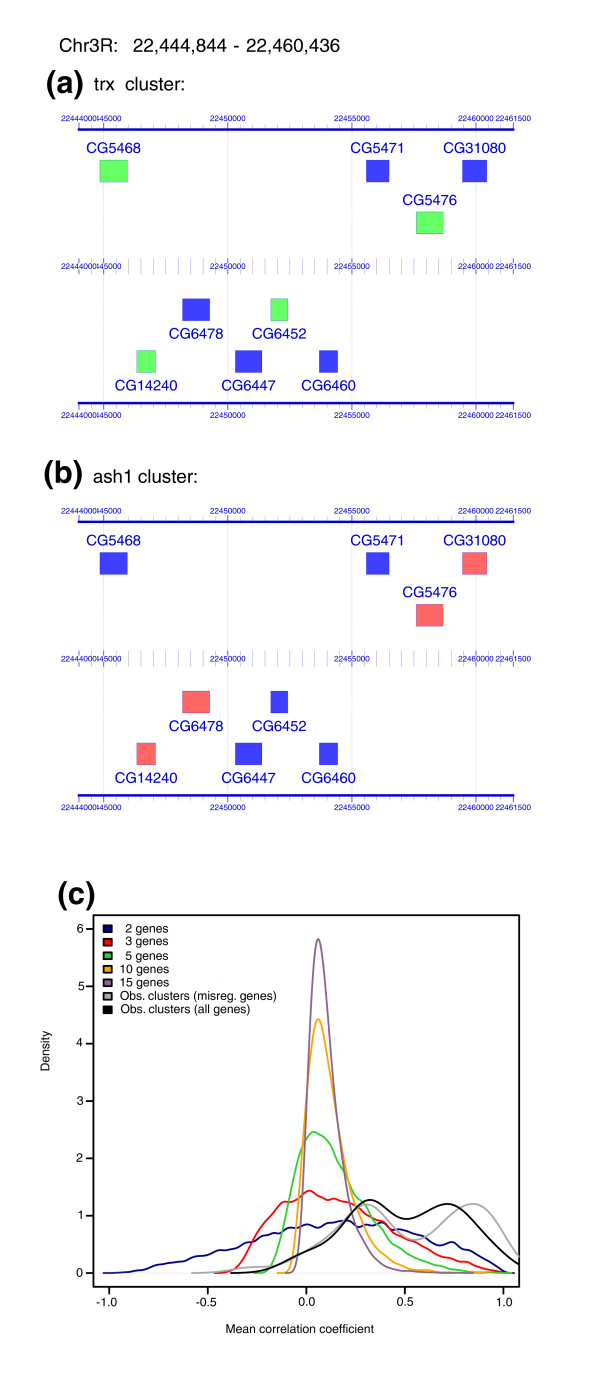
Co-expression of genes in clusters. **(a,b) **Expression of genes in the same cluster in different microarrays. (a) Cluster of four down-regulated genes (in green) in *trx *microarrays. (b) Cluster of four up-regulated genes (in red) in *ash1 *microarrays. Notice the boundaries and the co-regulated genes of the cluster are not the same in both experiments. These images were produced using the program GFF2PS [102]. **(c) **Graphical comparison between co-expression of genes in *trx *and artificial clusters, according to the expression data provided in [30]. For each cluster, the co-expression level was computed as the mean of Pearson's correlation coefficient between all pairs of genes in the cluster. The distribution of co-expression values within the boundaries of the *trx *clusters (including all genes or only the deregulated ones) is clearly skewed to the right, indicating much stronger co-expression than expected at random.

The genomic structure of the gene clusters governed by chromatin regulators does not appear to be homogeneous. The average size of clusters in the *trx *mutants is 3.5 genes, while the genomic region that harbors such genes contains, on average, 6.7 genes (Additional data file 2). For instance, although the cluster shown in Figure 4a contains four genes down-regulated by TRX (depicted in green), there are five additional genes annotated in this genomic region (depicted in blue) for which no change in expression was detected in the microarray. In addition, the comparison of the clusters identified in the different microarrays indicated that, as already outlined, only about 50% of the genes in a cluster regulated by either TRX or another chromatin regulator are actually deregulated in both experiments at the same time (Table 4). In many cases, therefore, either genes in the equivalent clusters from different experiments do not show the same regulation pattern or the boundaries of the clusters are not exactly the same. For example, the same cluster containing eight genes shown in Figure 4a, b was identified by the program REEF in both the *trx *and the *ash1 *microarrays. However, there are three interesting differences: the gene boundaries of the clusters when considering only the regulated genes are not the same; the expression of the genes changes in the opposite sense (down-regulation versus up-regulation); and some of the clustered genes are not regulated by any of the factors.

We used the whole-genome expression data generated by Hooper *et al*. [30] to investigate whether all genes within the genomic expanse of the *trx *clusters, and not only those defining the clusters themselves, are co-expressed (there are 162 genes within the region of the *trx *clusters, but only 97 in the clusters). For this dataset, Hooper *et al*. measured the expression of genes during the first 24 hours of embryonic development in *D. melanogaster *(1 hour time points). We used the data between 4 h and 24 h to minimize the possibility that the maternal effect could mask zygotic expression (see Materials and methods). Co-expression was evaluated both by using only those genes that define the *trx *clusters and using all genes located within the boundaries of each cluster. Based on the expression data provided in [30], we computed the Pearson's correlation coefficient between each pair of genes within the same chromosome across the 20 time points. For each cluster, the level of co-expression was then defined as the mean of Pearson's correlation coefficients between all pairs of genes in the cluster (see Materials and methods). As a reference set, we calculated the same values for each possible artificial cluster of N consecutive genes in the genome (2 ≤ N ≤ 15).

The distribution of values obtained for the clusters containing only the genes deregulated in *trx *mutants, the clusters containing all genes mapped within the boundaries of the clusters and the artificial clusters of several sizes using the 4 h-24 h expression data set are shown in Figure 4c. Interestingly, the distribution of co-expression levels in randomly generated clusters of different sizes appears to be slightly positive (means ranging from minimum to maximum), probably suggesting an overall induction of transcription during the first stages of larval development. The distribution of co-expression levels computed within the boundaries of clusters, and, in particular, computed only from the regulated genes defining the clusters, is, however, clearly skewed to the right, indicating much stronger coexpression than expected at random. The bimodal shape of the distribution, more accentuated when considering only the genes defining the clusters, suggests the existence of a class of clusters with tight regulation of expression. The deviation from randomness in the *trx *clusters is perhaps more appreciable in the cumulative plots (Additional data file 11).

Therefore, genes present within the genomic boundaries of the *trx *clusters, including those not in the defined clusters, are overall co-expressed. There are several causes that can explain the existence of additional genes within the boundaries of a *trx *cluster. These genes might not have been included in the clusters either because they were not in the array (4 cases out of 65 additional genes), because the gene showed a different pattern of regulation (up-regulated instead of down-regulated or vice versa, 1 case), or because the expression intensity from the microarray was below the selected thresholds (60 cases).

### Clusters may contain both up- and down-regulated genes

**Figure 5:**
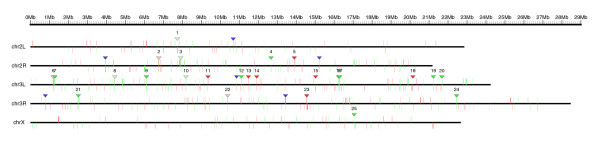
Genomic map of 'hybrid' clusters of genes deregulated by TRX in *D. melanogaster*. Computational identification of clusters was performed on a set of up- and down-regulated genes in the microrray. The new hybrid clusters of genes are indicated with a blue triangle. The clusters detected before - using one of both sets - are indicated with a red triangle (up-regulated genes) or a green triangle (down-regulated genes). Some of them have been enriched using genes expressed in the opposite sense (displayed in light red or light green).

The trxG members are known to be positive regulators of transcription [24]. However, in our study, we found a similar number of up-regulated compared to down-regulated genes in the *trx *mutants. Similar results have recently been reported for *ash2*, *ash1 *and *Nurf301 *[33,34,46], suggesting that trxG proteins might also act directly or indirectly as repressors of certain genes. We once more applied the REEF clustering strategy, but this time considering all *trx *deregulated genes together, irrespective of the direction of their regulation. In addition to the 25 clusters previously detected, this method allowed us to identify six additional 'hybrid' clusters (with both up- and down-regulated genes). Moreover, we also enriched previously detected clusters with genes regulated in the opposite direction (Figure 5). In total, we identified 129 deregulated genes that were organized in 31 clusters.

### The chromosomal clustering is conserved in other species

The clusters of genes detected here might be acting as transcriptional units with coordinated transcriptional regulation. One would therefore expect some level of conservation of cluster organization across species. The genomes of multiple species of *Drosophila *have been recently made available through the UCSC genome browser [38], allowing investigation of the conservation of *trx *clusters in other *Drosophila *species. Only three of these genomes have been completely assembled: *D. simulans*, *D. yakuba *and *D. pseudoobscura *[58]. We have mapped all *D. melanogaster *genes to the genomes of each of these species using the BLAT alignments provided by the UCSC genome browser [59] (see Materials and methods). The number of genes annotated on each species using this method is shown in Table 5.

**Table 5:** Clusters of genes deregulated in *trx *mutants conserved in other phylogenetically related species

		Genes (orthologs)					
						
Species	Genome	↑	↓	Clusters ↑	Clusters ↓	Clusters	Deregulated genes in clusters
***D. melanogaster***	19,670	260	275	10	15	25	97
*D. simulans*	17,927	235	262	7	13	20	75
*D. yakuba*	19,929	259	275	11	14	25	96
*D. pseudoobscura*	15,096	170	230	1	13	14	60
*A. gambiae*	7,283	97	151	2	5	7	34

For each species, we show: number of genes for which an ortholog in *D. melanogaster *was found, number of orthologs for up-regulated genes, number of orthologs for down-regulated genes, number of up-regulated clusters, number of down-regulated clusters, total number of clusters, number of deregulated genes from *D. melanogaster *that constitute the clusters.

**Figure 6:**
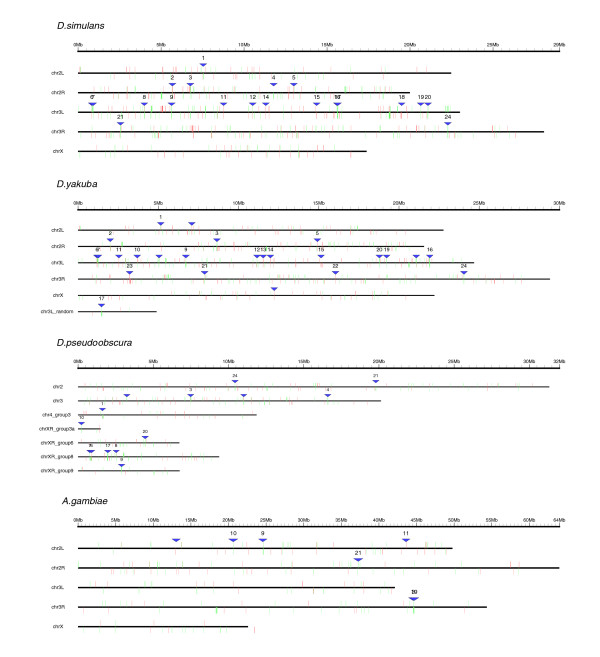
Genomic map in other species of clusters deregulated in *trx *mutants. The location in each species of the orthologous gene deregulated in *D. melanogaster *is indicated with a vertical line (up-regulated genes in red, down-regulated genes in green). Genes in the forward strand are displayed above the chromosome line, genes in the reverse strand are displayed below. Clusters of genes identified on each genome are indicated with a blue triangle.

After mapping the up-regulated and down-regulated genes of the *trx *mutant from *D. melanogaster *to the other *Drosophila *genomes, we used the program REEF with the same set of parameters to identify putative clustering of these genes. The number of clusters detected in these species is shown in Table 5: 20 clusters in *D. simulans *(corresponding to 7 up-regulated clusters and 13 down-regulated clusters in the *trx *microarrays), 25 clusters in *D. yakuba *(11 up-regulated clusters, 14 down-regulated clusters) and 14 clusters in *D. pseudoobscura *(1 up-regulated cluster, 13 down-regulated clusters). We have compared the clusters obtained in *D. melanogaster *with the clusters identified in these three species: 24 out of 25 clusters (96%) identified in *D. melanogaster *were conserved in at least one other species (80% of the clusters were conserved in *D. melanogaster *and two more species, 36% of the clusters were conserved in all species). In contrast, the percentage of clusters identified in these species that was not detected in *D. melanogaster *was very low (0% in *D. simulans*, 16% in *D. yakuba*, 14% in *D. pseudoobscura*; Table 6), indicating that this set of deregulated genes is similarly organized in the genome of these species. The distribution of clusters on each genome is shown in Figure 6 (the clusters of *D. melanogaster *that are conserved in other species have the same identifier as in Figure 1).

**Table 6:** Conservation of genes in the clusters and their vicinity

Genome	No. of clusters	No. of clusters conserved in *D. melanogaster*	No. of genes within the clusters	% Genes conserved within the clusters	% Genes conserved in the flanking area	% Genes conserved in artificial clusters	Divergence time estimates (Mya)
*D. simulans*	20	20 (100%)	144	96%	86%	90%	2-3
*D. yakuba*	25	21 (84%)	146	88%	64%	79%	5-7
*D. pseudoobscura*	14	12 (86%)	83	96%	58%	65%	25-55
*A. gambiae*	7	6 (86%)	48	66%	2%	20%	250-300

The following information is shown for each genome: the species, the number of clusters predicted, the number and the percentage of clusters that are conserved in *D. melanogaster*, the number of genes in the clusters (the same amount of genes is used to measure the conservation in the flanking areas), the percentage of genes that are conserved between these clusters and the corresponding clusters in *D. melanogaster*, the percentage of genes that are conserved in the flanking areas of the clusters (average conservation in the left and right flanking areas), the percentage of the genes that are conserved in 10,000 artificial clusters sampled on each species, and the divergence time (million years ago (Mya)) estimates between *D. melanogaster *and each species, extracted from [58,101].

Another genome of interest for the identification of homologous clusters potentially regulated by the *trx *gene is that of *Anopheles gambiae *[60]. We obtained the list of putative *Anopheles *orthologs to the *D. melanogaster *genes using the ENSEMBL annotations [61]. Less than 50% of the fly genes could be mapped to the mosquito genome in this way (Table 5). Consequently, only 7 clusters were identified. Most of these clusters, however, were conserved in *D. melanogaster *(Figure 6 and Table 6).

In the work presented here, we identified a set of 25 gene clusters in *D. melanogaster *that are phylogenetically conserved in other flies. However, given the strong synteny between the *Drosophila *genomes (see divergence time estimates in Table 6), we can not claim that the conservation of clusters that we observed is not simply a consequence of such an overall synteny. To discard such a hypothesis, for each cluster identified in *D. melanogaster *we examined the number of genes in common found in the corresponding cluster in each of the other *Drosophila *species (allowing for gene rearrangements and chromosome inversions inside the region; see Materials and methods for further details). We also analyzed the number of genes in common between the corresponding flanking areas of these clusters in order to compare the number of genes that are conserved inside and outside them; the results are shown in Table 6. While the genes that constitute the clusters of *trx *in *D. melanogaster *are mostly the same in the clusters of the other species (96% in *D. simulans*, 88% in *D. yakuba*, 96% in *D. pseudoobscura*), the number of conserved genes in the vicinity of each cluster decreases in more distant species (86% in *D. simulans*, 64% in *D. yakuba*, 58% in *D. pseudoobscura*). Additional statistical tests confirmed these observations (see Materials and methods). According to these results, we conclude that the overall synteny between the *Drosophila *genomes is not enough to explain the high level of conservation observed in the clusters of genes deregulated by TRX in *D. melanogaster*.

### Clusters of deregulated genes are enriched in some functional categories

In order to characterize the clusters previously identified in *D. melanogaster*, we functionally annotated their constituent genes (Additional data file 12) using Gene Ontology (GO) [62]. GO is a hierarchical dictionary of biological terms structured into three main categories: molecular function, biological process and cellular component. We also annotated the function of the full set of genes in our microarray and of the genes that were reported to be up-regulated or down-regulated to estimate the statistical significance of our results.

**Figure 7:**
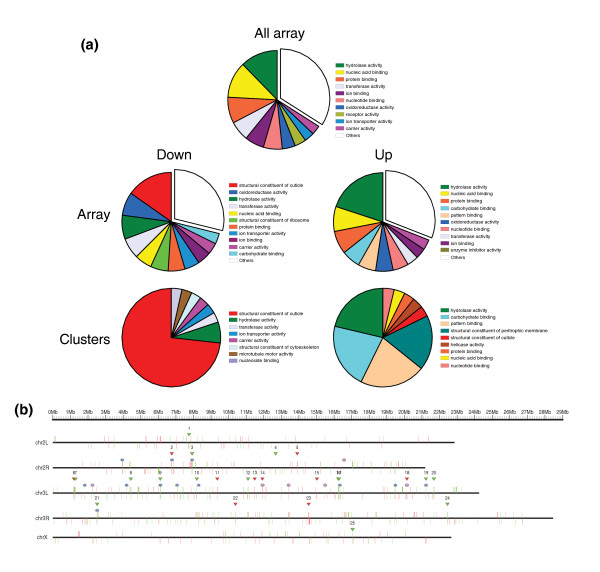
Functional annotation of genes deregulated in *trx *mutants. **(a) **Classification of the microarray gene set, the deregulated genes and the genes that constitute the clusters according to the GO category 'molecular function', level 3. **(b) **Genomic map of clusters of functionally related genes. Clusters of genes annotated as structural constituents of cuticle (displayed as blue stars) and clusters of genes annotated as chitin binding (displayed as purple circles). Clusters of co-regulated genes in the *trx *mutant are indicated with a triangle in red or green according to their expression. Notice that most functional clusters match regulatory clusters despite the fact that both approaches are completely different.

We analyzed the information available for the genes of each respective set (12,120 genes in the microarray, 535 deregulated genes, 97 genes in clusters) at the third level of the molecular function ontology (see Materials and methods). A graphical representation of the more abundant categories for each of the three gene sets is shown in Figure 7a. The clusters of down-regulated genes are significantly enriched in structural proteins involved in cuticle formation (*p*-value <10^-37^; see Materials and methods). The over-representation is less relevant in the set of down-regulated genes, while it is not observed in the full collection of genes in the microarray (Figure 7). The clusters of up-regulated genes are also enriched in proteins with carbohydrate and pattern binding functions, as well as structural components of the peritrophic membrane (*p*-value <0.005; see Materials and methods). The set of up-regulated genes is also, albeit to a lesser extent, enriched in these categories, while no over-representation is observed in the complete set of genes in the microarray (Figure 7). A more detailed inspection of the functional annotations of the clusters reveals that this over-representation is due to the abundance of genes involved in the subcategory 'chitin binding' [62].

According to this, the clusters of genes deregulated in the *trx *mutant contain a significant number of genes involved in cuticle structure and other related functions. To confirm these results, we performed a whole-genome clustering of the genes from *D. melanogaster *according to their functional annotation in GO, in which no expression data were used (see Materials and methods). We focused, in particular, on the two functional categories over-represented in the clusters of genes deregulated in *trx *mutants: structural constituents of cuticle (GO:0042302) and chitin binding (GO:0008061).

We found 98 genes annotated as components of the cuticle in the genome of *D. melanogaster*. Using the program REEF with the same set of parameters, we identified 12 clusters of genes involved in cuticle structure (shown as blue stars in Figure 7b), 8 of which are located on chromosome 3L. We found 67 genes annotated as chitin binding proteins and identified 6 clusters, 5 of which are also located on chromosome 3L (shown as purple circles in Figure 7b). Of the 18 functional clusters, 8 overlap the clusters of genes regulated by TRX (shown as red and green marks in Figure 7b). In addition, most genes in the functional clusters are annotated as being in the same functional categories (Additional data file 13).

### Clusters of genes deregulated in *trx *mutants are enriched in tissue specific genes

**Figure 8:**
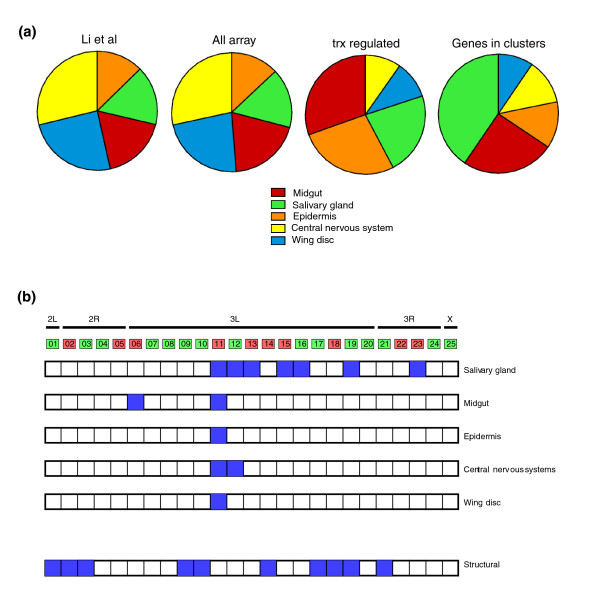
Clusters are enriched in genes expressed in particular tissues. **(a) **From left to right: tissue distribution of genes expressed 18 h before larval to pupal metamorphosis according to Li *et al*. [63]; expression pattern of genes included in our *trx *microarray; genes deregulated by TRX; and genes in clusters deregulated by TRX. **(b) **Tissue distribution of clustered genes (at least one gene must be expressed in that tissue). Clusters that contain genes annotated as structural proteins in GO are displayed for comparison.

We have also attempted to characterize the expression pattern of genes in the *trx *clusters. Using data from the Li *et al*. [63] study, in which the tissue distribution of genes expressed 18 hours before the larval to pupal metamorphosis in *D. melanogaster *was determined, we characterized the expression of the members of three different gene sets (genes in the microarray, deregulated genes, genes in clusters; see Materials and methods for further details); results are shown in Figure 8a. As expected, the proportion of genes in our microarray expressed in different tissues is very similar to that reported by Li *et al*. [63]: 28% are expressed in the central nervous system, 24% in wing discs, 20% in the midgut, 16% in salivary glands and 12% in the epidermis. Most genes regulated by TRX, on the other hand, are expressed in the midgut (30%, *p*-value <10^-5^) and in the epidermis (27%, *p*-value <10^-6^), while the genes that constitute the clusters regulated by TRX are abundantly expressed in salivary glands (41% of genes, *p*-value <10^-5^). We compared the tissue-specific expression pattern of the genes in the *trx *clusters with the functional annotation of these genes as inferred through the GO annotations (see previous section) and results are shown in Figure 8b: 16 of 25 clusters (64%) contain genes that are either expressed in salivary glands or code for structural cuticle proteins. In addition, the clusters appear to be divided into those containing genes expressed in salivary glands and those containing genes coding for structural proteins. Together, these results demonstrate that TRX plays a key role in the regulation of clusters containing genes involved in the structure of the cuticle during the larval stages of *D. melanogaster *development.

### PRE/TRE predictions and experimental approaches

The PcG and trxG complexes bind to sequences called PRE/TREs. However, not only is the genomic distance between well-characterized PRE/TREs and their target genes highly variable, ranging from a few nucleotides to more than 60 Kb in many cases, but they can be found both upstream or downstream of the gene [64-66]. Several methods have been proposed to detect PRE/TRE elements in genomic sequences. For example, Ringrose *et al*. [67] developed a computational approach to detect potential PRE/TREs and identified 167 candidates in the fly genome, some of which were validated experimentally [67]. More recently, several ChIP-on-chip experiments have been performed to search for PcG targets. Among others, Schwartz *et al*. [68] determined the distribution of the PcG proteins Pc, E(z) and Psc and of H3K27me3 in the whole genome, Tolhuis *et al*. [69] constructed a map of binding patterns of the PcG proteins Pc, esc and Sce in chromosomes 2L and 4, and part of chromosomes 2R and X, and Negre *et al*. [70] analyzed the binding profile of the PcG proteins Pc and ph, and the GAGA factor in certain regions of chromosomes 2L and X.

**Figure 9:**
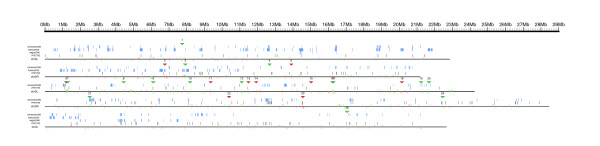
Genomic map of clusters, PcG ChIP-on-chip data and predicted PRE/TREs. The location of each gene reported on the *trx *microarray is indicated with a vertical line (up-regulated genes in red, down-regulated genes in green). Genes in the forward strand are displayed above the chromosome line, genes in the reverse strand are displayed below. Clusters of genes on each experiment are indicated with a triangle in red or green according to their expression. PcG binding domains reported by Schwartz *et al*. [68], Tolhuis *et al*. [69] and Negre *et al*. [70] are displayed in blue. PRE/TRE predictions obtained by Ringrose *et al*. [67] are displayed in black.

We mapped the PcG target sites identified in each of these experiments (251 sites in Schwartz *et al*. [68], 131 sites in Tolhuis *et al*. [69] and 36 sites in Negre *et al*. [70]) and the 167 PRE/TREs predicted by Ringrose *et al*. [67] in the fly genome (assembly dm2, April 2004). We then compared the location of the 25 clusters of genes deregulated by TRX in the fruit fly genome with the location of the PcG target sites and the PRE/TRE predictions (Figure 9). Since the distance between the PRE/TRE and its target gene can be highly variable, we decided to confine the search to the PcG binding regions or PRE/TREs that were located at most 100 Kb distant from our clusters. According to this restriction, we found 14 out of 25 clusters (56%) near one experimental evidence or a PRE/TRE prediction, five of which were supported by both a PcG binding site and a PRE/TRE (Additional data file 14). We performed ChIP analysis of third instar larvae, using both anti-TRX and H3K4me3 specific antibodies, to test the possible binding of the TRX protein to some of the predicted PRE/TREs [67] that are in close proximity to the *trx *deregulated clusters. Preliminary results seem to indicate that TRX is capable of binding to at least two of the PRE/TREs tested (Additional data file 15). In keeping with its role as a histone methyltransferase, TRX binding correlates with the presence of H3K4me3.

## Discussion

In this study we have compared the whole-genome expression profiles of *trx *mutant and wild-type fly larvae and located the deregulated genes on the fly genome. Considering the stringent threshold used in this study, it is possible that the total number of regulated genes may be underestimated. In the absence of genome-wide ChIP data, we can not assess TRX binding and its putative relationship with H3K4 trimethylation, but taking into account that most active genes have this mark in most species, it is tempting to speculate that the TRX protein itself or another member of this group should be responsible for it. Nevertheless, the genome mapping revealed the tendency of some *trx *deregulated genes to cluster within a few genomic locations with 97 genes (about 20% of all deregulated genes) organized in 25 genomic clusters covering less than 400,000 bp (about 0.3% of the fly genome). This appears to be a distinctive feature of the regulatory networks of other chromatin regulators. Indeed, our microarray experiments on *ash2 *mutants, as well as experiments performed on NURF [46], dMyc [47] and ASH1 [34], indicate that the genes regulated by these chromatin factors are also clustered in the fly genome. Remarkably, these clusters are a subset of the *trx *deregulated clusters, despite that only about 20% of the *trx *deregulated genes are also deregulated in these other experiments. In fact, the number of genes in clusters is likely to be also underestimated, because of the thresholds used to establish deregulation. Moreover, when using the time course whole-genome expression data during the first 24 hours of embryonic development [30], we observe an overall positive correlation between the expression levels of all genes within the boundaries of the *trx *clusters (irrespective of whether they are included in the clusters themselves). Similar results are observed when using the transcription maps generated across the fly genome in the same developmental stage by Manak *et al*. [71]. In fact, most genes in the *trx *clusters present similar patterns of overlapping transcribed fragments (transfrags; Additional data file 16). In addition, we found a significant under-representation of non-exonic transfrags within our clusters (the observed overlap between transfrags and clusters was 100 bp and the expected overlap was 2,610.8 bp, *χ*^2 ^homogeneity test, *p*-value = 0). Since the presence of transfrags in the vicinity of genes is often an indication of alternative transcription initiation and termination sites, their under-representation suggests that the boundaries of the genes in these clusters are under tight transcriptional control.

Some clusters detected here have been previously described in the literature. For instance, clusters 9 (Lcp65A, larval cuticle proteins) and 21 (Ccp84, cuticular genes cluster), both down-regulated in the *trx *microarray, are two well-known groups of genes involved in the determination of the physical characteristics of the insect cuticle [72,73]. Other interesting examples are clusters 11 (*Hsp23*, *Hsp26*, *Hsp27*) and 13 (*Sgs3*, *Sgs7*, *Sgs8*), which are up-regulated in *trx *deficient flies and down-regulated in flies lacking *ash2 *or *Nurf301*. *Hsp23*, *Hsp26 *and *Hsp27 *are heat shock genes also expressed in the absence of stress during embryogenesis and metamorphosis in *D. melanogaster *but their role in these processes is not well understood [74,75]. *Sgs3*, *Sgs7 *and *Sgs8 *form a cluster of genes that code for proteins that are part of the salivary glue secreted by *Drosophila *larvae to fix themselves to an external substrate for the duration of the pre-pupal and pupal periods [76,77]. Both clusters were previously reported to be regulated by the 20E- inducible Broad-Complex (BR-C), an early ecdysone response gene complex that is active during larval to pupal metamorphosis and encodes a family of zinc-finger transcription factors [78,79]. Although *Sgs *expression is known to be indirectly controlled by BR-C [80], up-regulation of *br *(a member of BR-C) in *trx *mutants could explain the up-regulation of both clusters. Consistent with this hypothesis, *br *has a reduced level of expression in *ash2 *and *Nurf301 *mutants, where such clusters are down-regulated, suggesting BR-C can be an intermediate step in the regulation of the expression of *Sgs *and *Hsp *clusters. Homeotic genes are also clustered in the genome and their expression state is maintained by PcG and trxG proteins after the initial transcriptional regulators disappear from the embryo [27]. In our *trx *mutant microarrays most homeotic genes did present a decrease in expression; however, the difference was inferior to the fold change threshold selected to filter and normalize the expression data. In addition, homeotic complexes are substantially larger than the *trx *clusters reported in our work, which suggests that the computational tools used here should be reconfigured to detect them.

We compared the results of several ChIP-on-chip experiments on different fragments of the fruit fly genome that have already been published [68-70] and the set of computational PRE/TRE predictions obtained by Ringrose *et al*. [67] with the location of our clusters. The analysis of this information is, however, difficult due to the different conditions in each experiment, the genome coverage of them and the limited knowledge of the PRE/TRE sequences [26]. In contrast to vertebrates, it seems that only around 30% of PcG binding sites are within 2 Kb of a promoter in flies, complicating the assignment to specific genes [81]. Half of the gene clusters deregulated in *trx *mutants are close (less than 100 Kb) to functional PcG binding sites or PRE/TRE predictions, suggesting that some clusters may be directly regulated by TRX, while others can be under indirect regulation. A recent study presented evidence for the existence of arrays of highly conserved non-coding elements (HCNEs) and genomic regulatory blocks in five *Drosophila *species [82], giving rise to some controversy about whether PRE/TREs might be found inside those regions or not [26]. We mapped the HCNEs identified by Engstrom *et al*. [82] to our clusters (Additional data file 17), detecting a significant under-representation of such elements in comparison to the rest of the genome (the observed overlap between HCNEs and clusters was 190 bp, and the expected overlap was 1,342.7 bp, *χ*^2 ^homogeneity test, *p*-value = 0).

Multiple genome-wide approaches for the detection of clustering organization have been published (see Hurst *et al*. [8] for a review), and a variety of computational approaches towards that end have been developed [20,40,41,83]. Comparison of the results, however, is complex because of several reasons: the lack of a standard definition of a cluster (from series of two/three adjacent genes to groups of 10-30 genes), the statistical engine is different, even in the same species, different sets of genes were used, or the biological conditions (tissues/developmental stage) of the study are not comparable. The clusters presented here are located in relatively small regions of the genome (average cluster size of 15,918 nucleotides) in contrast to larger clusters reported in previous studies (cluster length between 20 and 200 kb in [20]). In addition, we tolerate within the boundaries of the genomic clusters the presence of genes that are not shown to be regulated in the microarray data (only adjacent co-expressed genes were accepted in clusters in [20,21]).

The growing body of evidence supporting the existence of non-random gene distribution in genomes [8] indicates that genome organization may be partially evolutionarily conserved across eukaryotes. Phylogenetically conserved clusters of co-expressed genes have recently been reported in human and mouse [84,85], while adjacent pairs of essential genes that are evolutionarily conserved have been shown to cluster in regions of low recombination [86]. In fact, neighboring genes are thought to experience continuous concerted expression changes during evolution, which can lead to the formation of clusters of co-expressed genes. However, the pattern of expression might be evolving slowly within the clusters. On the other hand, some clusters may be maintained by natural selection because of their similar biological functions [87]. Recently, a comparative analysis of the genomes of 12 *Drosophila *species has been published [88,89]. Here, we identified a set of 25 clusters in *D. melanogaster *that are phylogenetically conserved in *D. simulans*, *D. yakuba *and *D. pseudoobscura*. The conservation that we have observed in these clusters is stronger than the overall synteny among the *Drosophila *genomes. Our preliminary results on *A. gambiae*, which diverged from *Drosophila *about 250 million years ago, are in support of the selective constraints to maintain the cluster organization.

Lee and Sonnhammer [90] recently reported the existence of a link between functional pathways in the KEGG dabatase [91] and the chromosomal distribution of genes involved in them. Here, we functionally annotated the genes constituting the clusters of genes deregulated in the *trx *mutant using GO [62], and detected a significant enrichment in structural proteins involved in cuticle formation and chitin metabolism. To assess the importance of this overrepresentation, we extracted all genes of the fruit fly genome annotated with these functions and used the program REEF to identify potential clusters on this set of genes. Remarkably, there is a high overlap between the map of functional clusters and the map of regulatory clusters. The overrepresentation of structural proteins involved in cuticle formation suggests that the trxG proteins analyzed here may play a role in the hormonal response that takes place during metamorphosis. Indeed, a function for the histone methyltransferase protein TRR (Trithorax-related) as a coactivator for the ecdysone receptor [92] and a direct link between NURF and ecdysteroid signaling in larval to pupal metamorphosis [46] have been reported. If genes involved in larval/pupal transition, such as the ones described here, are among the trxG targets, a putative explanation for the preferential localization in chromosome 3L may be just that 67% of the functionally annotated clusters of genes involved in cuticle structure are located in this chromosome. Moreover, it is known that many mutations affecting trxG genes (either null alleles or heteroallelic combinations) are lethal at the third instar larval stage, probably due to a large dowry of maternally loaded mRNA, and do not undergo larval to pupal metamorphosis. In spite of other phenotypic differences between these larvae, it is tempting to speculate that lethality could be caused by desiccation due to defective cuticle secretion and that the trxG/PcG regulation of the clusters could have a pivotal role in metamorphosis.

## Conclusion

Further experiments correlating gene expression states and chromatin modifications in specific tissues during development as well as chromatin protein binding maps will be required to understand the complex role of trxG proteins.

## Materials and methods

### *Drosophila *strains

All *Drosophila *strains and crosses were kept on standard media with 0.025% bromophenol blue. The reference line used was the *w*^*1118*^_*iso*_; *2*_*iso*_; *3*_*iso *_isogenic line from the DrosDel Collection [93]. To reduce the differences in the biological background between the alleles under study and the reference strain, we transferred chromosomes X, Y and 2 from the isogenic line to the TM6c-balanced *ash2*^*I1*^, and to *trx*^*B11*^ and *trx*^*E3*^ alleles, which were used in trans-heterozygosity. Their genotypes were, respectively, *w*^*1118*^_*iso*_; *2*_*iso*_; *ash2*^*I1*^/*TM6c*, *w*^*1118*^_*iso*_; *2*_*iso*_; *trx*^*B11*^/*TM6c *and *w*^*1118*^_*iso*_; *2*_*iso*_; *trx*^*E3*^/*TM6c*.

### Microarray design

Microarrays were printed on Corning UltraGAPS slides (Corning, Corning, NY, USA) at the Plataforma de Transcriptòmica (SCT - PCB, Universitat de Barcelona, Spain) using the *Drosophila *Genome Oligo Set version 1.1 (Operon Biotechnologies Inc., Huntsville, AL, USA), a collection of 14,593 probes representing 13,577 *Drosophila *genes with Flybase ID (12,120 genes in RefSeq). The 70 mer *Arabidopsis *sequences from TIGR [94] and spots with no material or with buffer were also printed to be used as spike-in and negative controls, respectively. The microarray annotation is deposited in the Gene Expression Omnibus database with accession number GPL3797. Wandering blue-gut staged Tb+ early third instar larvae were selected in all cases to extract total RNA using the RNeasy Protect Mini Kit (Qiagen Inc., Valencia, CA, USA). At least two independent total RNA extractions were carried out. Quality was assessed in all samples using the Eukaryote Total RNA Nano Assay on a 2100 Bioanalyzer (Agilent Technologies Inc., Santa Clara, CA, USA). Total RNA from *w*^*1118*^;+;+ larvae was used as a common reference. Four microarrays were hybridized for each experiment in biological replicate pairs, such that the total RNA from the sample used as a starting material came from different extractions. Both arrays from each pair were hybridized with the same amplified RNA from sample and common reference (obtained using the Amino-Allyl Messageamp II aRNA Amplification Kit from Ambion Inc., Austin, TX, USA) using dyes Cy3 and Cy5 (GE Healthcare UK Ltd, Buckinghamshire, UK).

### Microarray analysis

GenePix Results (GPR) data files were obtained for each microarray with an Axon 4000B scanner and GenePix Pro 6 (Molecular Devices Corp., Sunnyvale, CA, USA). All GPR files were analyzed with the Limma package from BioConductor [95,96] using the same criteria. The spots not fulfilling the quality thresholds (based on spot size, foreground versus background signals, saturation, coincidence between differently calculated ratio measures and R^2 ^of regression ratio) were eliminated from the analysis, and the data were background corrected with the *normexp *method and normalized using OLIN [97]. Between-array normalization was carried out independently for each set of four arrays using the *mad *method from OLIN, and a linear model was fitted and corrected with False Discovery Rate (FDR) [95]. We obtained lists of genes that were differentially expressed two-fold in the mutants compared to the isogenic strain (FDR-corrected *p*-value lower than 0.05). Raw and normalized data are deposited in the Gene Expression Omnibus database (user name: sergiba_rev_1, password: 2139861083) with the accession number GSE8783, which includes the data for *trx *(GSE8748) and *ash2 *(GSE8750) mutants.

### Microarray data collection

We obtained the set of genes that are differentially expressed in the larvae of *D. melanogaster *from Arbeitman *et al*. [28]. We selected only the genes that were up-regulated at least two-fold in the larvae (302 RefSeq genes). We extracted the gene expression data for *Nurf301-/- *from Badenhorst *et al*. [46] (only the list of down-regulated genes was published). The set of up-regulated genes in response to *dmyc *over-expression was taken from Goodliffe *et al*. [47]. We extracted from Goodliffe *et al*. [34] the set of up and down-regulated genes when the regulatory activity of *ash1 *was repressed using RNA interference to study the interaction between the dMyc complex and ASH1. We used the expression data from two microarray analyses (rovers and sitters) about foraging locomotion from Riedl *et al*. [57] as a negative control in the clustering process. The set of genes expressed in five different tissues (midgut, salivary glands, epidermis, central nervous system and wing disc) studied in the larval stage of *D. melanogaster *was obtained from Li *et al*. [63].

### Real-time RT-PCR analysis

Reverse transcription reactions with RNA independently isolated in Trizol from all mutant alleles and the reference were used to synthesize cDNA with M-MLV RT (Invitrogen Corp., Carlsbad, CA, USA) according to the manufacturer instructions. We used 1 *μ*l of a 1/10 dilution of cDNA on each quantitative real-time PCR (qRT-PCR). The qRT-PCR was performed on the ABI PRISM^® ^7700 following the recommended protocol (Applied Biosystems, Foster City, CA, USA). Each sample was replicated three times and average values were used for further analysis. Data were analyzed by the ΔΔC_T _method, being normalized by subtracting the value of the control gene *dia*. TaqMan primers and probes designed and synthesized by Applied Biosystems for this analysis were: Dm02371023_s1 (CG30458); Dm02366349_s1 (CG30457); Dm02366353_s1 (CG10953); Dm01792445_s1 (CG14567); Dm01792458_s1 (CG14572); Dm01792469_s1 (CG14565); Dm01792478_s1 (CG14564); and Dm01811206_g1 (*dia*).

### Chromatin immunoprecipitation

ChIP assays were essentially performed as previously described by Papp and Muller [98] with the following changes: 40 wandering third instar larvae were fixed with 1.8% formaldehyde solution for 25 minutes at room temperature, collected in 700 *μ*l of lysis buffer (1% SDS, 50 mM Tris HCl pH 8.0 and 10 mM EDTA) and disrupted 6 times for 20 seconds at 30% of a Branson sonifier. Samples were centrifuged at top speed at 4°C and aliquotes of 100 *μ*L of extract were used per immunoprecipitation. Ten percent of the immunoprecipitated extract was used as input, and a sample immunoprecipitated without antibody as a precipitation control. A 300 bp fragment corresponding to the transcription start of the *Ultrabithorax *gene, which has been previously described to present a TRX binding site [98,99], was used as a positive control. TRX antibody was a gift from A Mazo and H3K4me3 antibody was obtained from Abcam Inc. The primers used for the PCR amplifications were: Ubx forward, 5' CATGCCCAGCGAGAGAGG 3'; Ubx reverse, 5' AACAGCACAGAAAGCGAGG 3'; cluster9 forward, 5' ACCCACTTTTGCGCCATCG 3'; cluster9 reverse, 5' ACAAAGCGGTTCCGTGTCG 3'; cluster25 forward, 5' ACGTCTGGCTATGGATCTGG 3'; cluster25 reverse, 5' GGACACCGATGTGACCACC 3'.

### Gene mapping in the genome

All gene sets were mapped in the genome of *D. melanogaster *using the RefSeq track of the UCSC genome browser [38] annotations (genome assembly dm2, April 2004). We considered only one transcript per gene (the first one present in the annotations). The file refGene.txt was used to retrieve the coordinates of each gene in RefSeq. The official name and the description of each gene were retrieved from the file refLink.txt. The statistics of gene distribution on each chromosome were computed using the 19,670 unique RefSeq genes contained in the file refGene.txt as well. To measure the statistical significance of the gene distribution, we randomly generated 1,000 rounds of sets of genes with the same size to evaluate the *p*-value of each result using a hypergeometric distribution. We used the BLAT alignments between the RefSeq genes from *D. melanogaster *and the genomes of *D. simulans*, *D. yakuba *and *D. pseudoobscura *to produce a catalogue of genes for these species (Additional data file 18). On each genome distribution, the file xenoRefGene.txt contains the best BLAT hits of the genes of other species in that genome [38]. We selected only the best hit for each RefSeq gene from *D. melanogaster *in the corresponding genome distributions of *D. simulans*, *D. yakuba *and *D. pseudoobscura*. The sets of up- and down-regulated genes in the *trx *microarray were then mapped in those genomes using the appropriate catalogue of genes. We used the list of orthologous genes between *D. melanogaster *and *A. gambiae *provided by the Biomart tool [100] from ENSEMBL. The association between the ENSEMBL gene identifier and the official gene name in the fruit fly genome was used to cross this information with the set of RefSeq genes of fly annotated in the UCSC.

### Cluster identification

We define a cluster as a group of neighboring genes located in a limited region of the genome, not necessarily consecutive, that show the same expression pattern (up-regulation or down-regulation) in the microarray experiment. Clusters are determined by the physical position in the genome, the number of co-expressed genes inside and the total number of genes in such a genomic fragment. The length of the clusters can be then defined in terms of nucleotides or number of genes annotated on that region. We follow two similar approaches to computationally detect the clusters in the sets of genes in our work: the program REEF and our own implementation of a clustering program. No significant difference was observed between the results obtained with both computational approaches.

To evaluate the significance of the number of clusters identified in our microarray, we generated 100 randomly generated sets with the same size and chromosomal gene distribution of the real sets of genes regulated by TRX. We then performed the clustering analysis in such data sets to find out how many clusters could be obtained by chance (expected clusters). The Z-score of the results observed in our microarray was thus calculated using the distribution of clusters identified in the random sets:

$$z=\frac{x-\mu }{\sigma }$$

where *x *is the number of observed clusters in the *trx *microarray, and *μ *and *σ *are the mean and the standard deviation of the distribution of number of clusters found in the random sets, respectively.

To evaluate whether regulation by TRX imposes a stronger clustering tendency compared to gene size, we extracted all genes from the fruit fly genome whose size is within the range delimited by the size observed in genes from *trx *clusters (average size 946 bp, standard deviation 788 bp). We thus mapped the 6,626 genes that match this condition on the genome to get their coordinates and identified 107 clusters, using the REEF program. In order to assess the statistical significance of the clustering (and to measure which parameter, *trx *regulation or gene size, is more discriminant in the clustering), we repeated the analysis on 100 sets of genes that were randomly selected, preserving the gene distribution observed in the dataset of short genes. As an additional control, we also counted how many clusters can be identified in 100 different random sets of 260 genes (number of up-regulated genes) and 275 genes (number of down-regulated genes), which preserve the same gene size distribution observed in both lists of deregulated genes in our *trx *microarray (average and standard deviation).

The program REEF [41] identifies regions of a genome enriched in specific features, compared with a reference landscape of feature density. The two input files are: the list of reference features (genes annotated in the fly genome) mapped on a genome sequence; and the list of selected features among the reference set (genes regulated by TRX in our microarray) with their genomic positions and the number and the length of the chromosomes in the genome under consideration. REEF scans the reference genome using a sliding window approach, calculating the statistical significance of each window using the hypergeometric distribution and the FDR. The windows are defined as fragments of a fixed genomic length. Consecutive significant windows form a cluster of regional enriched features. Using an approach similar to that used by Spellman and Rubin [20], we defined the optimal set of parameters comparing the growth function in the number of clusters obtained in random sets and in the real reference set for different values (Additional data file 19). The optimal configuration of parameters to scan the genome of *D. melanogaster *was: window length, 25,000 bp; window step, 1,000 bp; minimal number of co-expressed genes, 3; q-value ≤0.05s.

### Cluster identification based on gene density

We have also implemented our own approach in Perl to cluster the genes regulated by a certain gene. Basically, we scan the genome following a sliding window approach combined with the hypergeometric distribution to evaluate the statistical significance of the clusters. The length of the windows is determined, however, in terms of genes rather than nucleotides. Thus, the clustering process is not limited by a fixed window length. Some parameters in this algorithm must be adjusted in order to maximize the sensibility and specificity. Firstly, only clusters of at least three genes showing the same sense of deregulation were considered. Another important parameter is the size of the window to apply. Using the results in random sets, a window size of seven genes was selected as the optimal length (the number of clusters found in *trx *data seems to grow logarithmically with the size of the window used, while the number of clusters found in random sets grows exponentially). We used our algorithm to scan the chromosomes of *D. melanogaster *using window lengths of seven genes, advancing one gene between two instances of the window so that all possible seven-gene windows were tested. To avoid multiple testing problems, we took a conservative *p*-value threshold of 10^-3^. Consecutive statistically significant windows were merged up in only one cluster. Results obtained with our clustering strategy are highly concordant to those produced by the REEF program (Additional data file 3): 27 clusters were detected (22 identical clusters, 2 clusters with additional genes, 3 new clusters and 1 missing cluster).

### Clustering characterization and comparison

We computed different values on the genes that constitute the clusters (using the file refGene.txt downloaded from the UCSC genome browser): the average gene length, the gene length distribution, the number of bidirectional/opposed genes on each cluster according to their strand, and the average intergenic distance. Such values were compared to those observed in the whole set of genes annotated in the fruit fly genome, to evaluate their significance. The comparison between the clusters of genes regulated by TRX and the clusters regulated by other chromatin regulators was performed at three different levels: common genes in the transcriptomic maps, common genes in clusters and genomic position of the clusters. The intersection between two transcriptomes is defined as the quotient between the number of common genes (twice) and the total number of genes in both experiments. Two clusters from two different microarrays are matching if and only if they are overlapping in at least one commonly deregulated gene. Thus, we calculated the clusters identified in a second experiment (*ash2*, *Nurf*, *dmyc*, *ash1*) that were supported by another cluster in the *trx *microarray results. Once a set of common clusters was identified in two microarrays, we computed the percentage of co-expressed genes that was present in each pair of equivalent clusters (the ratio between the number of common genes and the total number of genes on each cluster).

Two clusters of genes identified in two different genomes are considered to be equivalent when the percentage of genes that are present in both clusters is 50% or higher. To calculate the percentage of genes that are common in two clusters, the genes that are located in a different relative order within the clusters are considered to be conserved. Clusters of genes that are conserved in different strands of the chromosomes are considered to be equivalent as well (allowing for chromosomal inversions in the flanking regions). The length of the left and right flanking areas of each cluster is equal to the number of genes of the corresponding cluster. To measure the statistical significance of these results, we randomly sampled 10,000 artificial clusters of seven genes in *D. melanogaster *(the average size of our clusters is 6.7 genes) and found the location of the equivalent cluster on the other genomes. We examined the number of genes shared between each artificial cluster of genes in *D. melanogaster *and its equivalent cluster conserved on each of the other *Drosophila *species. Each artificial cluster on each genome was constituted of the corresponding gene and the three genes annotated before and after in the same location (the average length of our clusters is 6.7 genes). The results are shown in Table 6. The conservation of the artificial clusters in terms of common genes is more similar to the conservation observed in the flanking area of our clusters (90% in *D. simulans*, 79% in *D. yakuba*, 65% in *D. pseudoobscura*). In fact, the difference between the number of conserved genes inside our clusters and inside the artificial clusters is significant in all the species (*D. simulans p*-value <10^-3^, *D. yakuba p*-value <10^-3^, *D. pseudoobscura p*-value <10^-6^; Wilcoxon test). Similar results were obtained in the set of clusters detected in *A. gambiae *(Table 6).

### Gene co-expression in clusters

Hooper *et al*. [30] generated whole-genome expression data for the first 24 h of embryonic *D. melanogaster *development by extracting RNA from overlapping 1 h time points for the first 6.5 h of development and non-overlapping 1 h time points for the rest. We generated a table containing all pairwise Pearson's correlation coefficients between all genes expressed between 4 and 24 h in the study by Hooper *et al*. [30]. For each cluster identified in the *trx *mutants, we computed the mean of all pairwise correlation coefficients between the genes constituting the cluster to assess if they are co-expressed along the embryonic development. We also calculated the mean, including all the genes included in the boundaries of each cluster. As a reference set, we computed the same value for each set of correlative N genes in the genome (2 ≤ N ≤ 15). We then evaluated if each particular cluster was located in any of these tails and the total percentage of clusters that were found in these tails.

### Functional annotations

We downloaded the GO terms and relationships (file gene_ontology_edit.obo, release 1.2) and the associations between gene products from *D. melanogaster *and GO terms (file gene_association.fb, release 1.94) from the GO web site [62]. We annotated different gene sets (full set of genes in the *trx *microarray, the up-regulated and down-regulated genes in the same experiment, the clusters identified in such co-expression sets) using the annotation available at level 3 of the 'molecular function' ontology tree. We climbed up in the ontology to obtain the corresponding parent term at level 3 for those genes that were annotated at deeper levels in the hierarchical tree. The statistical analysis was performed using hypergeometric distribution to test the probability of observing a given GO term significantly enriched in genes belonging to such clusters:

$$p=\sum_{i=n}^{min\left[k,a\right]}\frac{\left(\begin{array}{c} A\\ i \end{array}\right)\left(\begin{array}{c} G-A\\ k-i \end{array}\right)}{\left(\begin{array}{c} G\\ k \end{array}\right)}$$

In this case, *G *is the number of up- or down-regulated genes, *A *is the subset of *G *that are annotated in that GO term, *k *is the number of genes up- or down-regulated in clusters, and *n *is the subset of *k *annotated in the GO term.

We performed a whole-genome clustering of the genes from *D. melanogaster *annotated with the hierarchies based on the term structural constituents of cuticle (GO:0042302). No data about gene expression in the microarrays were used here. We first extracted the fly genes annotated with the following GO terms: structural constituent of cuticle (GO:0042302); structural constituent of chitin-based cuticle (GO:0005214); structural constituent of chitin-based larval cuticle (GO:0008010); structural constituent of pupal chitin-based cuticle (GO:0008011); structural constituent of adult chitin-based cuticle (GO:0008012); and structural constituent of collagen and cuticulin-based cuticle (GO:0042329). Then, we used the program REEF to identify clustering organization events in this gene set (the same configuration of parameters set in the case of co-expressed genes). We also performed a whole-genome clustering of the genes from *D. melanogaster *annotated with the hierarchies based on the term chitin binding (GO:0008061). We first extracted the fly genes annotated with the following GO terms: chitin binding (GO:0008061); polysaccharide binding (GO:0030247); carbohydrate binding (GO:0030246); and pattern binding (GO:0001871). Then, we used the program REEF to identify clustering organization events in this gene set.

## Abbreviations

BR-C, Broad-Complex; FDR, false discovery rate; GO, Gene Ontology; HCNE, highly conserved non-coding element; Pc, Polycomb protein; PcG, polycomb group; PRE, Polycomb response element; qRT-PCR, quantitative real-time PCR; TRE, Trithorax response element; trxG, trithorax group.

## Authors' contributions

EB, MP and RG conceived the bioinformatics experiments. EB, MP and SB performed the bionformatics analysis. SB, AP and MC designed the microarray experiments. SB performed the microarray analysis. AP and SP performed the qRT-PCR experiments. SP designed and performed the chromatin immunoprecipitation experiments. FS and MC contributed reagents/material/analysis tools. EB, MP, SB, RG and MC wrote the paper.

## Additional data files

The following additional data files are available. Additional data file 1 lists the up- and down-regulated genes in the *trx *microarray. Additional data file 2 is a table of general features of the clusters of genes deregulated by TRX. Additional data file 3 is a table of the clusters detected using our own clustering approach. Additional data file 4 contains the results of clusters detected in random gene sets (gene distribution). Additional data file 5 contains the results of clusters detected in random gene sets (gene size). Additional data file 6 is a graphical representation of the different clusters detected in the genome that are deregulated by several chromatin remodelers. Additional data file 7 lists up- and down-regulated genes in the *ash2 *microarray. Additional data file 8 is a table of the average lengths of the deregulated genes on each microarray (clustered or not). Additional data file 9 is a table that shows the results of the clustering analysis in other microarrays of transcription factors. Additional data file 10 is a table that shows the average gene size of deregulated genes in the microarrays analyzed in this study. Additional data file 11 shows the cumulative distribution Pearson correlation coefficient means in the real and the artificial clusters. Additional data file 12 contains the GO functional annotation of the *trx *clusters. Additional data file 13 is a graphical representation of the clusters of genes in the genome associated with similar categories (cuticle/chitin binding). Additional data file 14 is a table that shows the intersection between the *trx *clusters, the ChIP-on-chip information and the PRE/TRE predictions. Additional data file 15 shows the results of the chromatin immunoprecipitation using anti-TRX and H3K4me3 specific antibodies to test the binding of TRX to the predicted PRE/TREs in clusters 9 and 25. Additional data file 16 shows several examples of clusters and transfrags that detect similar patterns of expression. Additional data file 17 is a graphical genome-wide representation of the clusters of *trx *and the HCNEs mapped in several *Drosophila *species. Additional data file 18 is a table that contains catalogs of orthologous genes between *D. melanogaster *and the other *Drosophila *species or mosquito. Additional data file 19 is an image that represents the optimal window length to discriminate between real and artificial clusters. Additional data file 20 is a table that shows the results of an alternative overlap analysis among the clusters of genes regulated by different chromatin remodelers (only genes affected in the same way are considered).

## Supplementary Material

Additional data file 1 Up- and down-regulated genes in the *trx *microarray. Click here for file

Additional data file 2 General features of the clusters of genes deregulated by TRX. Click here for file

Additional data file 3 Clusters detected using our own clustering approach. Click here for file

Additional data file 4 Clusters detected in random gene sets (gene distribution). Click here for file

Additional data file 5 Clusters detected in random gene sets (gene size). Click here for file

Additional data file 6 Different clusters detected in the genome that are deregulated by several chromatin remodelers. Click here for file

Additional data file 7 Up- and down-regulated genes in the *ash2 *microarray. Click here for file

Additional data file 8 Average lengths of the deregulated genes on each microarray (clustered or not). Click here for file

Additional data file 9 Clustering analysis in other microarrays of transcription factors. Click here for file

Additional data file 10 Average gene size of deregulated genes in the microarrays analyzed in this study. Click here for file

Additional data file 11 Cumulative distribution Pearson correlation coefficient means in the real and the artificial clusters. Click here for file

Additional data file 12 GO functional annotation of the *trx *clusters. Click here for file

Additional data file 13 Clusters of genes in the genome associated with similar categories (cuticle/chitin binding). Click here for file

Additional data file 14 Intersection between the *trx *clusters, the ChIP-on-chip information and the PRE/TRE predictions. Click here for file

Additional data file 15 Chromatin immunoprecipitation using anti-TRX and H3K4me3 specific antibodies to test the binding of TRX to the predicted PRE/TREs in clusters 9 and 25. Click here for file

Additional data file 16 Examples of clusters and transfrags that detect similar patterns of expression. Click here for file

Additional data file 17 Graphical genome-wide representation of the clusters of *trx *and the HCNEs mapped in several *Drosophila *species. Click here for file

Additional data file 18 Orthologous genes between *D. melanogaster *and the other *Drosophila *species or mosquito. Click here for file

Additional data file 19 The optimal window length to discriminate between real and artificial clusters. Click here for file

Additional data file 20 Alternative overlap analysis among the clusters of genes regulated by different chromatin remodelers (only genes affected in the same way are considered). Click here for file
