# Supplementary material for: Conserved chromosomal clustering of genes governed by chromatin regulators in Drosophila
Source: Genome Biol. 2008 Sep 10;9(9):R134. doi: 10.1186/gb-2008-9-9-r134 (PMC2592712; doi:10.1186/gb-2008-9-9-r134)
Supplement: Additional data file 2 — General features of the clusters of genes deregulated by TRX. [file gb-2008-9-9-r134-S2.pdf]

Chromosome	Clusters	Length	Avg. Length	Misexpressed genes	Avg. Misexpressed genes	Genes	Avg. Genes
2L	1	12,609	12,609	3	3.0	4	4.0
2R	4	62,892	15,723	15	3.7	30	7.5
3L	15	227,525	15,168	62	4.1	96	6.4
3R	4	45,137	11,284	14	3.5	22	5.5
X	1	24,804	24,804	3	3.0	10	10.0
TOTAL	25	372,967	15,918	97	3.5	162	6.7
