# Supplementary material for: Conserved chromosomal clustering of genes governed by chromatin regulators in Drosophila
Source: Genome Biol. 2008 Sep 10;9(9):R134. doi: 10.1186/gb-2008-9-9-r134 (PMC2592712; doi:10.1186/gb-2008-9-9-r134)
Supplement: Additional data file 3 — Clusters detected using our own clustering approach. [file gb-2008-9-9-r134-S3.doc]

TABLE 1

| Chr | Begin | End | Reg | pvalue | Genes regulated in cluster |
| --- | --- | --- | --- | --- | --- |
| 2L | 7732656 | 7767648 | D | <10^-4^ | Acp1 CG7214 CG7203 |
| 2R | 6752590 | 6777697 | U | <10^-5^ | CG9080 CG30029 CG7738 CG13224 |
| 2R | 7902885 | 7941364 | D | <10^-6^ | CG8505 CG8836 CG8510 CG8511 CG8520 |
| 2R | 12651360 | 12780415 | D | <10^-4^ | CG30458 CG30457 CG10953 |
| 2R | 13899427 | 13908524 | U | <10^-4^ | CG18107 CG16836 CG15068 |
| 3L | 1179852 | 1221845 | U | <10^-7^ | LysC LysB LysD LysP LysS |
| 3L | 1286215 | 1297227 | D | <10^-3^ | CG9149 CG2469 CG 9186 |
| 3L | 4419319 | 4452493 | D | <10^-3^ | CG12607 CG11345 CG32241 |
| 3L | 4819589 | 4957019 | D | <10^-3^ | CG32237 CG17150 CG13705 |
| 3L | 6068940 | 6134760 | D | <10^-6^ | CG32405 Lcp65Ag2 CG18779 l(3)mbn CG18778 Lcp65Ae CG32404 Lcp65Ac Lcp65Ab2 Lcp65Aa |
| 3L | 8176586 | 8234312 | D | <10^-3^ | CG8012 CG13674 CG13678 |
| 3L | 9346877 | 9376138 | U | <10^-3^ | Hsp26 Hsp23 Hsp27 |
| 3L | 11094722 | 11138921 | D | <10^-3^ | CG7628 CG32074 CG14143 |
| 3L | 11452520 | 11596660 | U | <10^-3^ | Sgs8 Sgs7 Sgs3 |
| 3L | 11898704 | 11946926 | U | <10^-3^ | CG5883 CG7252 CG17826 |
| 3L | 15014158 | 15049886 | U | <10^-3^ | CG13461 CG18649 CG13460 |
| 3L | 16227011 | 16244468 | D | <10^-3^ | CG13069 CG13068 CG13067 |
| 3L | 16247251 | 16295116 | D | <10-4 | CG13063 CG4982 CG13047 CG4962 CG13041 CG13060 CG13059 |
| 3L | 19453212 | 19490729 | D | <10^-3^ | CG32212 CG9283 CG9290 |
| 3L | 20139884 | 20155656 | U | <10^-3^ | CG7290 CG7017 CG6933 |
| 3L | 21219105 | 21251817 | D | <10^-3^ | CG11310 Edg78E CG7658 |
| 3L | 21662784 | 21742679 | D | <10^-6^ | CG14566 CG14569 CG14568 CG14572 CG14565 CG14564 |
| 3R | 2510171 | 2567785 | D | <10^-5^ | Ccp84Ag Ccp84Ad Ccp84Ab Ccp84Aa |
| 3R | 10346792 | 10401570 | U | <10^-4^ | CG14850 CG8087 CG14852 |
| 3R | 14518441 | 14584588 | U | <10^-6^ | CG7710 CG7714 CG7715 CG14302 |
| 3R | 2242393 | 22455578 | D | <10^-3^ | CG5468 CG14240 CG6452 |
| X | 21186099 | 21268410 | U | <10^-4^ | Hlc Ics CG10918 |
