# Supplementary figures and images for: Conserved chromosomal clustering of genes governed by chromatin regulators in Drosophila
Source: Genome Biol. 2008 Sep 10;9(9):R134. doi: 10.1186/gb-2008-9-9-r134 (PMC2592712; doi:10.1186/gb-2008-9-9-r134)

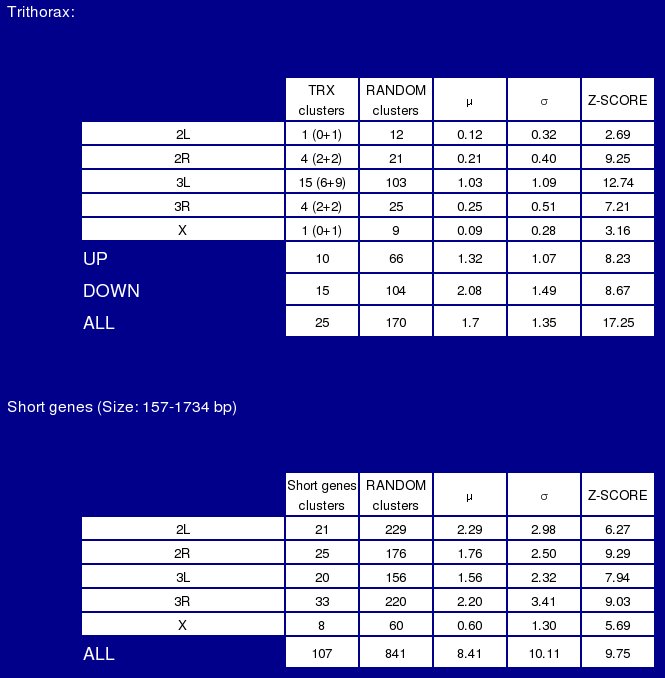

Supplement: Additional data file 4 — Clusters detected in random gene sets (gene distribution). [file gb-2008-9-9-r134-S4.jpeg]

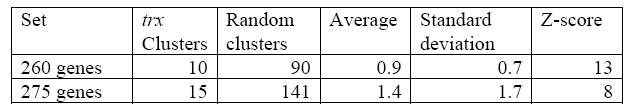

Supplement: Additional data file 5 — Clusters detected in random gene sets (gene size). [file gb-2008-9-9-r134-S5.jpeg]

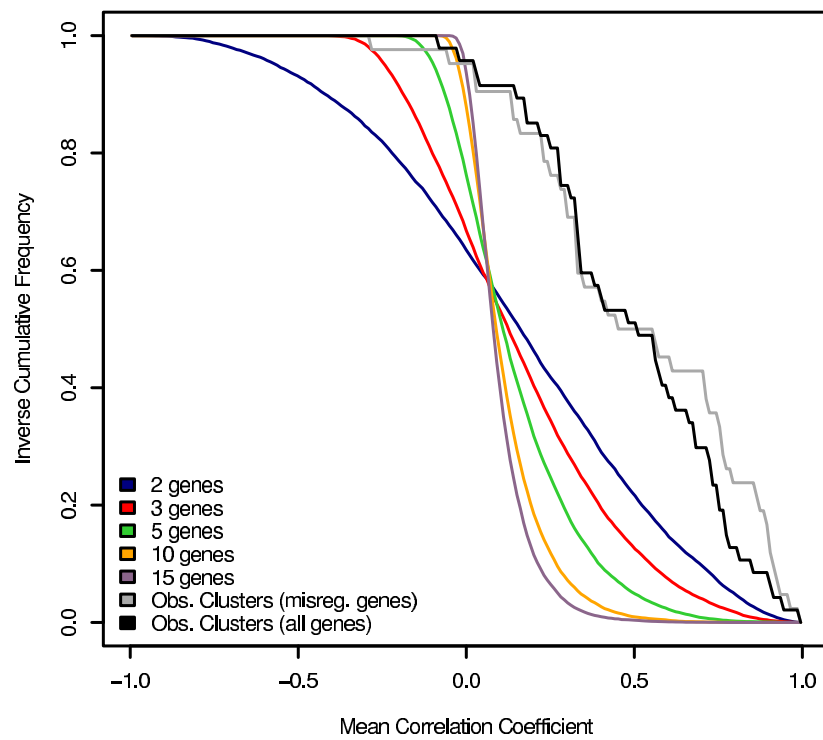

Supplement: Additional data file 11 — Cumulative distribution Pearson correlation coefficient means in the real and the artificial clusters. [file gb-2008-9-9-r134-S11.pdf]

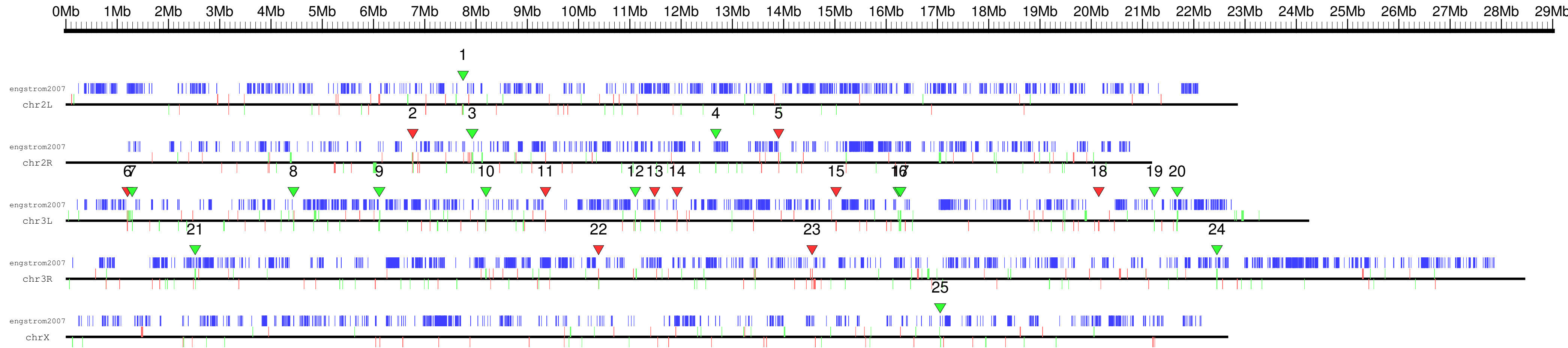

Supplement: Additional data file 17 — Graphical genome-wide representation of the clusters of trx and the HCNEs mapped in several Drosophila species. [file gb-2008-9-9-r134-S17.pdf]

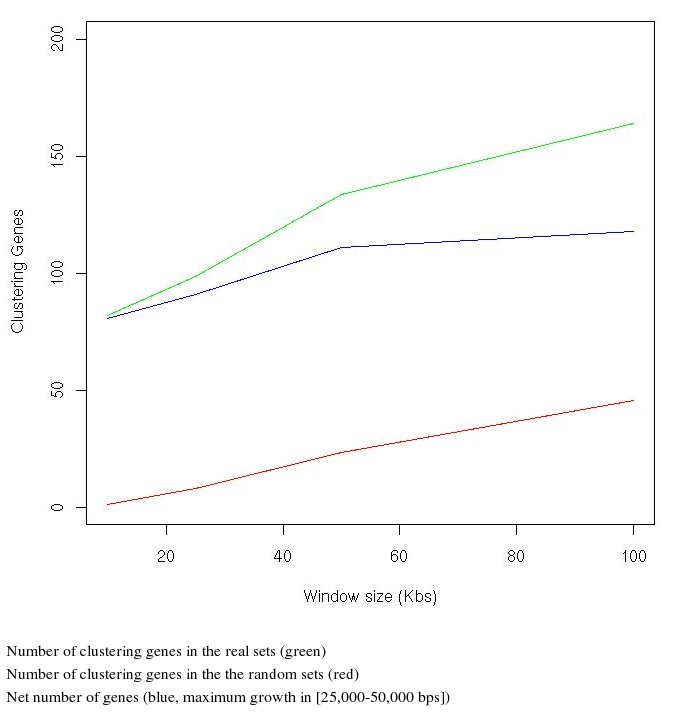

Supplement: Additional data file 19 — The optimal window length to discriminate between real and artificial clusters. [file gb-2008-9-9-r134-S19.jpeg]
