# Supplementary material for: Conserved chromosomal clustering of genes governed by chromatin regulators in Drosophila
Source: Genome Biol. 2008 Sep 10;9(9):R134. doi: 10.1186/gb-2008-9-9-r134 (PMC2592712; doi:10.1186/gb-2008-9-9-r134)
Supplement: Additional data file 6 — Different clusters detected in the genome that are deregulated by several chromatin remodelers. [file gb-2008-9-9-r134-S6.pdf]

trxU – chr3L: 1190902 - 1211770

Genomic components: 5 coregulated genes, 6 genes

Chr	Strand	Start	End	RefSeq	Name	Exons	Description
chr3L	+	1190902	1191400	NM_079158	LysB	1	Lysozyme B CG1179-PA
chr3L	+	1194016	1194503	NM_080130	LysC	1	Lysozyme C CG9111-PA
chr3L	-	1194256	1194728	NM_057475	LysD	1	Lysozyme D CG9118-PA
chr3L	+	1196455	1196938	NM_057479	LysE	1	Lysozyme E CG1180-PA
chr3L	-	1201613	1202228	NM_057480	LysP	1	Lysozyme P CG9116-PA
chr3L	+	1211299	1211770	NM_057481	LysS	1	Lysozyme S CG1165-PA

Cluster size: 20869 nucleotides

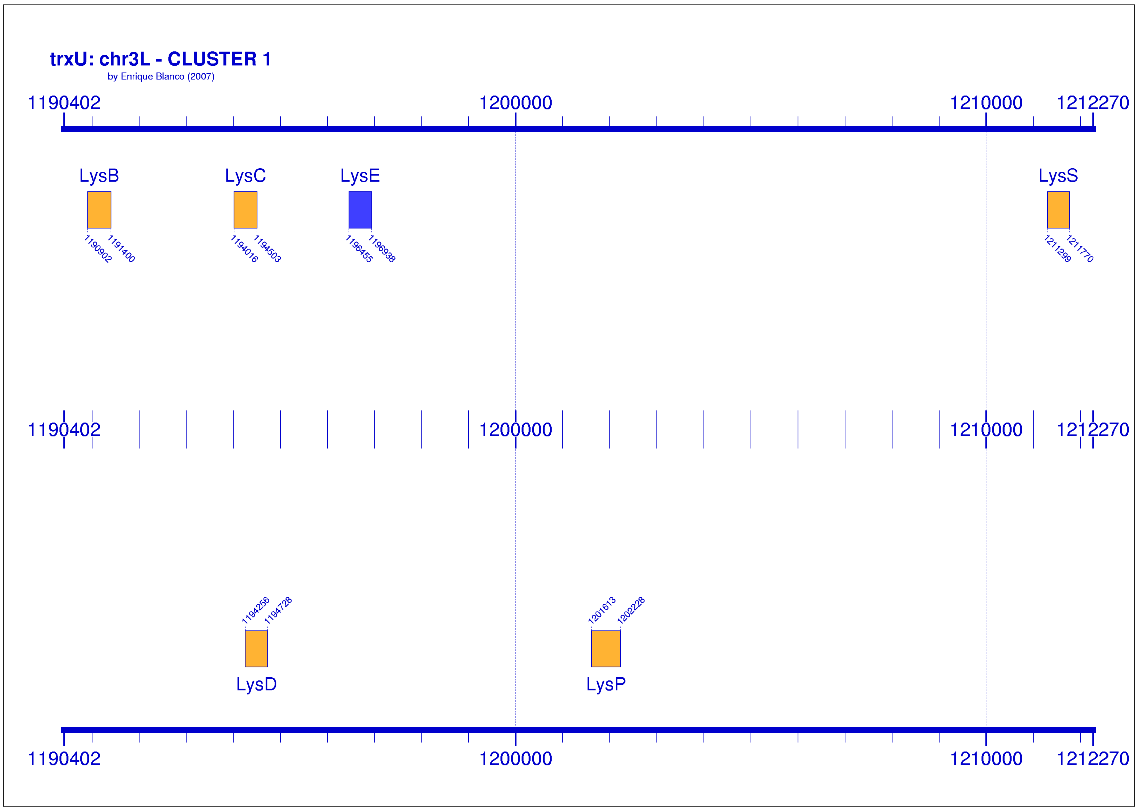

Enrique Blanco © 2007 — May 22, 2007

trxU – chr3L: 9350364 - 9359229

Genomic components: 3 coregulated genes, 4 genes

CHR	Strand	Start	End	RefSeq	Name	Exons	Description
CHR3L	-	9350364	9351374	NM_079273	Hsp26	1	Heat shock protein 26 CG4183-PA
CHR3L	-	9352049	9353386	NM_079274	Hsp67Ba	1	Heat shock gene 67Ba CG4167-PA
CHR3L	+	9355830	9356712	NM_079275	Hsp23	1	Heat shock protein 23 CG4463-PA
CHR3L	+	9358011	9359229	NM_079276	Hsp27	1	Heat shock protein 27 CG4466-PA

Cluster size: 8866 nucleotides

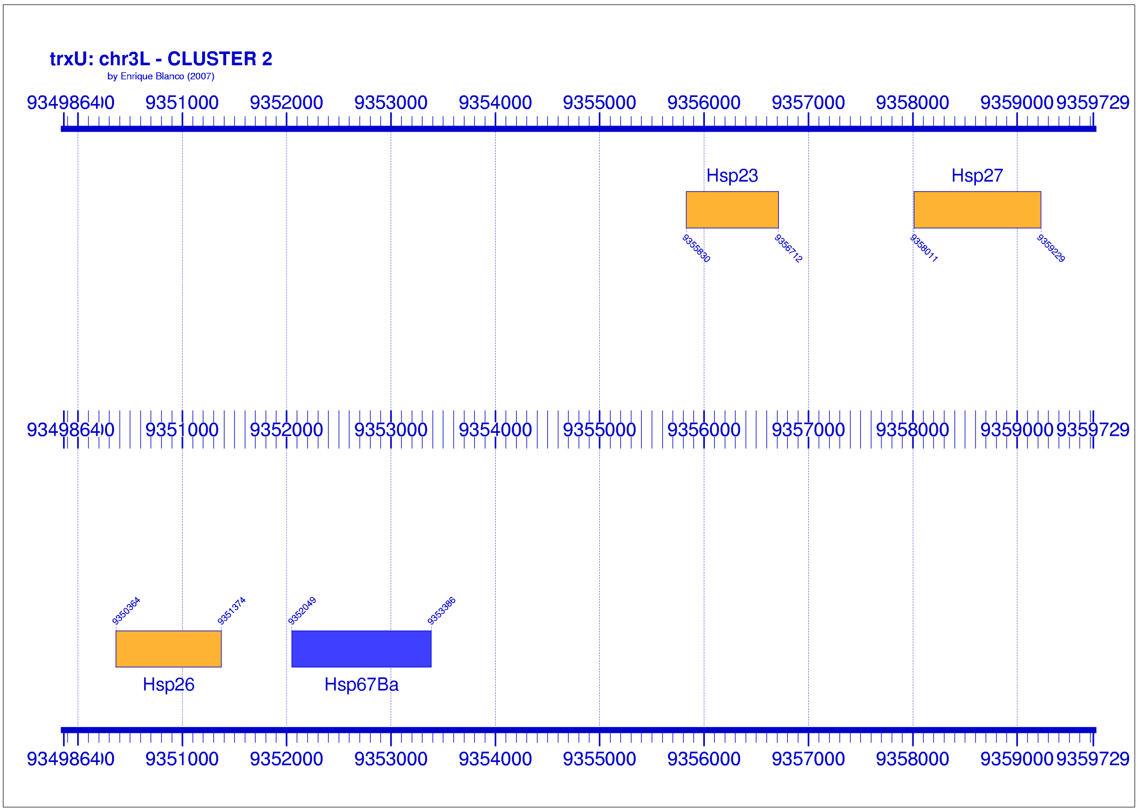

Enrique Blanco © 2007 — May 22, 2007

trxU – chr3L: 11482913 - 11487349

Genomic components: 3 coregulated genes, 4 genes

Chr	Strand	Start	End	RefSeq	Name	Exons	Description
chr3L	-	11482913	11483341	NM_057371	Sgs8	2	Salivary gland secretion 8 CG6132-PA
chr3L	+	11483807	11484194	NM_057370	Sgs7	2	Salivary gland secretion 7 CG18087-PA
chr3L	-	11484269	11484536	NM_206323	CG33272	1	CG33272-PA
chr3L	+	11486167	11487349	NM_079300	Sgs3	2	Salivary gland secretion 3 CG11720-PA

Cluster size: 4437 nucleotides

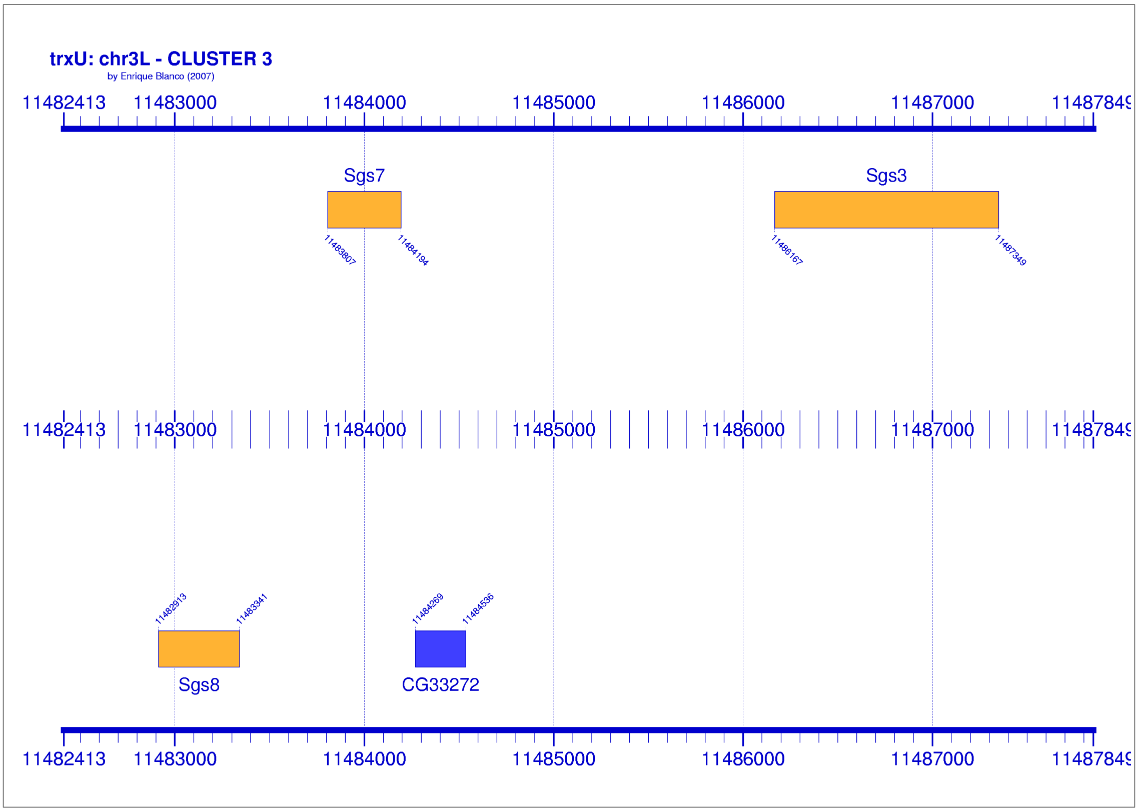

Enrique Blanco © 2007 — May 22, 2007

trxU – chr3L: 11917595 - 11926172

Genomic components: 3 coregulated genes, 3 genes

CHR	Strand	Start	End	RefSeq	Name	Exons	Description
CHR3L	-	11917595	11918793	NM_140269	CG5883	2	CG5883-PA
CHR3L	+	11921632	11923195	NM_140270	CG7252	2	CG7252-PA
CHR3L	+	11923787	11926172	NM_140271	CG17826	2	CG17826-PA

Cluster size: 8578 nucleotides

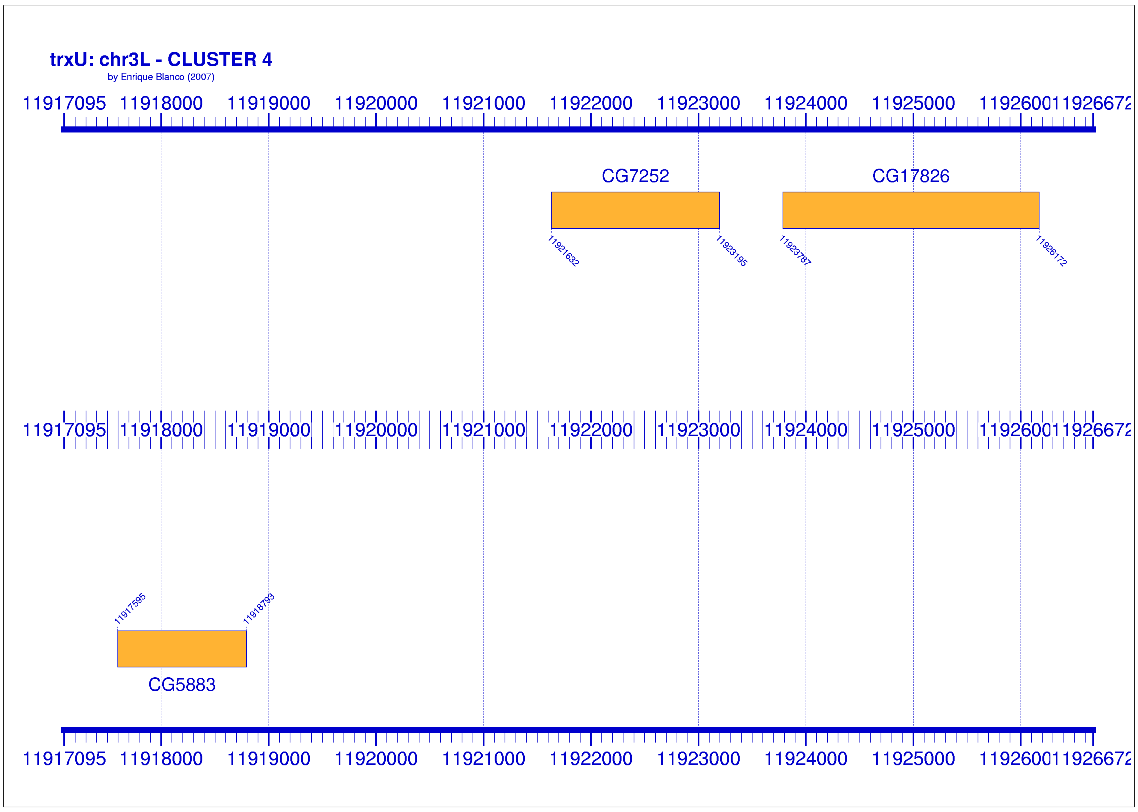

Enrique Blanco © 2007 — May 22, 2007

trxU – chr3L: 15017322 - 15026980

Genomic components: 3 coregulated genes, 4 genes

CHR	Strand	Start	End	RefSeq	Name	Exons	Description
CHR3L	-	15017322	15017734	NM_140481	CG13461	1	CG13461-PA
CHR3L	-	15018826	15019392	NM_140482	CG18649	1	CG18649-PA
CHR3L	+	15020029	15020824	NM_140483	CG13463	1	CG13463-PA
CHR3L	-	15026328	15026980	NM_140484	CG13460	1	CG13460-PA

Cluster size: 9659 nucleotides

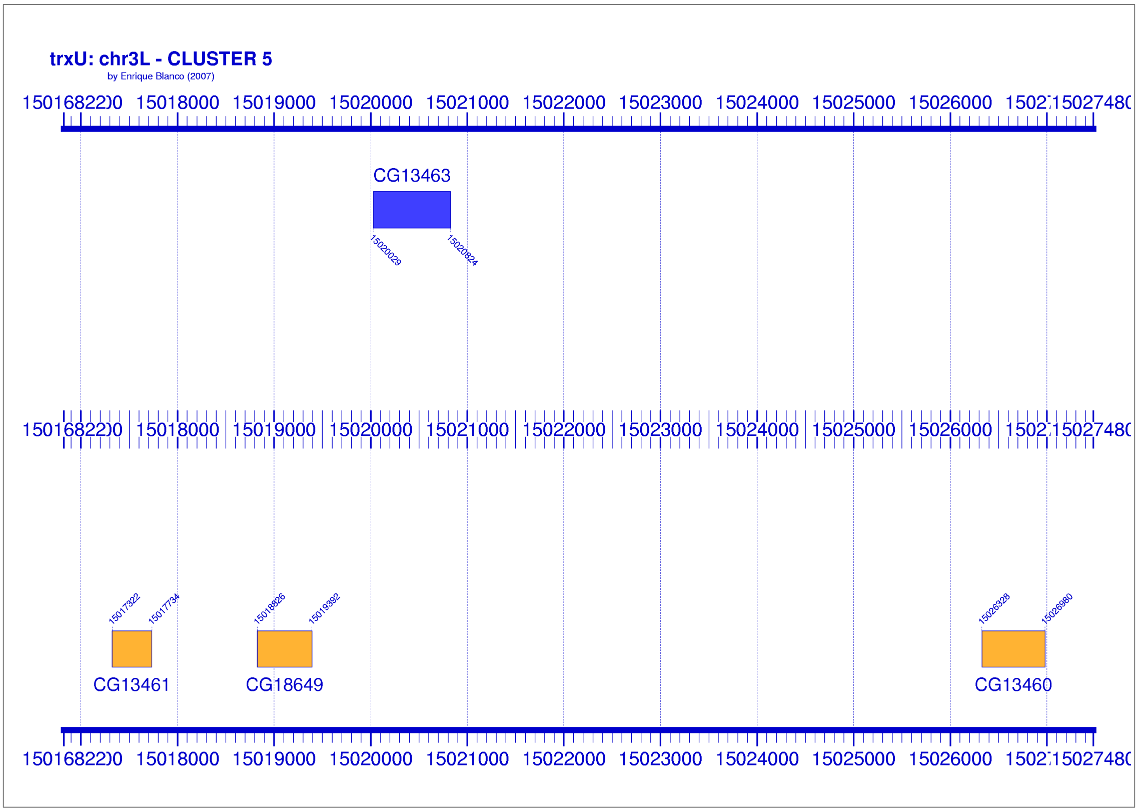

Enrique Blanco © 2007 — May 22, 2007

trxU – chr3L: 20138463 - 20154238

Genomic components: 3 coregulated genes, 5 genes

CHR	Strand	Start	End	RefSeq	Name	Exons	Description
CHR3L	-	20138463	20139884	NM_140931	CG7290	2	CG7290-PA
CHR3L	+	20141740	20142798	NM_140932	CG6996	1	CG6996-PA
CHR3L	-	20141782	20143320	NM_168832	CG32224	2	CG32224-PA
CHR3L	-	20144276	20145959	NM_140933	CG7017	2	CG7017-PA
CHR3L	-	20152342	20154238	NM_140934	CG6933	2	CG6933-PA, isoform A

Cluster size: 15776 nucleotides

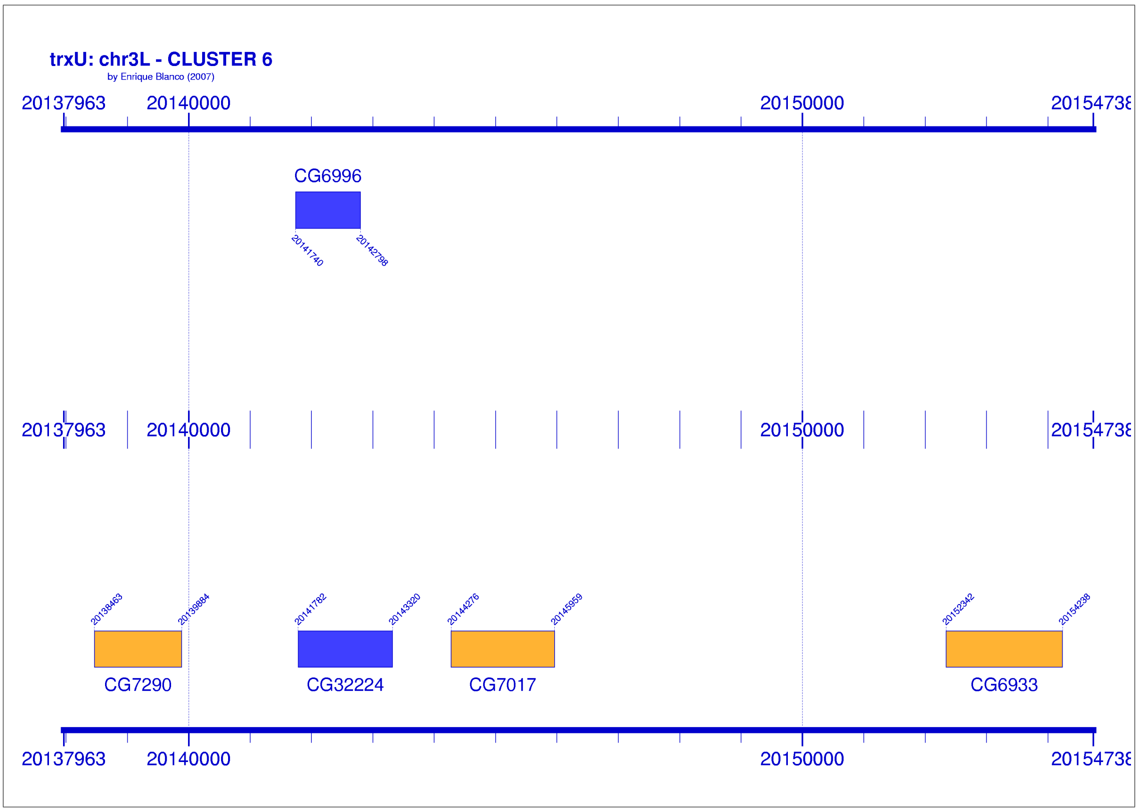

Enrique Blanco © 2007 — May 22, 2007

trxU – chr2R: 6757647 - 6771890

Genomic components: 4 coregulated genes, 7 genes

CHR	Strand	Start	End	RefSeq	Name	Exons	Description
CHR2R	-	6757647	6758102	NM_136806	CG9080	1	CG9080-PA
CHR2R	+	6760196	6760910	NM_136807	CG13226	1	CG13226-PA
CHR2R	+	6762023	6762561	NM_165812	CG30029	1	CG30029-PA
CHR2R	+	6764032	6764517	NM_136809	CG7738	1	CG7738-PA
CHR2R	+	6766274	6767820	NM_078965	Or47a	4	Odorant receptor 47a CG13225-PA
CHR2R	-	6768035	6768798	NM_136810	CG9079	3	CG9079-PA
CHR2R	+	6771075	6771890	NM_136811	CG13224	1	CG13224-PA

Cluster size: 14244 nucleotides

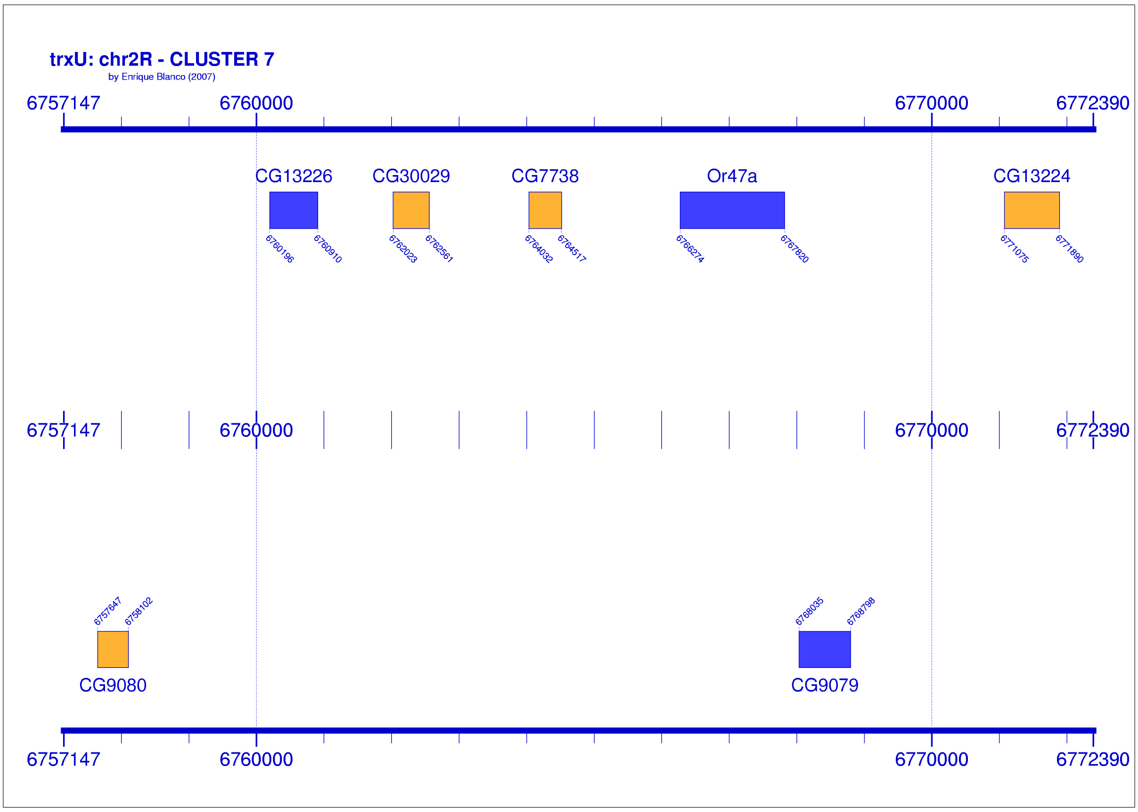

Enrique Blanco © 2007 — May 22, 2007

trxU – chr2R: 13899427 - 13905472

Genomic components: 3 coregulated genes, 7 genes

CHR	Strand	Start	End	RefSeq	Name	Exons	Description
CHR2R	+	13899427	13899778	NM_145336	CG18107	2	CG18107-PA
CHR2R	-	13899774	13900451	NM_137476	CG15067	2	CG15067-PA
CHR2R	+	13901459	13901898	NM_166277	IM2	2	Immune induced molecule 2 CG18106-PA
CHR2R	+	13902982	13903370	NM_144111	IM3	2	Immune induced molecule 3 CG16844-PA
CHR2R	+	13903957	13904319	NM_144110	CG16836	2	CG16836-PA
CHR2R	+	13904820	13905107	NM_144109	CG15065	2	CG15065-PA
CHR2R	-	13905208	13905472	NM_176222	CG15068	2	CG15068-PA

Cluster size: 6046 nucleotides

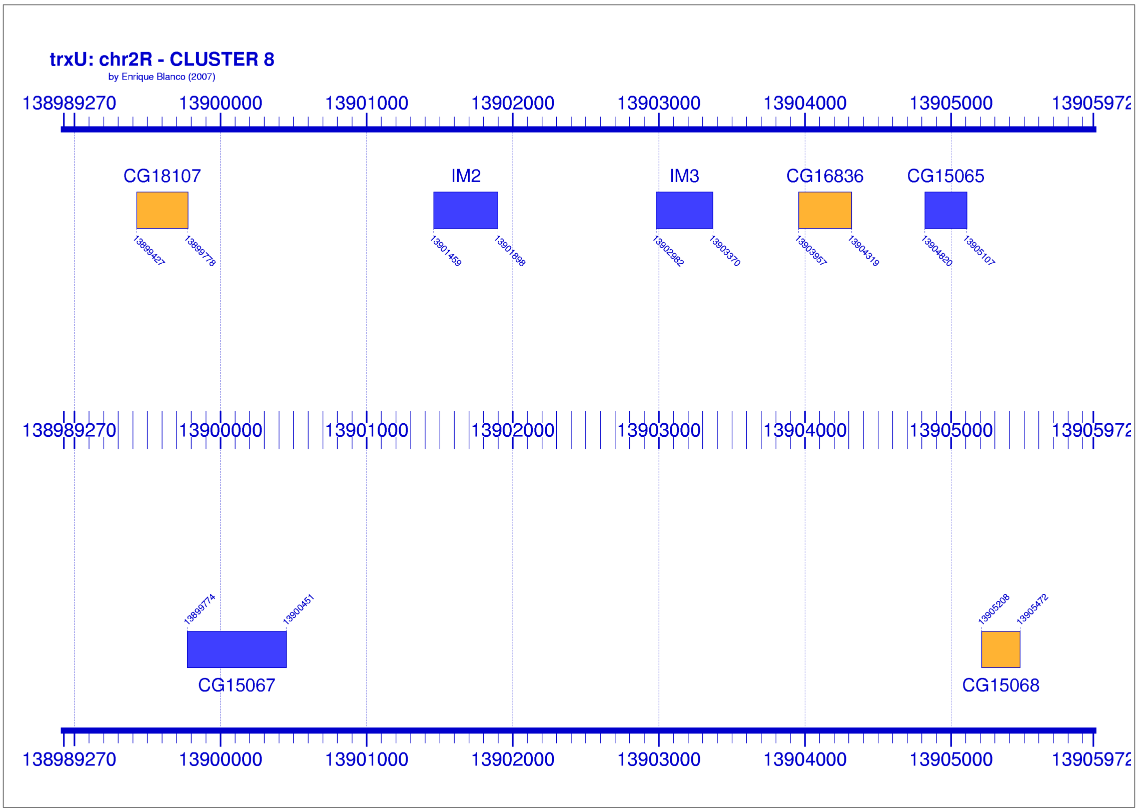

Enrique Blanco © 2007 — May 22, 2007

trxU – chr3R: 10386703 - 10392190

Genomic components: 3 coregulated genes, 4 genes

CHR	Strand	Start	End	RefSeq	Name	Exons	Description
CHR3R	+	10386703	10387178	NM_142114	CG14850	1	CG14850-PA
CHR3R	+	10388083	10388540	NM_142115	CG14851	1	CG14851-PA
CHR3R	-	10390044	10390504	NM_169585	CG8087	1	CG8087-PA
CHR3R	+	10391667	10392190	NM_142116	CG14852	1	CG14852-PA

Cluster size: 5488 nucleotides

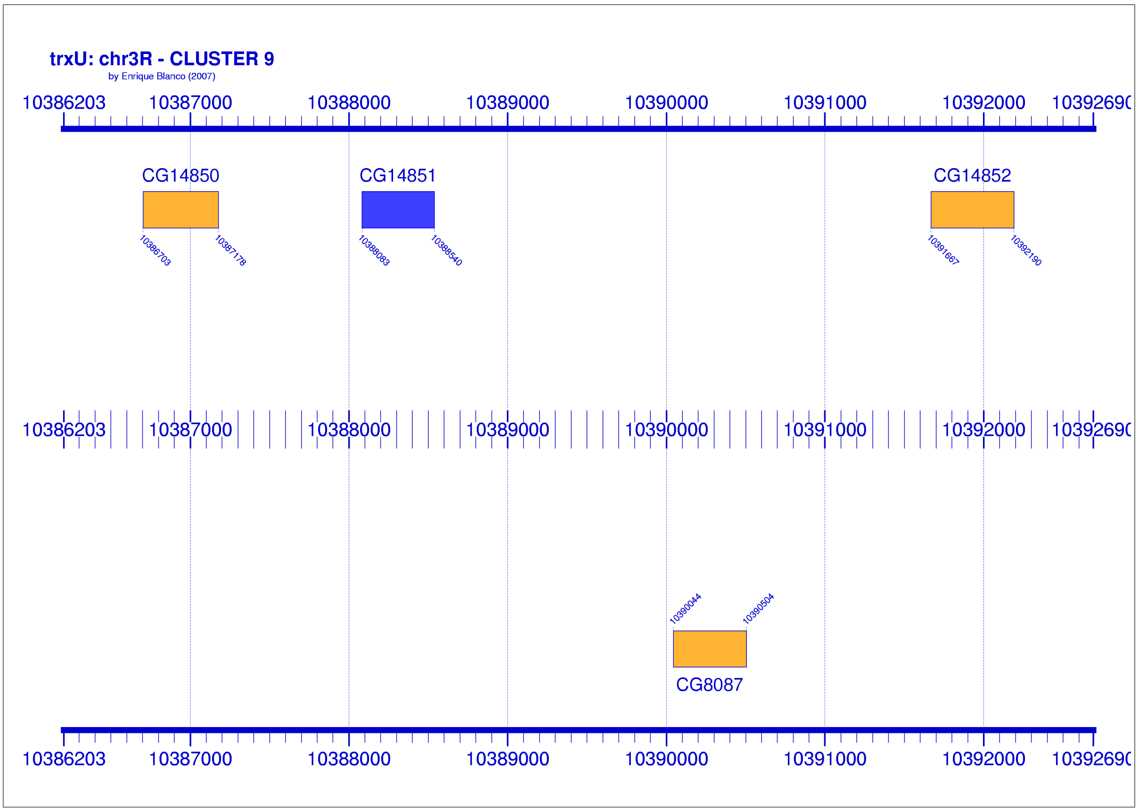

Enrique Blanco © 2007 — May 22, 2007

trxU – chr3R: 14551120 - 14558756

Genomic components: 3 coregulated genes, 3 genes

Chr	Strand	Start	End	RefSeq	Name	Exons	Description
chr3R	-	14551120	14552156	NM_142490	CG7714	2	CG7714-PA
chr3R	-	14553294	14554190	NM_142491	CG7715	2	CG7715-PA
chr3R	+	14558422	14558756	NM_142492	CG14302	2	CG14302-PA

Cluster size: 7637 nucleotides

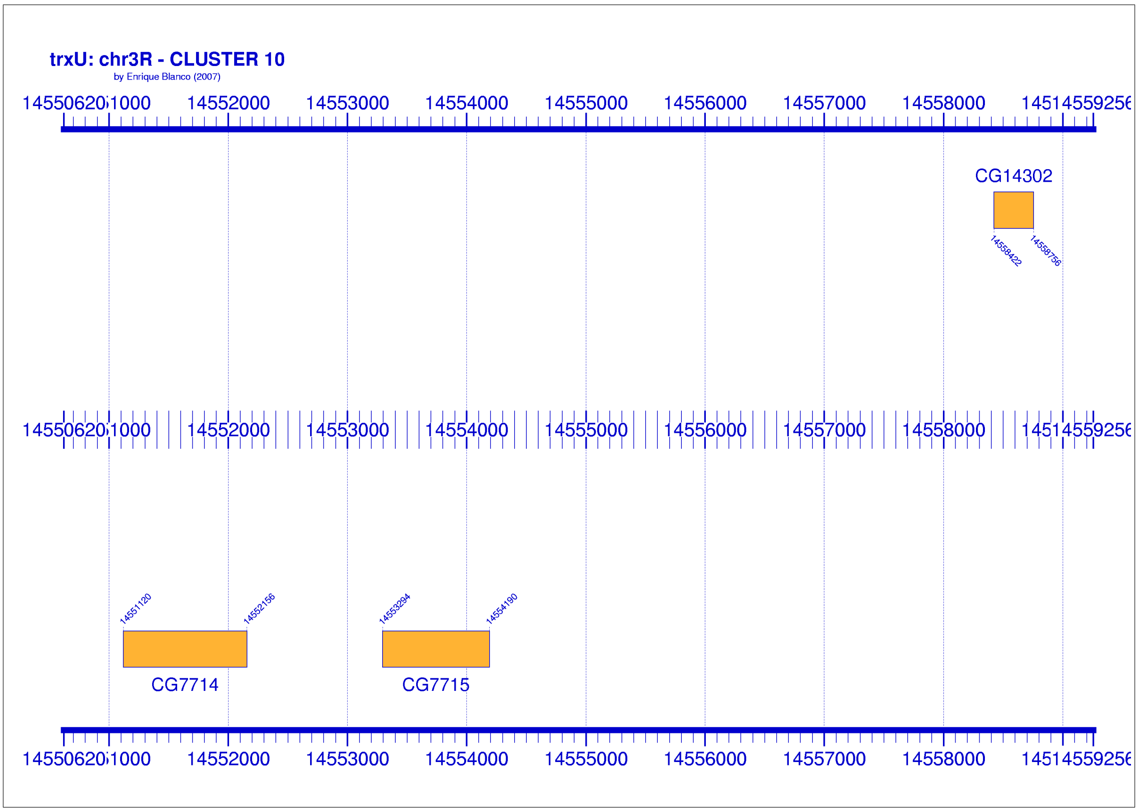

trxD – chr2L: 7740552 - 7753160

Genomic components: 3 coregulated genes, 4 genes

CHR	Strand	Start	End	RefSeq	Name	Exons	Description
CHR2L	-	7740552	7741542	NM_057767	Acp1	2	Adult cuticle protein 1 CG7216-PA
CHR2L	-	7743676	7744841	NM_135297	CG7214	2	CG7214-PA
CHR2L	+	7747342	7747874	NM_135298	CG7211	2	CG7211-PA
CHR2L	-	7752167	7753160	NM_135299	CG7203	2	CG7203-PA

Cluster size: 12609 nucleotides

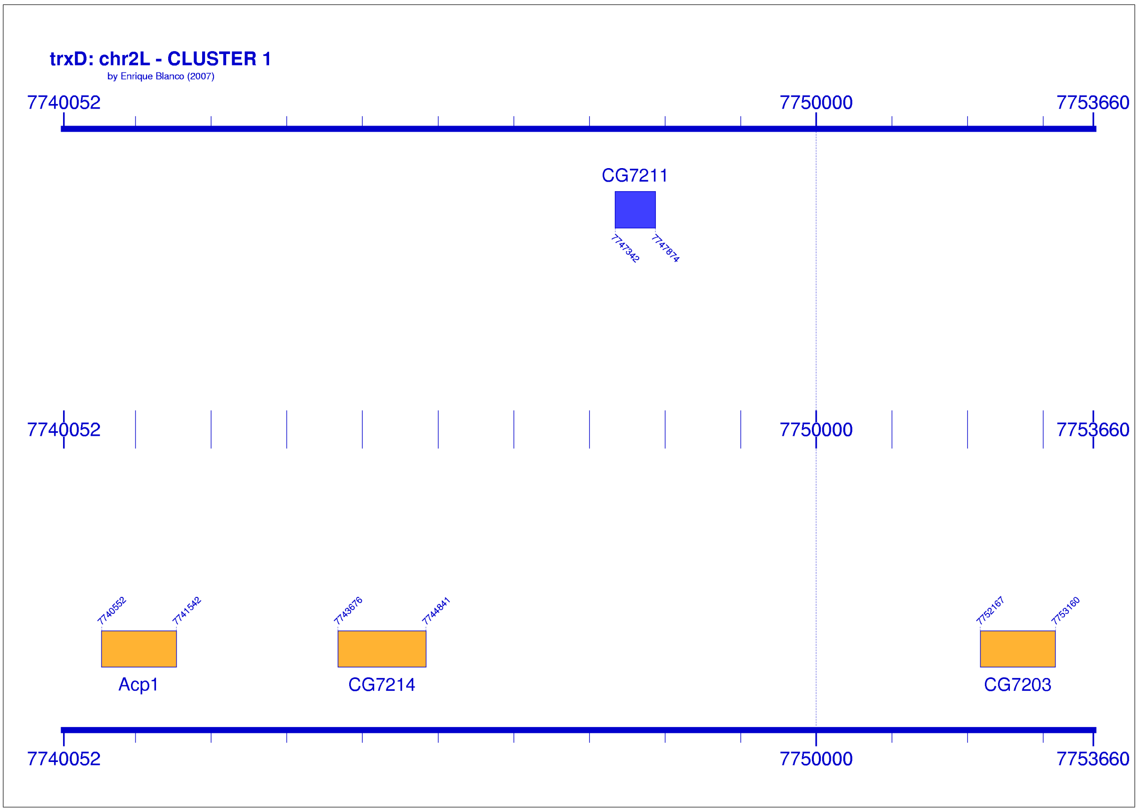

Enrique Blanco © 2007 — May 22, 2007

trxD – chr3L: 1286480 - 1296909

Genomic components: 3 coregulated genes, 4 genes

CHR	Strand	Start	End	RefSeq	Name	Exons	Description
CHR3L	+	1286480	1288253	NM_138250	CG9149	6	CG9149-PA
CHR3L	-	1288808	1290949	NM_138251	CG2277	1	CG2277-PA
CHR3L	+	1290755	1295343	NM_176271	CG2469	7	CG2469-PB, isoform B
CHR3L	-	1295250	1296909	NM_138253	CG9186	3	CG9186-PB, isoform B

Cluster size: 10430 nucleotides

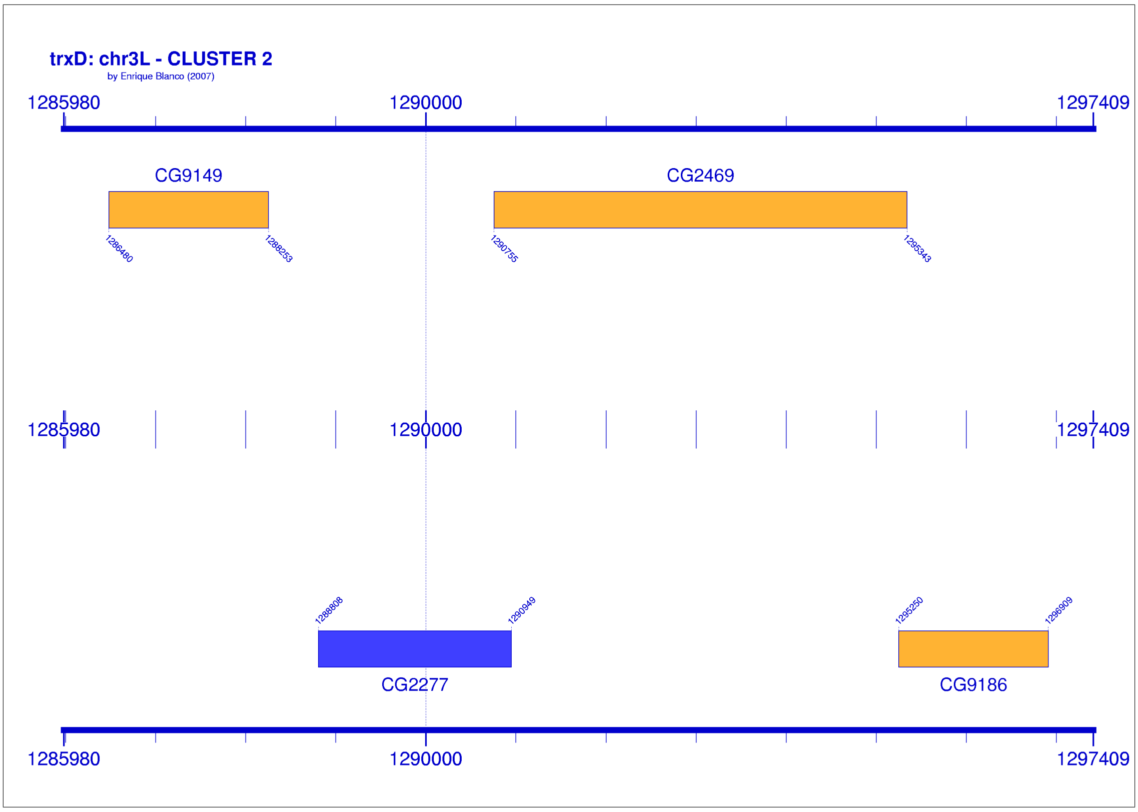

Enrique Blanco © 2007 — May 22, 2007

trxD – chr3L: 4429174 - 4447704

Genomic components: 3 coregulated genes, 6 genes

CHR	Strand	Start	End	RefSeq	Name	Exons	Description
CHR3L	+	4429174	4430664	NM_139646	CG12607	3	CG12607-PB
CHR3L	-	4438168	4438983	NM_139647	CG11345	2	CG11345-PA
CHR3L	-	4440039	4441160	NM_139648	CG15022	2	CG15022-PA
CHR3L	-	4441593	4442099	NM_139649	CG15023	1	CG15023-PA
CHR3L	-	4443894	4444185	NM_144079	CG15024	1	CG15024-PA
CHR3L	-	4446072	4447704	NM_168087	CG32241	2	CG32241-PA

Cluster size: 18531 nucleotides

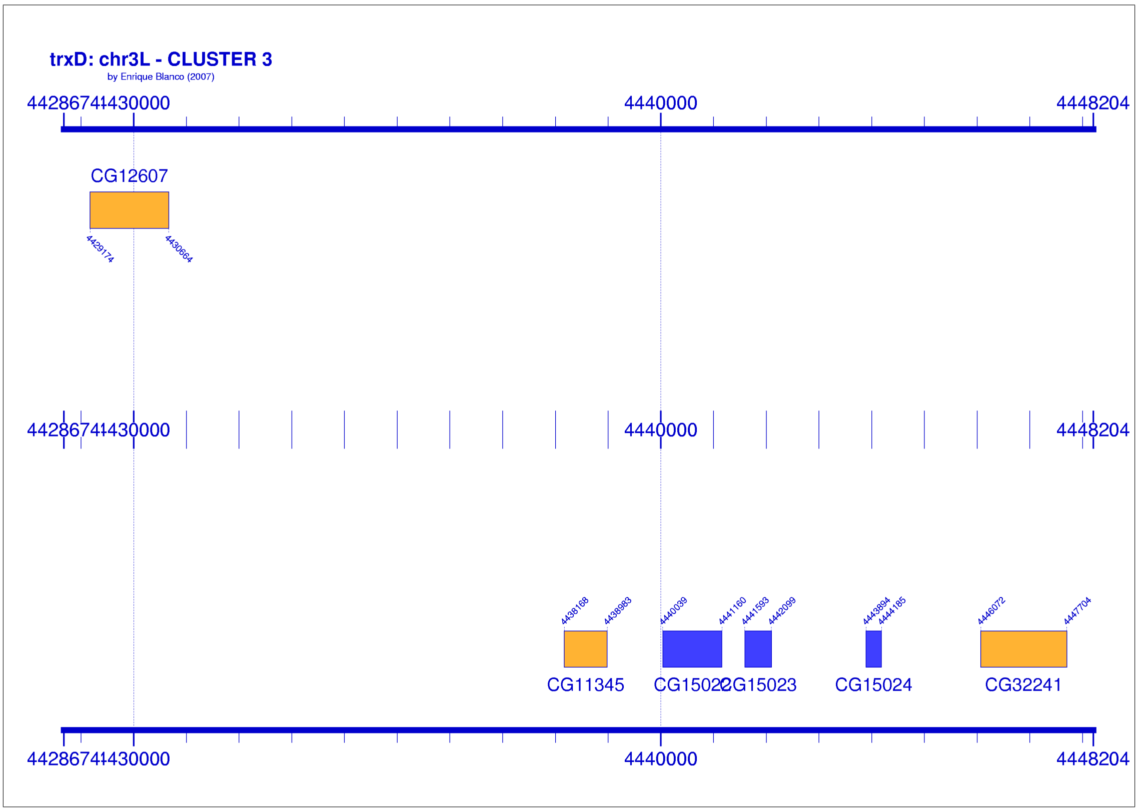

Enrique Blanco © 2007 — May 22, 2007

trxD – chr3L: 6097832 - 6125586

Genomic components: 10 coregulated genes, 15 genes

CHR	Strand	Start	End	RefSeq	Name	Exons	Description
CHR3L	-	6097832	6102566	NM_001014570	l(3)mbn	7	lethal (3) malignant blood neoplasm CG12755-PC,
CHR3L	-	6104332	6104933	NM_144405	CG18779	2	CG18779-PA
CHR3L	-	6105259	6106056	NM_144404	CG18778	2	CG18778-PA
CHR3L	-	6106936	6107547	NM_057924	Lcp65Ag2	2	Lcp65Ag2 CG10534-PA
CHR3L	-	6108643	6109217	NM_057925	Lcp65Ag1	2	Lcp65Ag1 CG10530-PA
CHR3L	-	6110251	6110718	NM_057926	Lcp65Af	2	Lcp65Af CG10533-PA
CHR3L	-	6111667	6112025	NM_176290	Lcp65Ae	2	Lcp65Ae CG10529-PA
CHR3L	-	6113169	6113920	NM_168158	CG32405	2	CG32405-PA
CHR3L	-	6115912	6116330	NM_168159	CG32404	2	CG32404-PA
CHR3L	+	6117353	6117953	NM_057930	Lcp65Ad	2	Lcp65Ad CG6955-PA
CHR3L	+	6118770	6119345	NM_057931	Lcp65Ac	2	Lcp65Ac CG6956-PA
CHR3L	+	6120617	6121036	NM_176291	Lcp65Ab2	1	Lcp65Ab2 CG18773-PA
CHR3L	+	6120668	6120981	NM_080075	Lcp65Ab1	1	Lcp65Ab1 CG32400-PA
CHR3L	-	6121477	6121928	NM_144403	CG18777	2	CG18777-PA
CHR3L	+	6125279	6125586	NM_057932	Lcp65Aa	1	Lcp65Aa CG7287-PA

Cluster size: 27755 nucleotides

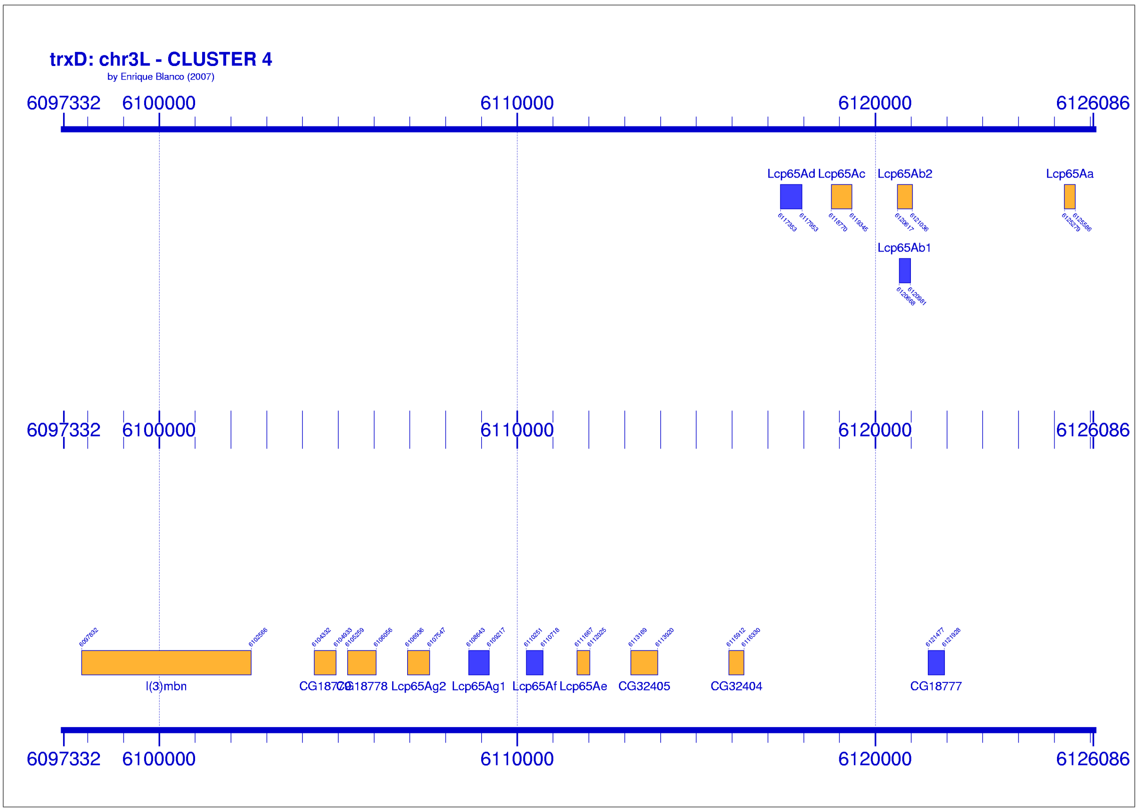

Enrique Blanco © 2007 — May 22, 2007

trxD – chr3L: 8189385 - 8196898

Genomic components: 3 coregulated genes, 3 genes

CHR	Strand	Start	End	RefSeq	Name	Exons	Description
CHR3L	+	8189385	8190145	NM_144191	CG8012	2	CG8012-PA
CHR3L	-	8192387	8192864	NM_139935	CG13674	2	CG13674-PA
CHR3L	+	8196089	8196898	NM_139936	CG13678	2	CG13678-PA

Cluster size: 7514 nucleotides

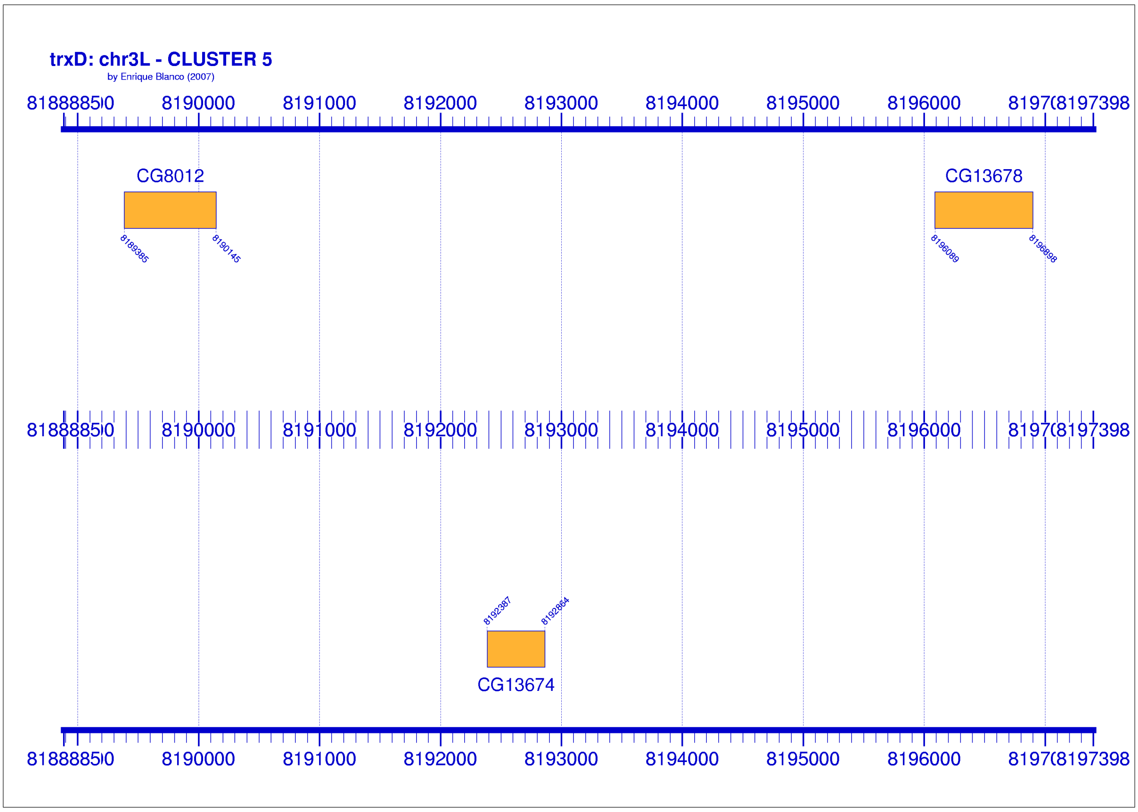

Enrique Blanco © 2007 — May 22, 2007

trxD – chr3L: 11103295 - 11109286

Genomic components: 3 coregulated genes, 4 genes

CHR	Strand	Start	End	RefSeq	Name	Exons	Description
CHR3L	+	11103295	11108621	NM_140184	CG7628	5	CG7628-PA
CHR3L	+	11104781	11105349	NM_080275	nol	1	no optic lobe CG32077-PA
CHR3L	-	11105936	11106267	NM_168447	CG32074	1	CG32074-PA
CHR3L	+	11108943	11109286	NM_140185	CG14143	1	CG14143-PA

Cluster size: 5992 nucleotides

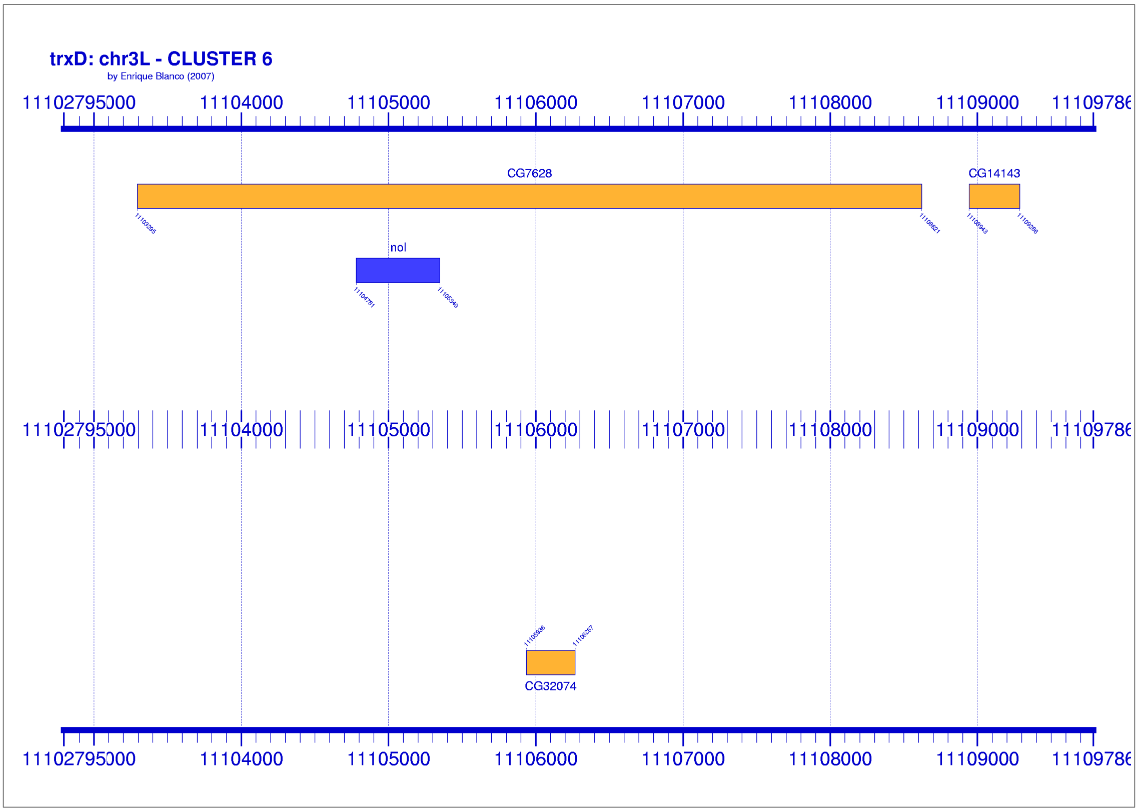

Enrique Blanco © 2007 — May 22, 2007

trxD – chr3L: 16230688 - 16260799

Genomic components: 5 coregulated genes, 14 genes

Chr	Strand	Start	End	RefSeq	Name	Exons	Description
chr3L	+	16230688	16230982	NM_144161	CG13069	1	CG13069-PA
chr3L	-	16231675	16233469	NM_140598	CG4950	1	CG4950-PA
chr3L	+	16234693	16235091	NM_140599	CG13068	2	CG13068-PA
chr3L	+	16235807	16236909	NM_206383	CG13067	3	CG13067-PB, isoform B
chr3L	+	16238418	16238704	NM_144160	CG13066	1	CG13066-PA
chr3L	+	16242691	16243439	NM_140601	CG13065	2	CG13065-PA
chr3L	-	16243916	16244468	NM_140602	CG13050	2	CG13050-PA
chr3L	+	16245737	16246064	NM_206384	CG13064	2	CG13064-PA
chr3L	-	16246389	16247251	NM_140603	CG13049	2	CG13049-PB, isoform B
chr3L	-	16248237	16248916	NM_140604	CG13048	2	CG13048-PA
chr3L	-	16250025	16251401	NM_140605	CG13047	2	CG13047-PA
chr3L	-	16252973	16253474	NM_140606	CG13046	1	CG13046-PA
chr3L	-	16255494	16256056	NM_140607	CG13045	1	CG13045-PA
chr3L	-	16259969	16260799	NM_140608	CG4962	2	CG4962-PA

Cluster size: 30112 nucleotides

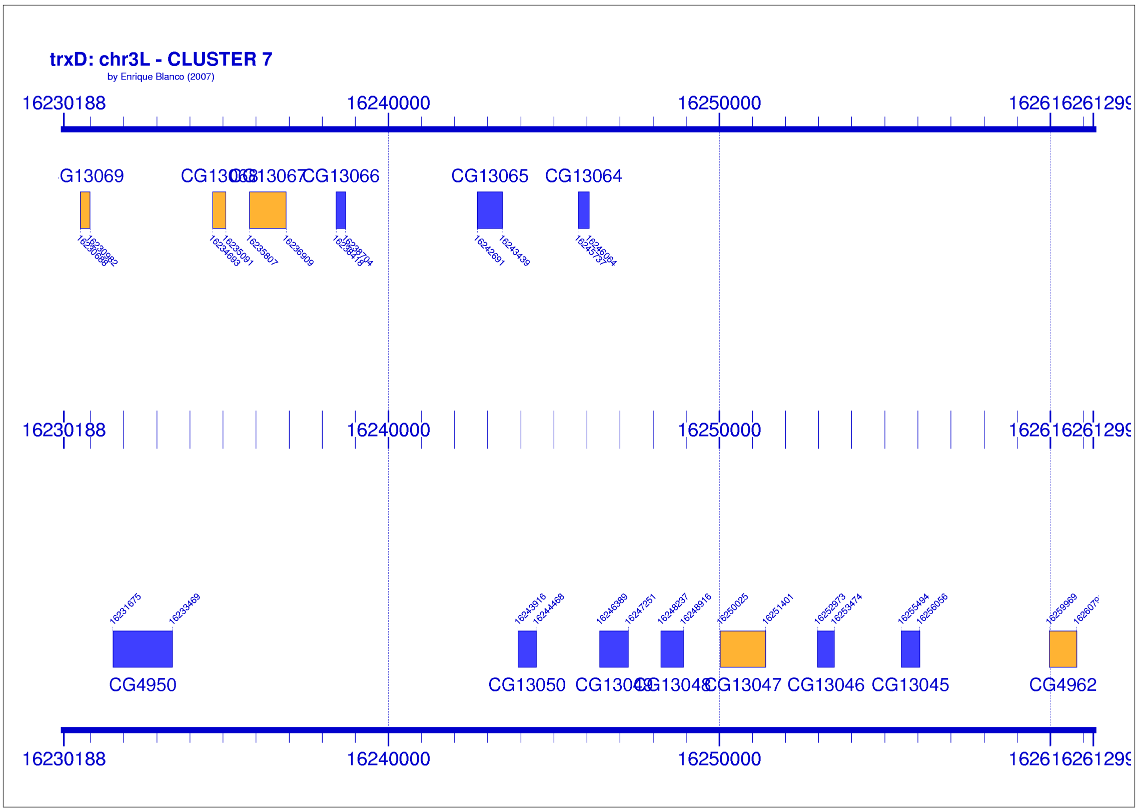

Enrique Blanco © 2007 — May 22, 2007

trxD – chr3L: 16266728 - 16289521

Genomic components: 6 coregulated genes, 11 genes

CHR	Strand	Start	End	RefSeq	Name	Exons	Description
CHR3L	+	16266728	16267286	NM_140609	CG4982	2	CG4982-PA
CHR3L	-	16267749	16268419	NM_140610	CG13044	2	CG13044-PA
CHR3L	-	16270483	16271125	NM_140611	CG13043	1	CG13043-PA
CHR3L	+	16271905	16272355	NM_140612	CG13063	2	CG13063-PA
CHR3L	-	16273258	16273674	NM_140613	CG13042	2	CG13042-PA
CHR3L	+	16274198	16276577	NM_168658	CG32160	2	CG32160-PA
CHR3L	+	16279434	16280204	NM_140614	CG13062	3	CG13062-PA
CHR3L	+	16283283	16283949	NM_144453	Nplp3	2	Neuropeptide-like precursor 3 CG13061-PA
CHR3L	-	16284212	16284779	NM_140615	CG13041	2	CG13041-PA
CHR3L	+	16285119	16285721	NM_140616	CG13060	2	CG13060-PA
CHR3L	+	16288989	16289521	NM_140617	CG13059	2	CG13059-PA

Cluster size: 22794 nucleotides

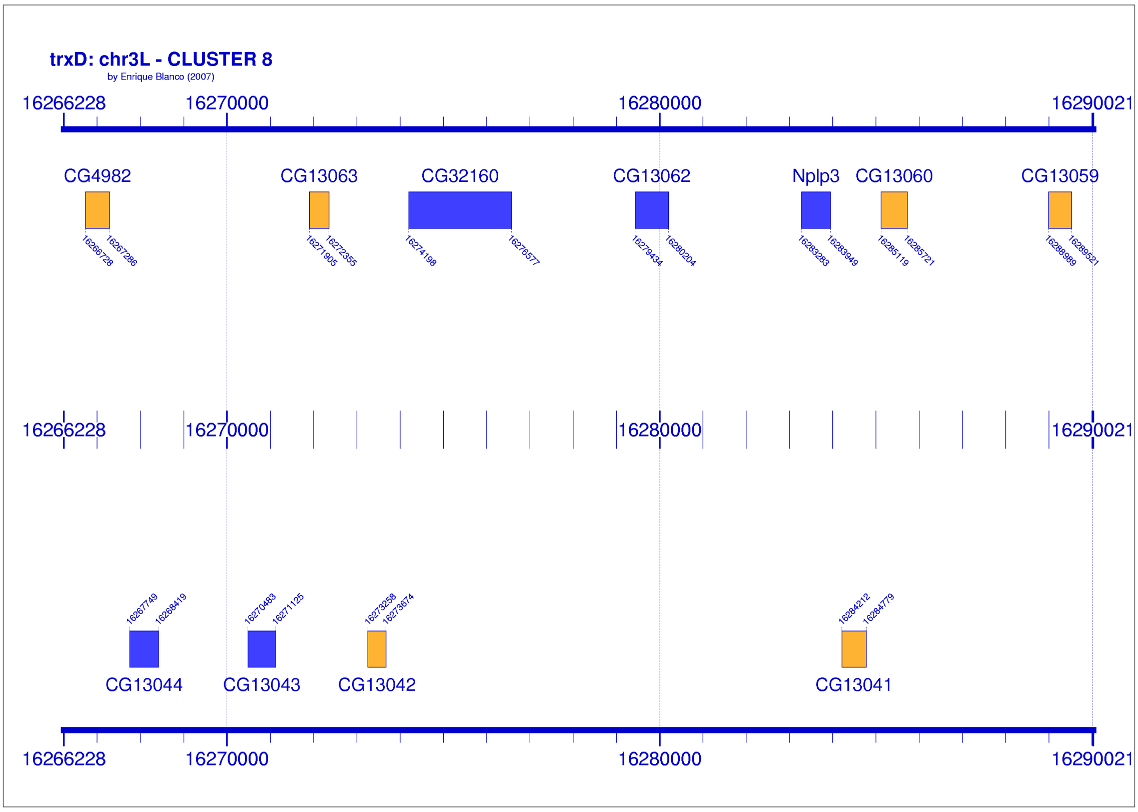

Enrique Blanco © 2007 — May 22, 2007

trxD – chr3L: 21226060 - 21235012

Genomic components: 3 coregulated genes, 4 genes

Chr	Strand	Start	End	RefSeq	Name	Exons	Description
chr3L	-	21226060	21226508	NM_141041	CG11310	2	CG11310-PA
chr3L	+	21229241	21230539	NM_141042	CG7663	2	CG7663-PA
chr3L	-	21230603	21231644	NM_079474	Edg78E	2	Ecdysone-dependent gene 78E CG7673-PA
chr3L	+	21234428	21235012	NM_141043	CG7658	2	CG7658-PA

Cluster size: 8953 nucleotides

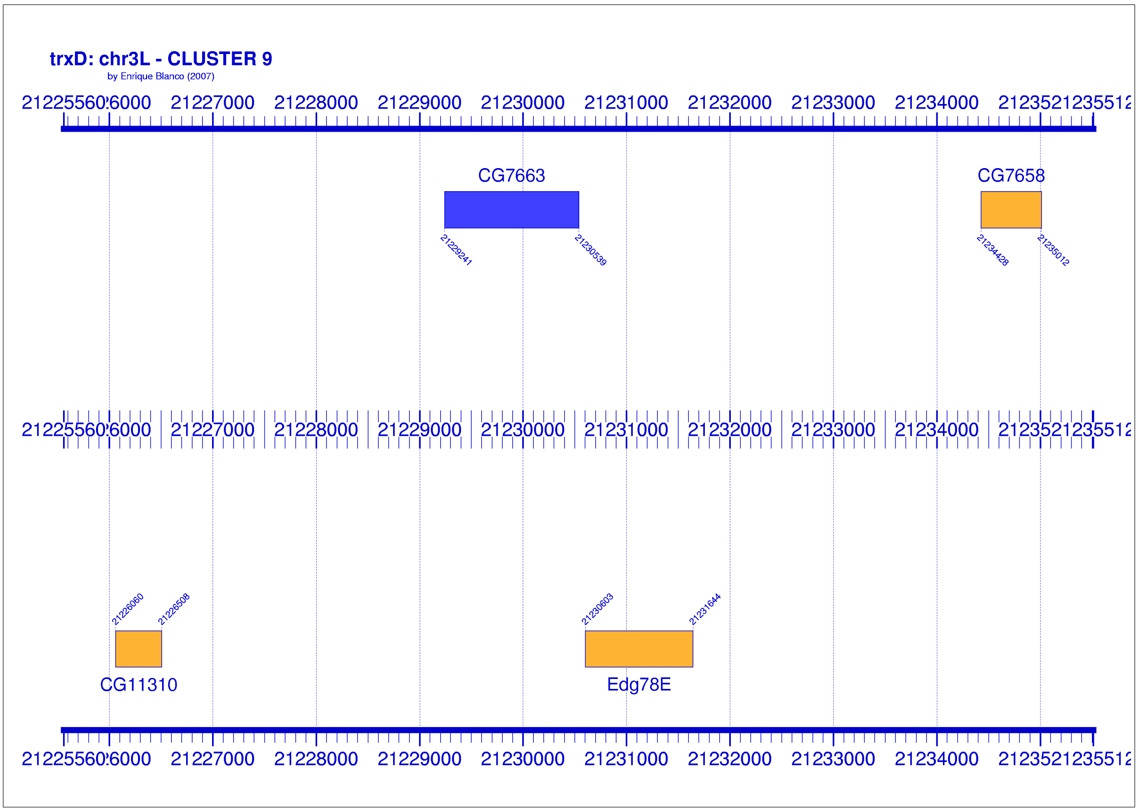

Enrique Blanco © 2007 — May 22, 2007

trxD – chr3L: 21664564 - 21691822

Genomic components: 6 coregulated genes, 9 genes

CHR	Strand	Start	End	RefSeq	Name	Exons	Description
CHR3L	-	21664564	21665388	NM_141097	CG14569	1	CG14569-PA
CHR3L	-	21666650	21667325	NM_141098	CG14568	1	CG14568-PA
CHR3L	+	21667971	21668550	NM_141099	CG14573	1	CG14573-PA
CHR3L	-	21671604	21672175	NM_141100	CG14567	1	CG14567-PA
CHR3L	-	21673606	21674107	NM_141101	CG14566	1	CG14566-PA
CHR3L	+	21675379	21676282	NM_141102	CG14572	1	CG14572-PA
CHR3L	-	21677084	21678105	NM_141103	CG14565	1	CG14565-PA
CHR3L	-	21679199	21682046	NM_168937	Syn1	5	Syntrophin-like 1 CG7152-PA, isoform A
CHR3L	+	21690997	21691822	NM_141104	CG14564	1	CG14564-PA

Cluster size: 27259 nucleotides

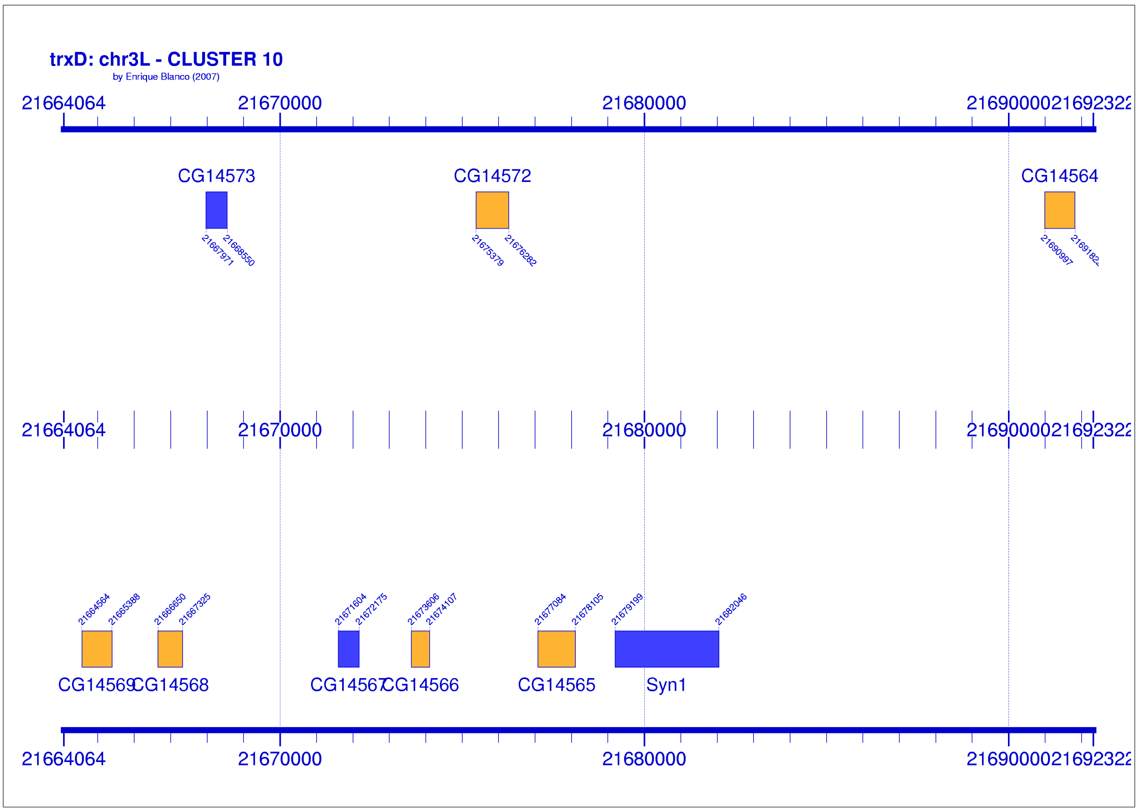

Enrique Blanco © 2007 — May 22, 2007

trxD – chr2R: 7906941 - 7941371

Genomic components: 5 coregulated genes, 13 genes

CHR	Strand	Start	End	RefSeq	Name	Exons	Description
CHR2R	-	7906941	7907561	NM_136929	CG8836	3	CG8836-PA
CHR2R	+	7909389	7910747	NM_078987	Or49a	4	Odorant receptor 49a CG13158-PA
CHR2R	+	7910848	7915011	NM_165895	CG30048	3	CG30048-PA, isoform A
CHR2R	+	7918121	7921019	NM_136930	CG8505	3	CG8505-PA
CHR2R	+	7921911	7922348	NM_136931	CG8510	2	CG8510-PA
CHR2R	+	7923742	7924438	NM_136932	CG8511	2	CG8511-PA
CHR2R	+	7924802	7925559	NM_165897	CG30050	3	CG30050-PA
CHR2R	+	7925985	7926601	NM_001032234	CG33626	3	CG33626-PA
CHR2R	+	7926917	7927583	NM_001032235	CG33627	3	CG33627-PA
CHR2R	+	7929968	7930760	NM_136933	CG8515	2	CG8515-PA
CHR2R	+	7932361	7934047	NM_136934	CG13157	2	CG13157-PA
CHR2R	-	7936554	7938390	NM_136935	CG8834	5	CG8834-PA
CHR2R	+	7938891	7941371	NM_136936	CG8520	5	CG8520-PA

Cluster size: 34431 nucleotides

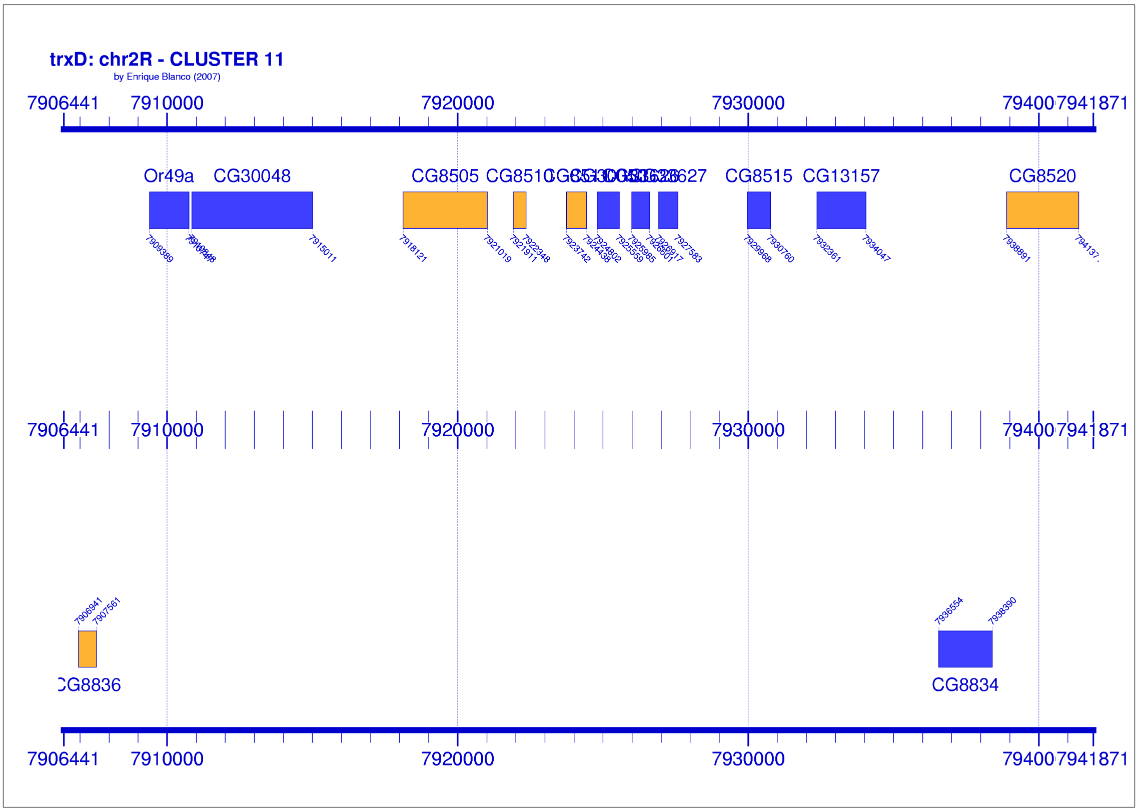

Enrique Blanco © 2007 — May 22, 2007

trxD – chr2R: 12673194 - 12681364

Genomic components: 3 coregulated genes, 3 genes

Chr	Strand	Start	End	RefSeq	Name	Exons	Description
chr2R	-	12673194	12673894	NM_166220	CG30458	2	CG30458-PA
chr2R	-	12675016	12675688	NM_137354	CG30457	2	CG30457-PA
chr2R	-	12680323	12681364	NM_137355	CG10953	2	CG10953-PA

Cluster size: 8171 nucleotides

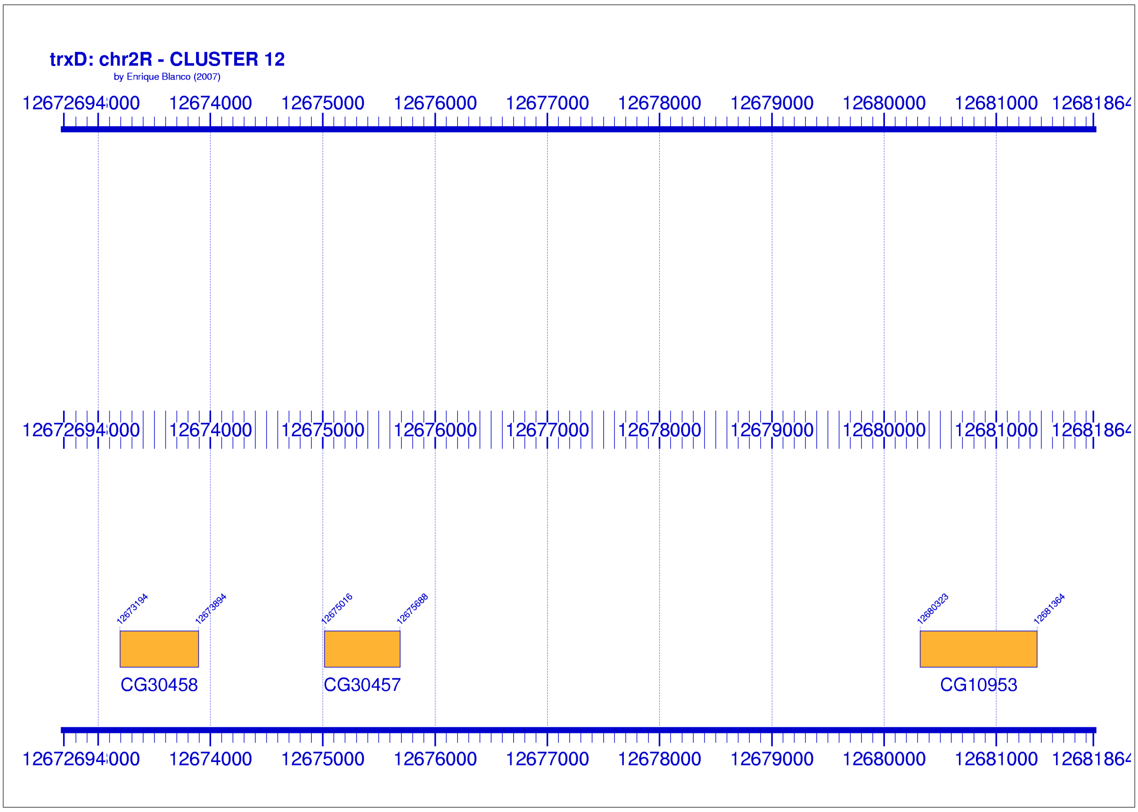

Enrique Blanco © 2007 — May 22, 2007

trxD – chr3R: 2512449 - 2530625

Genomic components: 4 coregulated genes, 7 genes

CHR	Strand	Start	End	RefSeq	Name	Exons	Description
CHR3R	+	2512449	2513761	NM_141420	Ccp84Ag	2	Ccp84Ag CG2342-PA
CHR3R	-	2515394	2515908	NM_141421	Ccp84Af	2	Ccp84Af CG1331-PA
CHR3R	-	2516611	2517480	NM_141422	Ccp84Ae	2	Ccp84Ae CG1330-PA
CHR3R	+	2518519	2519178	NM_141423	Ccp84Ad	2	Ccp84Ad CG2341-PA
CHR3R	-	2521188	2521993	NM_141424	Ccp84Ac	2	Ccp84Ac CG1327-PA
CHR3R	-	2527689	2528599	NM_141425	Ccp84Ab	2	Ccp84Ab CG1252-PA
CHR3R	+	2529803	2530625	NM_141426	Ccp84Aa	2	Ccp84Aa CG2360-PA

Cluster size: 18177 nucleotides

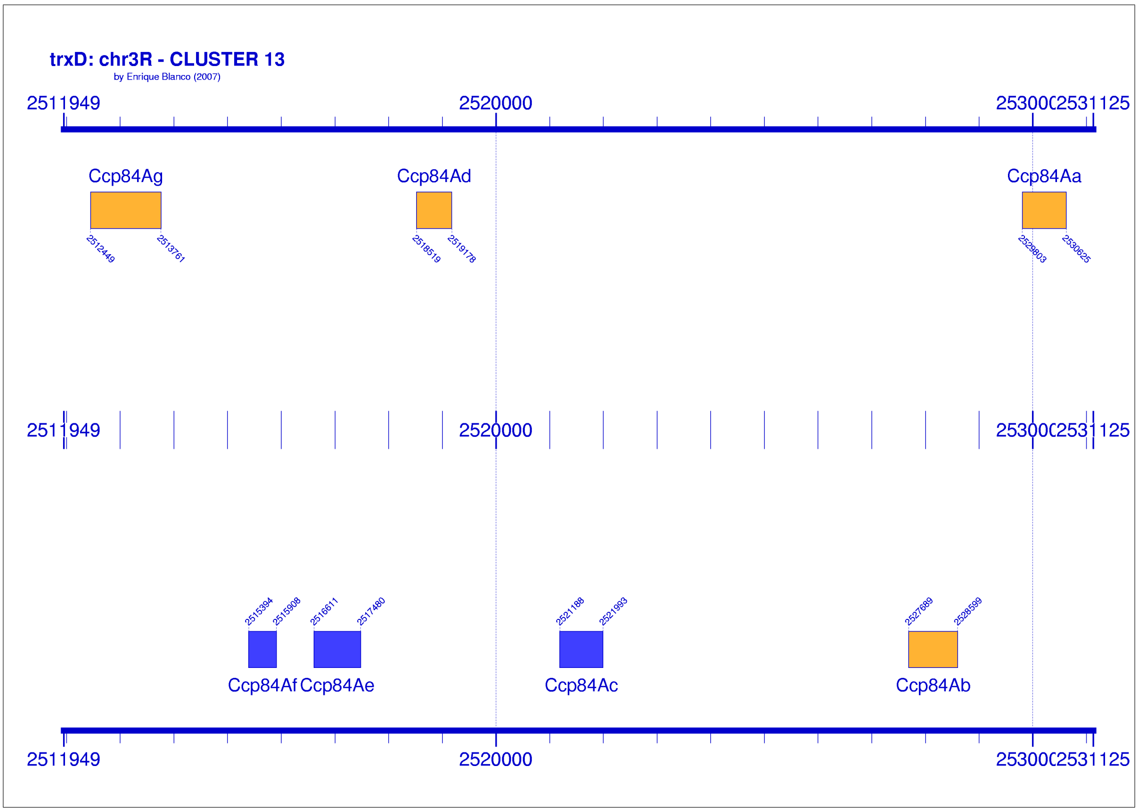

Enrique Blanco © 2007 — May 22, 2007

trxD – chr3R: 22444844 - 22458678

Genomic components: 4 coregulated genes, 8 genes

CHR	Strand	Start	End	RefSeq	Name	Exons	Description
CHR3R	+	22444844	22445952	NM_143227	CG5468	2	CG5468-PA
CHR3R	-	22446329	22447082	NM_143228	CG14240	1	CG14240-PA
CHR3R	-	22448172	22449270	NM_143229	CG6478	2	CG6478-PA
CHR3R	-	22450301	22451374	NM_170279	CG6447	2	CG6447-PA, isoform A
CHR3R	-	22451731	22452419	NM_143230	CG6452	1	CG6452-PA
CHR3R	-	22453687	22454429	NM_143231	CG6460	1	CG6460-PA
CHR3R	+	22455578	22456500	NM_143232	CG5471	1	CG5471-PA
CHR3R	+	22457594	22458678	NM_170281	CG5476	1	CG5476-PA

Cluster size: 13835 nucleotides

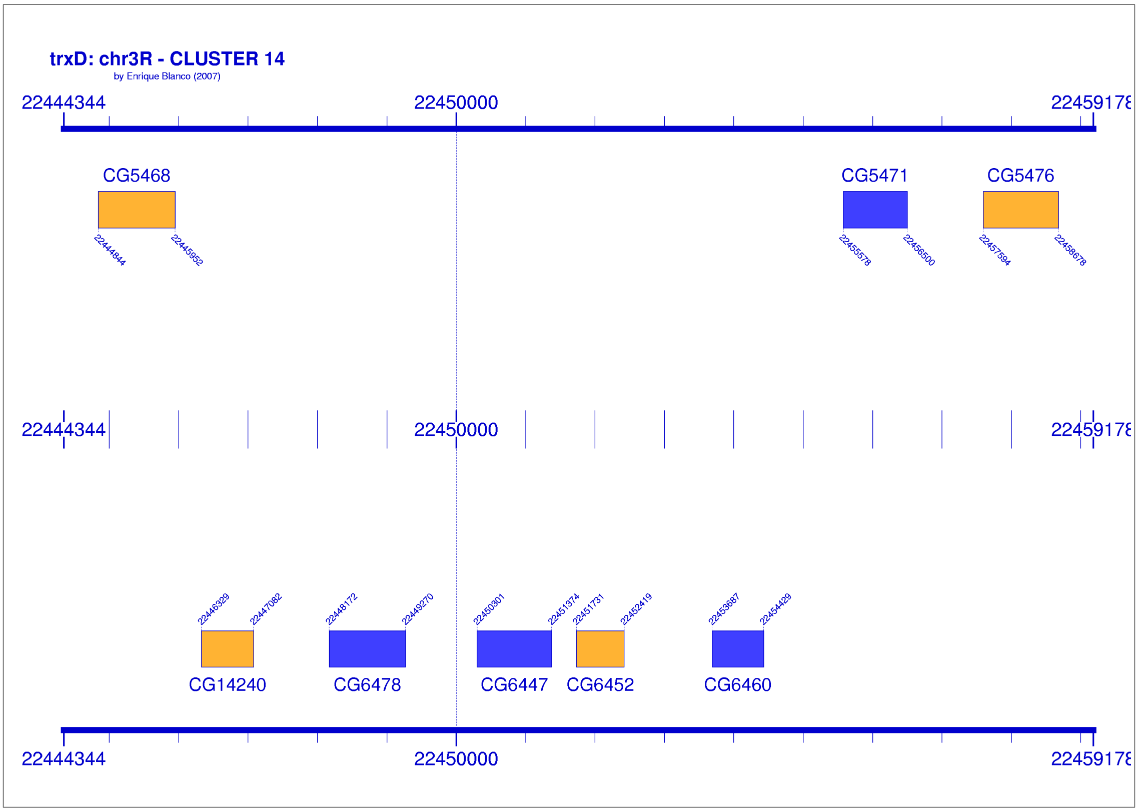

Enrique Blanco © 2007 — May 22, 2007

trxD – chrX: 17040356 - 17065159

Genomic components: 3 coregulated genes, 10 genes

CHR	Strand	Start	End	RefSeq	Name	Exons	Description
CHRX	+	17040356	17041791	NM_167559	CG32564	1	CG32564-PA
CHRX	+	17042372	17042944	NM_167560	CG32563	2	CG32563-PA
CHRX	-	17043378	17044027	NM_132972	CG12995	1	CG12995-PB, isoform B
CHRX	+	17046264	17047844	NM_132973	CG18258	2	CG18258-PA
CHRX	+	17049238	17051151	NM_132974	CG5162	3	CG5162-PA
CHRX	+	17052818	17053431	NM_132975	CG12998	1	CG12998-PA
CHRX	+	17055378	17056467	NM_132976	CG5172	3	CG5172-PA, isoform A
CHRX	+	17057031	17057743	NM_132977	CG12997	1	CG12997-PA
CHRX	-	17059650	17060388	NM_132978	CG10598	1	CG10598-PA, isoform A
CHRX	-	17064272	17065159	NM_132979	CG10597	2	CG10597-PA

Cluster size: 24804 nucleotides

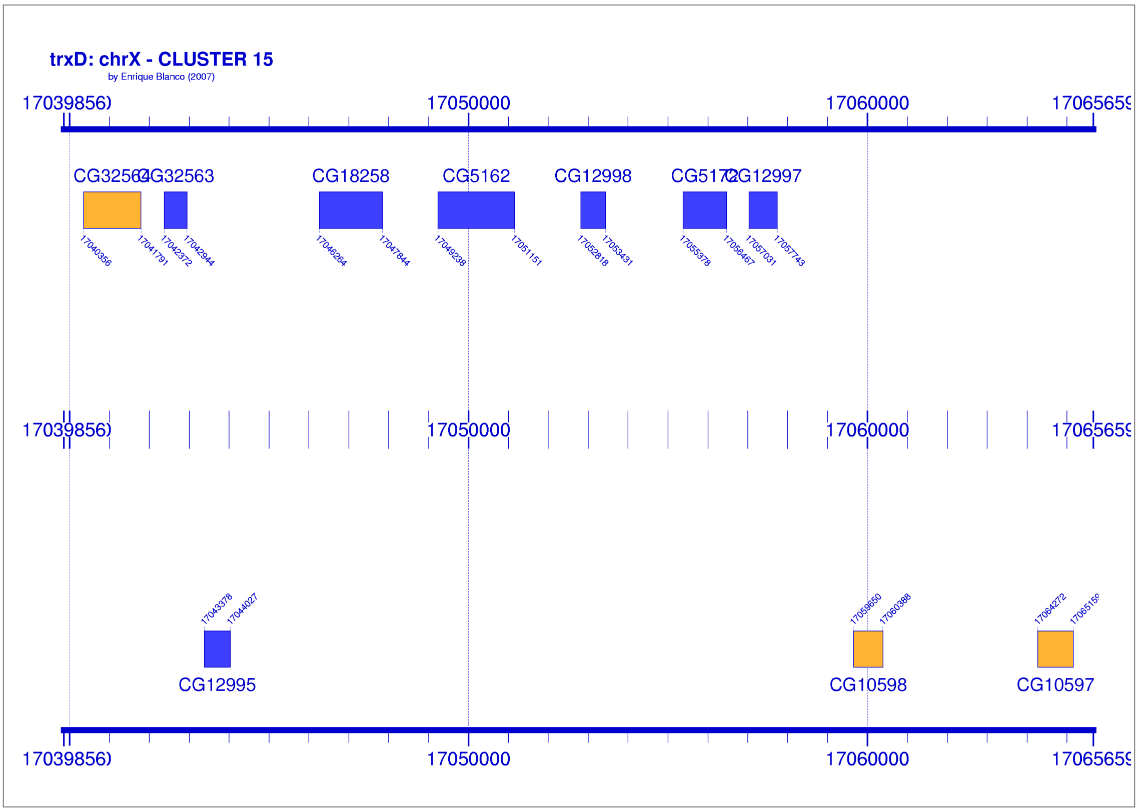

Enrique Blanco © 2007 — May 22, 2007

ash2U – chr2L: 3692842 - 3707060

Genomic components: 3 coregulated genes, 9 genes

CHR	Strand	Start	End	RefSeq	Name	Exons	Description
CHR2L	-	3692842	3693168	NM_134954	CG3513	2	CG3513-PA
CHR2L	-	3693478	3693889	NM_001042865	Acp24A4	2	Acp24A4 CG31779-PB
CHR2L	-	3695027	3695406	NM_134955	CG16713	2	CG16713-PA
CHR2L	-	3696239	3696661	NM_134956	CG16712	2	CG16712-PA
CHR2L	-	3697237	3697777	NM_134957	CG3604	1	CG3604-PA
CHR2L	-	3698554	3699091	NM_134958	CG10031	2	CG10031-PA
CHR2L	+	3703579	3704037	NM_134959	CG2816	3	CG2816-PA
CHR2L	-	3704105	3705462	NM_164549	CG31778	2	CG31778-PA
CHR2L	-	3706544	3707060	NM_164550	CG31777	2	CG31777-PA

Cluster size: 14219 nucleotides

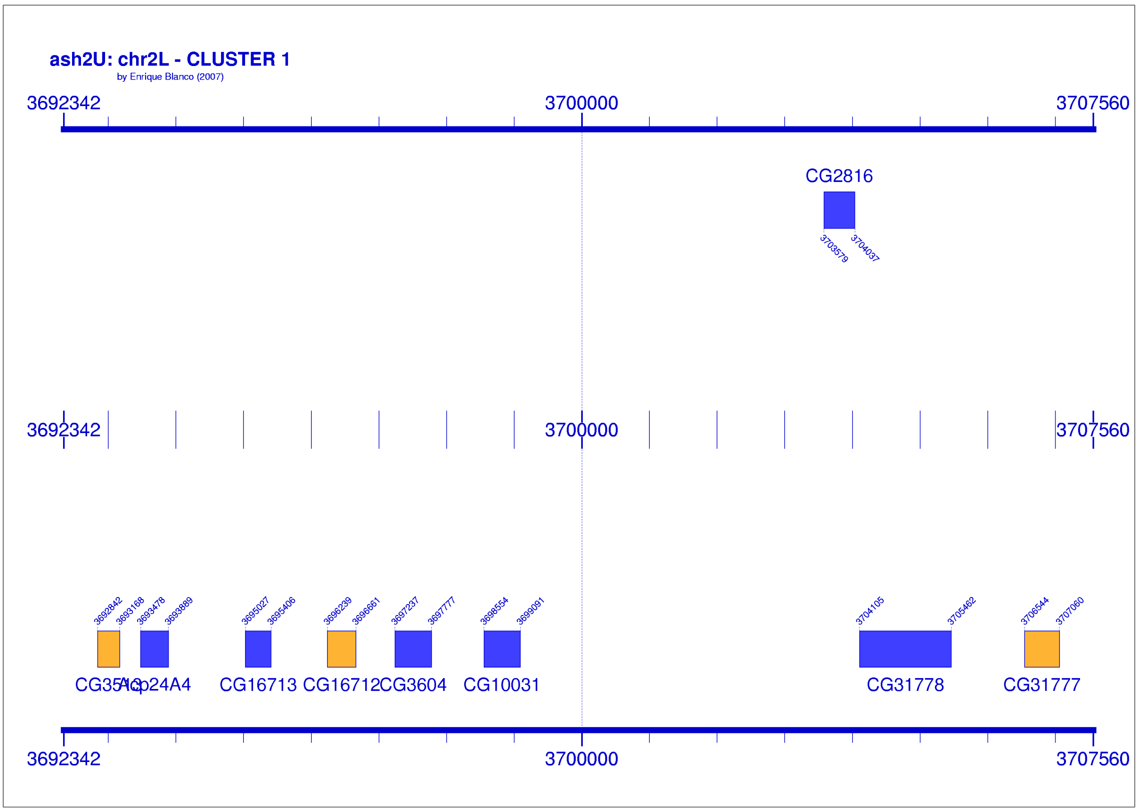

Enrique Blanco © 2007 — May 22, 2007

ash2U – chr3L: 11917595 - 11930731

Genomic components: 4 coregulated genes, 5 genes

CHR	Strand	Start	End	RefSeq	Name	Exons	Description
CHR3L	-	11917595	11918793	NM_140269	CG5883	2	CG5883-PA
CHR3L	+	11921632	11923195	NM_140270	CG7252	2	CG7252-PA
CHR3L	+	11923787	11926172	NM_140271	CG17826	2	CG17826-PA
CHR3L	-	11926347	11927240	NM_140272	CG9781	2	CG9781-PA
CHR3L	+	11928032	11930731	NM_140273	CG7248	2	CG7248-PA

Cluster size: 13137 nucleotides

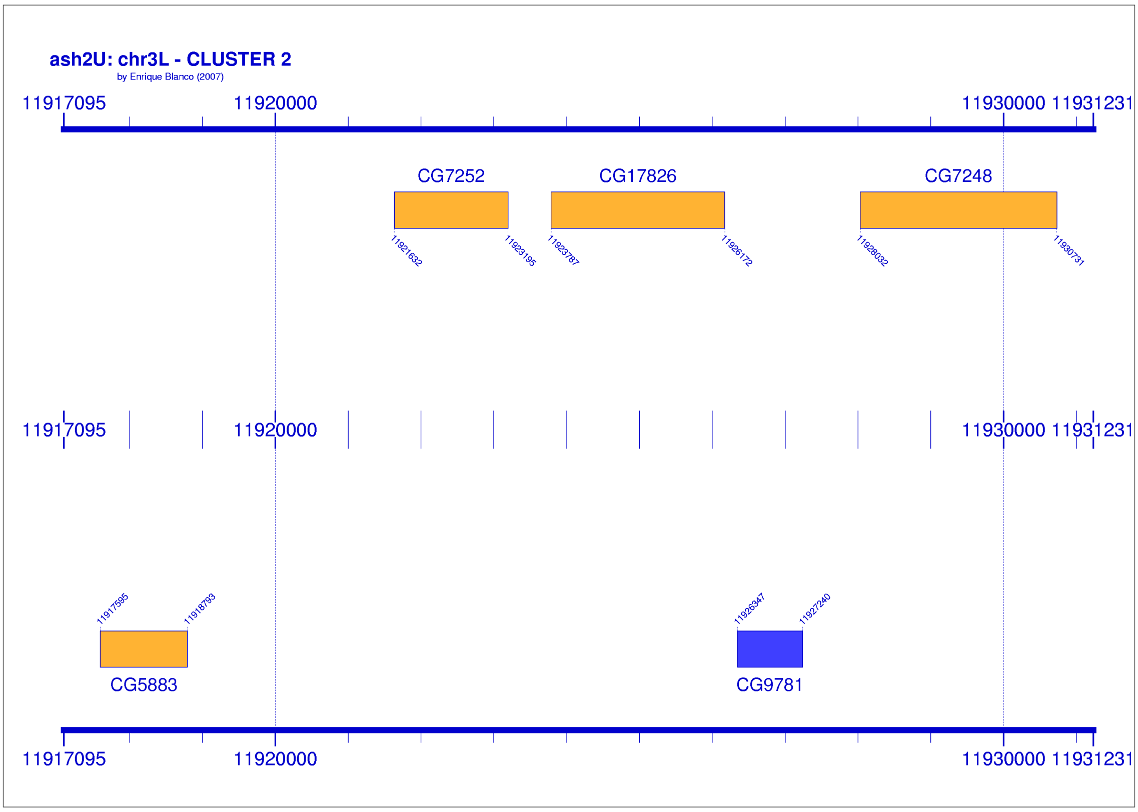

Enrique Blanco © 2007 — May 22, 2007

ash2U – chr3L: 15017322 - 15026980

Genomic components: 3 coregulated genes, 4 genes

CHR	Strand	Start	End	RefSeq	Name	Exons	Description
CHR3L	-	15017322	15017734	NM_140481	CG13461	1	CG13461-PA
CHR3L	-	15018826	15019392	NM_140482	CG18649	1	CG18649-PA
CHR3L	+	15020029	15020824	NM_140483	CG13463	1	CG13463-PA
CHR3L	-	15026328	15026980	NM_140484	CG13460	1	CG13460-PA

Cluster size: 9659 nucleotides

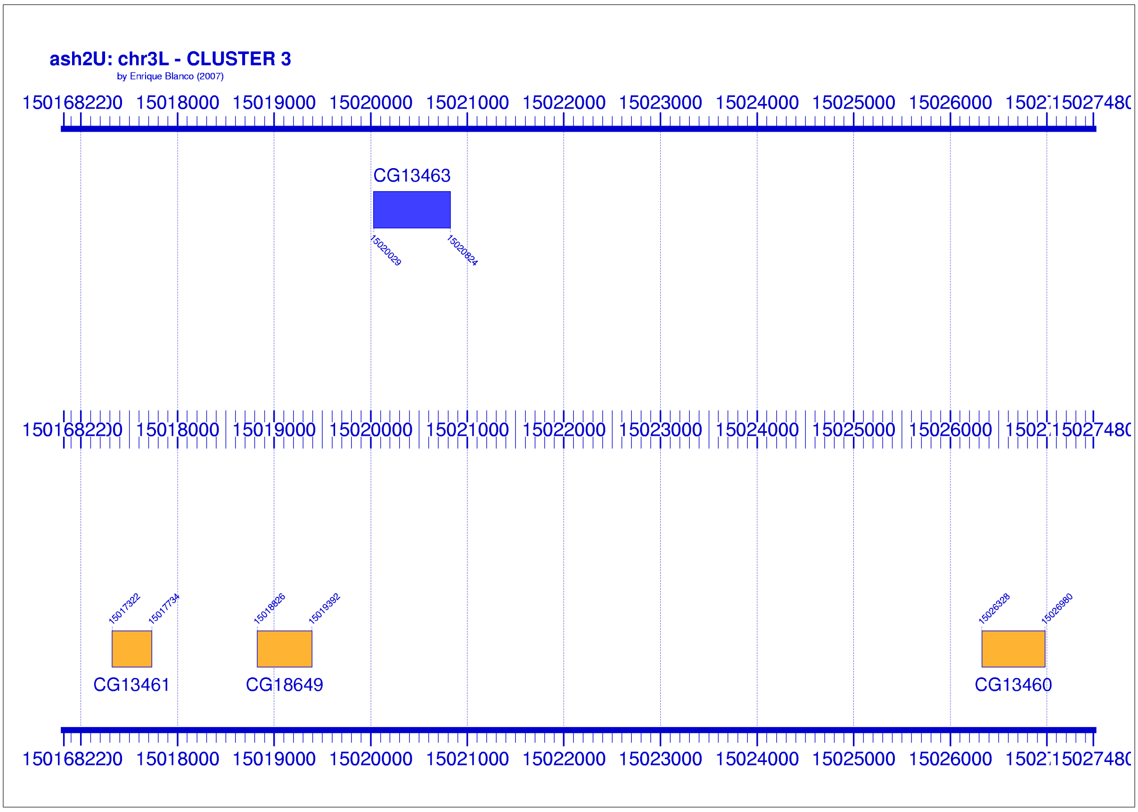

Enrique Blanco © 2007 — May 22, 2007

ash2U – chr2R: 12633571 - 12651715

Genomic components: 3 coregulated genes, 8 genes

CHR	Strand	Start	End	RefSeq	Name	Exons	Description
CHR2R	-	12633571	12635166	NM_080421	Amy-p	1	Amylase proximal CG18730-PA
CHR2R	-	12637757	12639100	NM_001043088	CG10956	2	CG10956-PB
CHR2R	+	12639624	12641243	NM_079044	Amy-d	1	Amylase distal CG17876-PA
CHR2R	-	12641272	12641886	NM_137347	CG15605	1	CG15605-PA
CHR2R	+	12643695	12645105	NM_137348	CG15918	3	CG15918-PA
CHR2R	+	12648385	12648524	NM_001043089	Acp54A1	1	Acp54A1 CG34098-PA
CHR2R	+	12649019	12649708	NM_137349	CG11400	1	CG11400-PA
CHR2R	+	12651360	12651715	NM_137350	CG15917	1	CG15917-PA

Cluster size: 18145 nucleotides

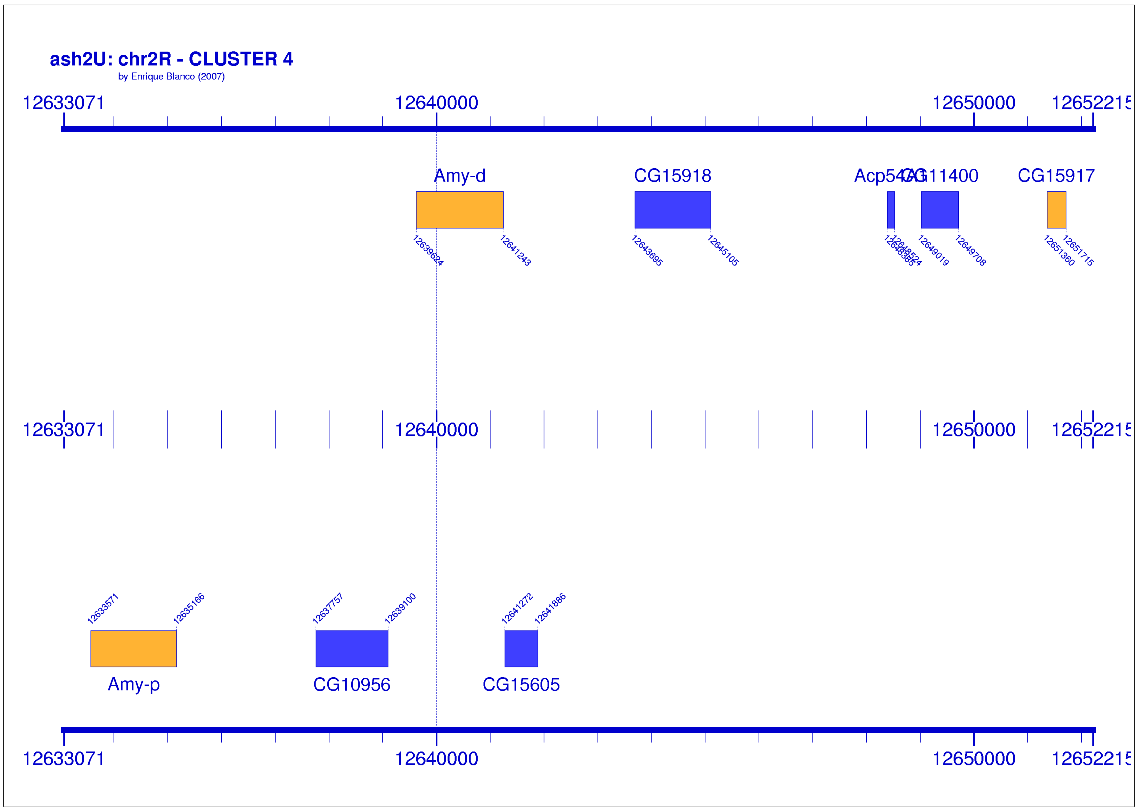

Enrique Blanco © 2007 — May 22, 2007

ash2U – chr2R: 13899427 - 13905472

Genomic components: 4 coregulated genes, 7 genes

Chr	Strand	Start	End	RefSeq	Name	Exons	Description
chr2R	+	13899427	13899778	NM_145336	CG18107	2	CG18107-PA
chr2R	-	13899774	13900451	NM_137476	CG15067	2	CG15067-PA
chr2R	+	13901459	13901898	NM_166277	IM2	2	Immune induced molecule 2 CG18106-PA
chr2R	+	13902982	13903370	NM_144111	IM3	2	Immune induced molecule 3 CG16844-PA
chr2R	+	13903957	13904319	NM_144110	CG16836	2	CG16836-PA
chr2R	+	13904820	13905107	NM_144109	CG15065	2	CG15065-PA
chr2R	-	13905208	13905472	NM_176222	CG15068	2	CG15068-PA

Cluster size: 6046 nucleotides

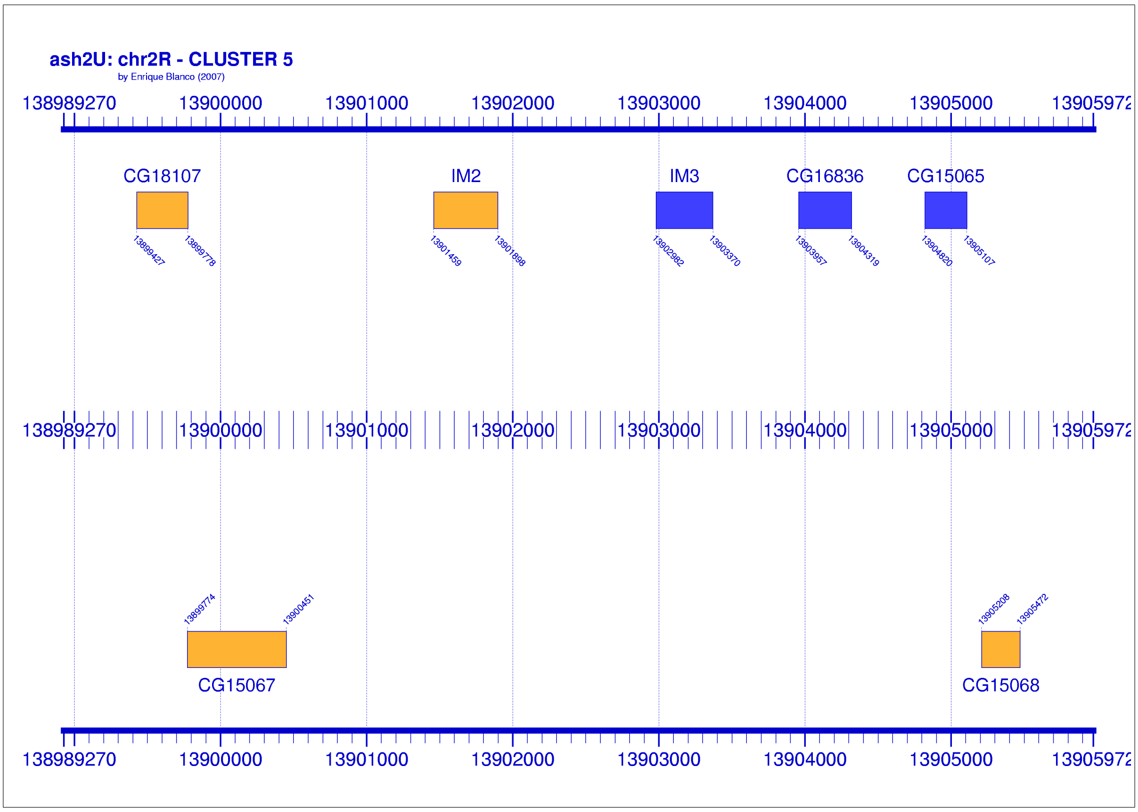

Enrique Blanco © 2007 — May 22, 2007

ash2U – chr3R: 10386703 - 10392190

Genomic components: 3 coregulated genes, 4 genes

CHR	Strand	Start	End	RefSeq	Name	Exons	Description
CHR3R	+	10386703	10387178	NM_142114	CG14850	1	CG14850-PA
CHR3R	+	10388083	10388540	NM_142115	CG14851	1	CG14851-PA
CHR3R	-	10390044	10390504	NM_169585	CG8087	1	CG8087-PA
CHR3R	+	10391667	10392190	NM_142116	CG14852	1	CG14852-PA

Cluster size: 5488 nucleotides

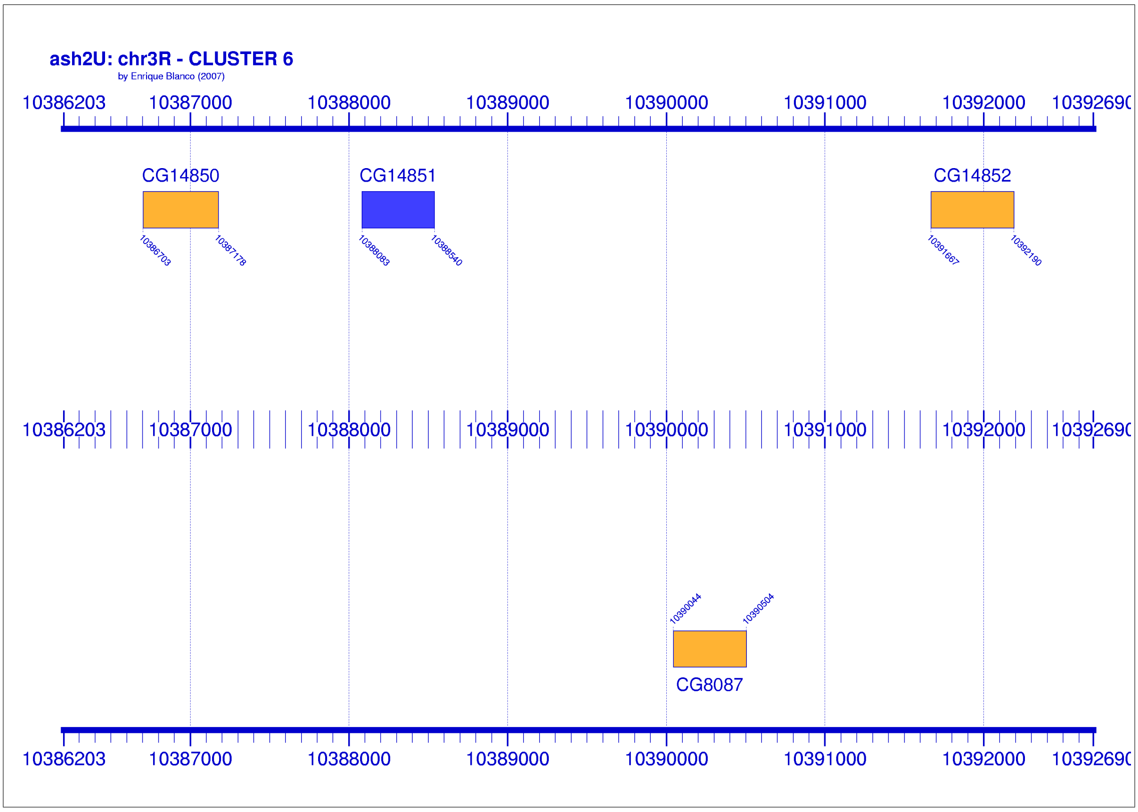

Enrique Blanco © 2007 — May 22, 2007

ash2D – chr3L: 9345665 - 9359229

Genomic components: 4 coregulated genes, 8 genes

CHR	Strand	Start	End	RefSeq	Name	Exons	Description
CHR3L	-	9345665	9346264	NM_079270	Hsp67Bc	1	Heat shock gene 67Bc CG4190-PA
CHR3L	+	9346877	9348916	NM_001031944	Hsp22	3	CG4460-PA, isoform A
CHR3L	+	9346877	9348916	NM_001031945	Hsp67Bb	3	CG4456-PB, isoform B
CHR3L	+	9349375	9350216	NM_140047	CG4461	1	CG4461-PA
CHR3L	-	9350364	9351374	NM_079273	Hsp26	1	Heat shock protein 26 CG4183-PA
CHR3L	-	9352049	9353386	NM_079274	Hsp67Ba	1	Heat shock gene 67Ba CG4167-PA
CHR3L	+	9355830	9356712	NM_079275	Hsp23	1	Heat shock protein 23 CG4463-PA
CHR3L	+	9358011	9359229	NM_079276	Hsp27	1	Heat shock protein 27 CG4466-PA

Cluster size: 13565 nucleotides

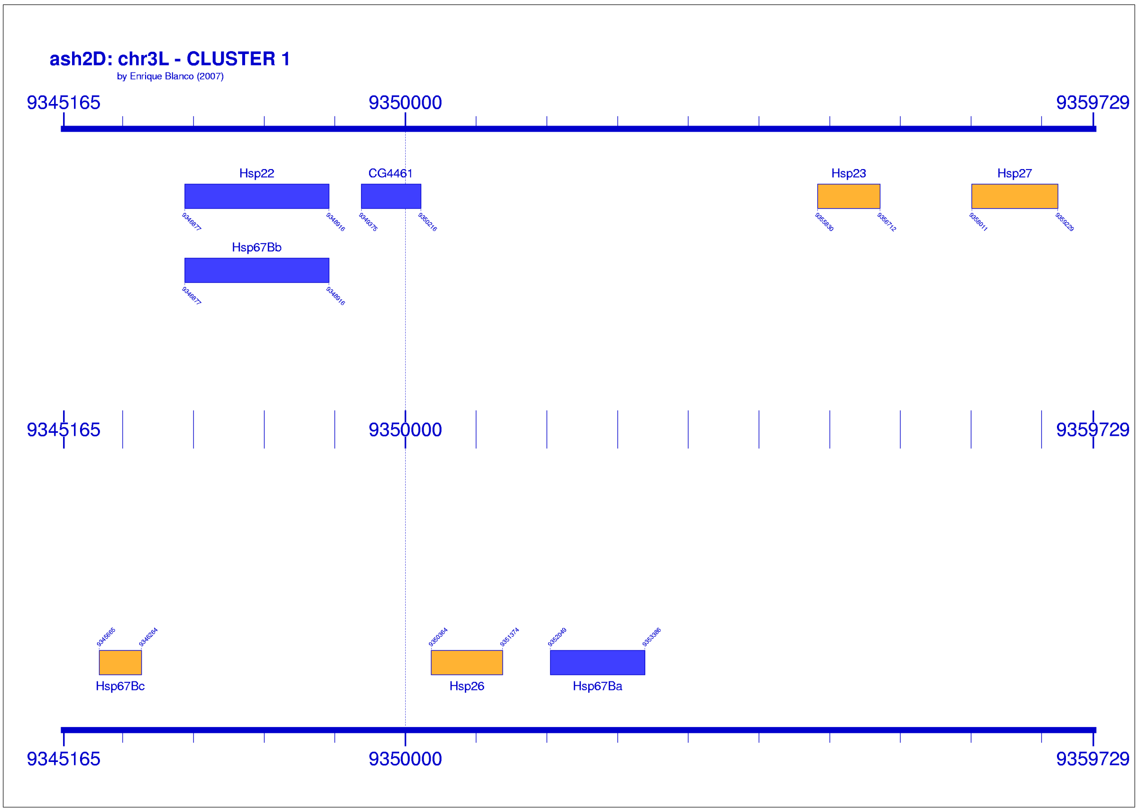

Enrique Blanco © 2007 — May 22, 2007

ash2D – chr3L: 11482913 - 11487349

Genomic components: 3 coregulated genes, 4 genes

CHR	Strand	Start	End	RefSeq	Name	Exons	Description
CHR3L	-	11482913	11483341	NM_057371	Sgs8	2	Salivary gland secretion 8 CG6132-PA
CHR3L	+	11483807	11484194	NM_057370	Sgs7	2	Salivary gland secretion 7 CG18087-PA
CHR3L	-	11484269	11484536	NM_206323	CG33272	1	CG33272-PA
CHR3L	+	11486167	11487349	NM_079300	Sgs3	2	Salivary gland secretion 3 CG11720-PA

Cluster size: 4437 nucleotides

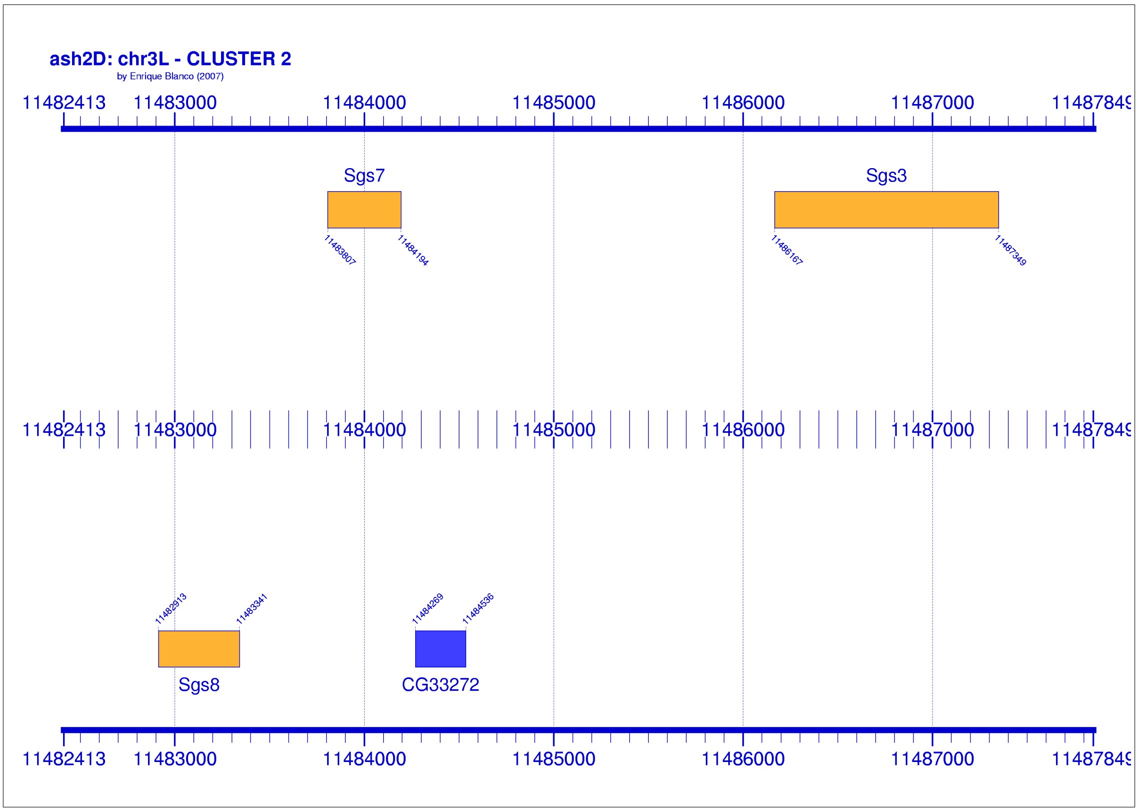

Enrique Blanco © 2007 — May 22, 2007

nurf – chr2L: 4937408 - 4958292

Genomic components: 3 coregulated genes, 9 genes

CHR	Strand	Start	End	RefSeq	Name	Exons	Description
CHR2L	+	4937408	4941415	NM_078751	Sgs1	2	Salivary gland secretion 1 CG3047-PA
CHR2L	+	4942747	4944305	NM_135035	CG14044	5	CG14044-PA
CHR2L	-	4944255	4945343	NM_078752	mRpL24	4	mitochondrial ribosomal protein L24 CG8849-PA
CHR2L	+	4945597	4948321	NM_080361	betaggt-I	4	beta subunit of type I geranylgeranyl
CHR2L	-	4948205	4949751	NM_001042867	CG8873	2	CG8873-PB, isoform B
CHR2L	-	4949946	4950763	NM_135037	Jon25Biii	1	Jonah 25Biii CG8871-PA
CHR2L	-	4952249	4953134	NM_135038	Jon25Bii	1	Jonah 25Bii CG8869-PA
CHR2L	-	4954071	4955353	NM_078753	Jon25Bi	1	Jonah 25Bi CG8867-PA, isoform A
CHR2L	+	4955518	4958292	NM_135039	Marcal1	7	Marcal1 CG3753-PA

Cluster size: 20885 nucleotides

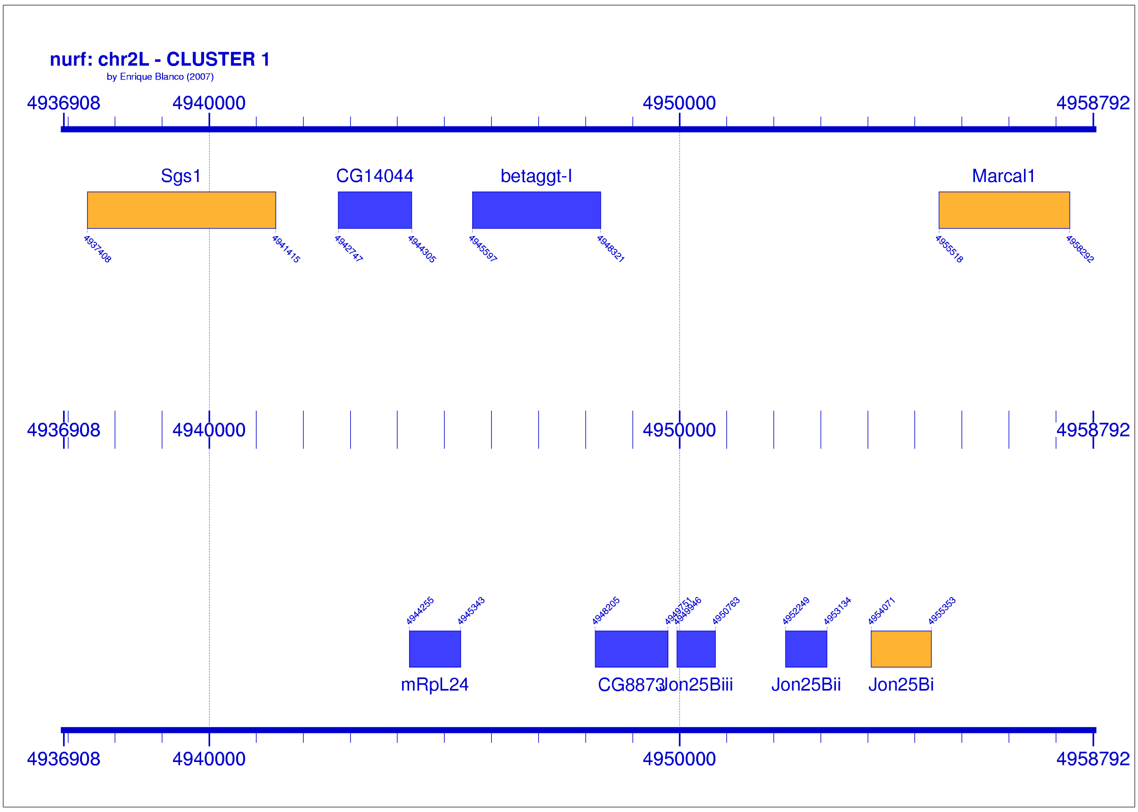

Enrique Blanco © 2007 — May 22, 2007

nurf – chr3L: 4453991 - 4468860

Genomic components: 3 coregulated genes, 4 genes

CHR	Strand	Start	End	RefSeq	Name	Exons	Description
CHR3L	+	4453991	4455942	NM_139651	CG11349	1	CG11349-PA
CHR3L	+	4461128	4462334	NM_139652	CG7465	2	CG7465-PA
CHR3L	+	4463757	4465126	NM_139653	CG11350	1	CG11350-PB
CHR3L	-	4466256	4468860	NM_139654	CG13722	3	CG13722-PA

Cluster size: 14870 nucleotides

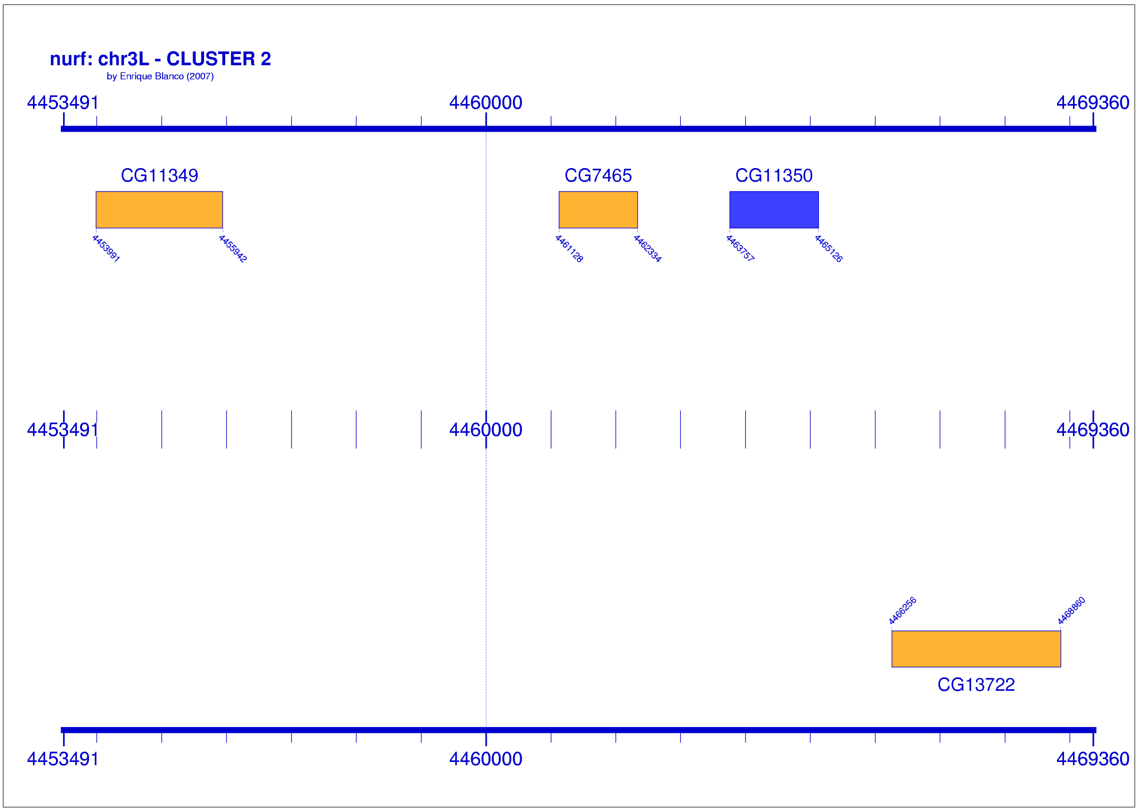

Enrique Blanco © 2007 — May 22, 2007

nurf – chr3L: 6097832 - 6117953

Genomic components: 4 coregulated genes, 10 genes

Chr	Strand	Start	End	RefSeq	Name	Exons	Description
chr3L	-	6097832	6102566	NM_001014570	l(3)mbn	7	lethal (3) malignant blood neoplasm CG12755-PC,
chr3L	-	6104332	6104933	NM_144405	CG18779	2	CG18779-PA
chr3L	-	6105259	6106056	NM_144404	CG18778	2	CG18778-PA
chr3L	-	6106936	6107547	NM_057924	Lcp65Ag2	2	Lcp65Ag2 CG10534-PA
chr3L	-	6108643	6109217	NM_057925	Lcp65Ag1	2	Lcp65Ag1 CG10530-PA
chr3L	-	6110251	6110718	NM_057926	Lcp65Af	2	Lcp65Af CG10533-PA
chr3L	-	6111667	6112025	NM_176290	Lcp65Ae	2	Lcp65Ae CG10529-PA
chr3L	-	6113169	6113920	NM_168158	CG32405	2	CG32405-PA
chr3L	-	6115912	6116330	NM_168159	CG32404	2	CG32404-PA
chr3L	+	6117353	6117953	NM_057930	Lcp65Ad	2	Lcp65Ad CG6955-PA

Cluster size: 20122 nucleotides

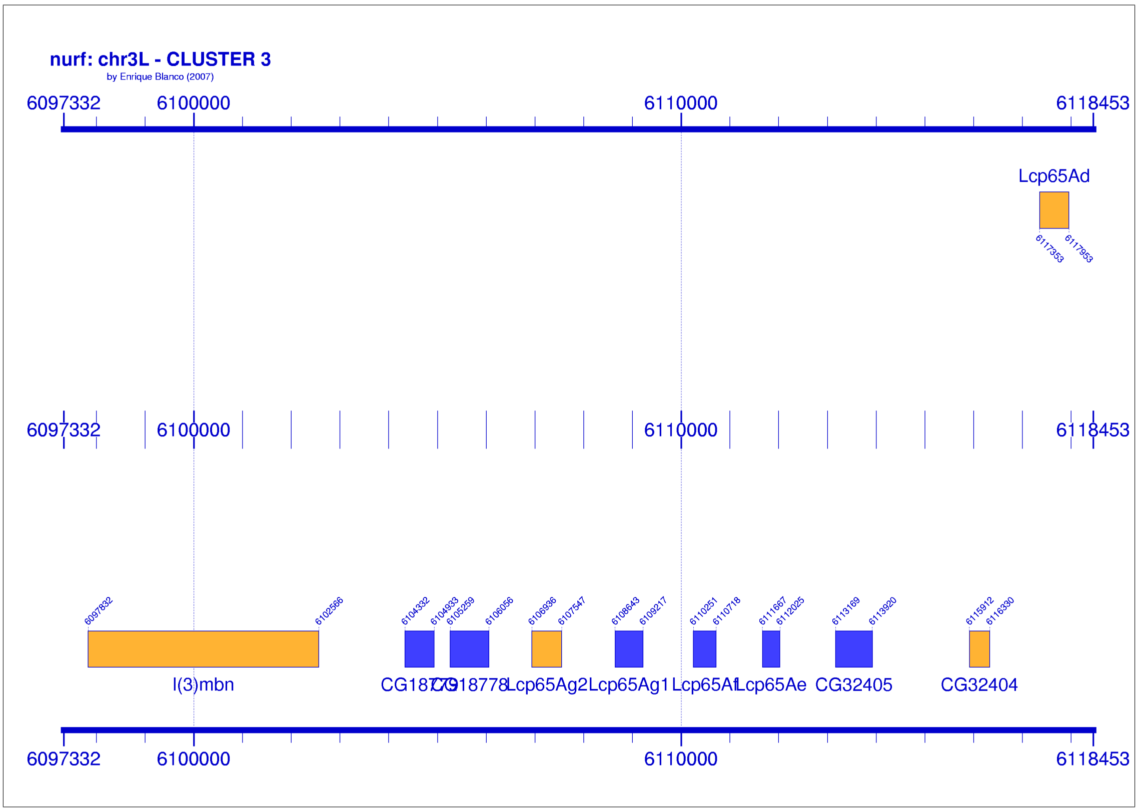

Enrique Blanco © 2007 — May 22, 2007

nurf – chr3L: 9345665 - 9359229

Genomic components: 4 coregulated genes, 8 genes

CHR	Strand	Start	End	RefSeq	Name	Exons	Description
CHR3L	-	9345665	9346264	NM_079270	Hsp67Bc	1	Heat shock gene 67Bc CG4190-PA
CHR3L	+	9346877	9348916	NM_001031944	Hsp22	3	CG4460-PA, isoform A
CHR3L	+	9346877	9348916	NM_001031945	Hsp67Bb	3	CG4456-PB, isoform B
CHR3L	+	9349375	9350216	NM_140047	CG4461	1	CG4461-PA
CHR3L	-	9350364	9351374	NM_079273	Hsp26	1	Heat shock protein 26 CG4183-PA
CHR3L	-	9352049	9353386	NM_079274	Hsp67Ba	1	Heat shock gene 67Ba CG4167-PA
CHR3L	+	9355830	9356712	NM_079275	Hsp23	1	Heat shock protein 23 CG4463-PA
CHR3L	+	9358011	9359229	NM_079276	Hsp27	1	Heat shock protein 27 CG4466-PA

Cluster size: 13565 nucleotides

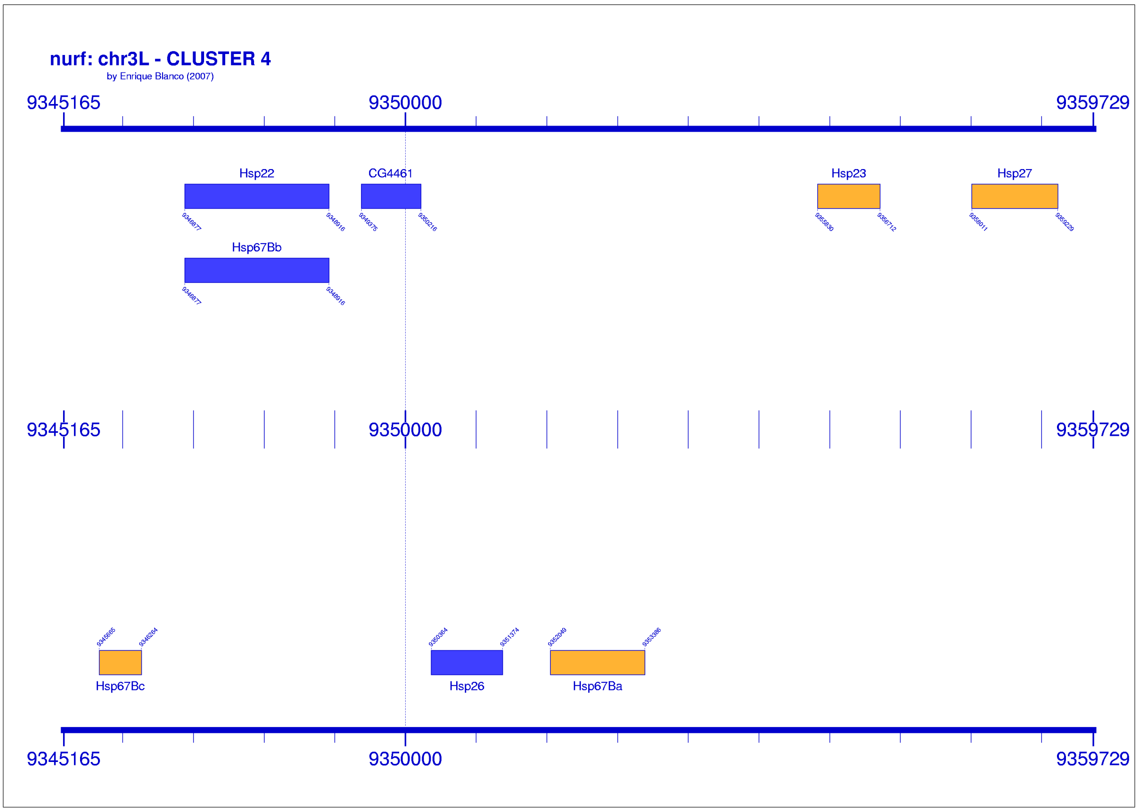

Enrique Blanco © 2007 — May 22, 2007

nurf – chr3L: 11482913 - 11487349

Genomic components: 3 coregulated genes, 4 genes

CHR	Strand	Start	End	RefSeq	Name	Exons	Description
CHR3L	-	11482913	11483341	NM_057371	Sgs8	2	Salivary gland secretion 8 CG6132-PA
CHR3L	+	11483807	11484194	NM_057370	Sgs7	2	Salivary gland secretion 7 CG18087-PA
CHR3L	-	11484269	11484536	NM_206323	CG33272	1	CG33272-PA
CHR3L	+	11486167	11487349	NM_079300	Sgs3	2	Salivary gland secretion 3 CG11720-PA

Cluster size: 4437 nucleotides

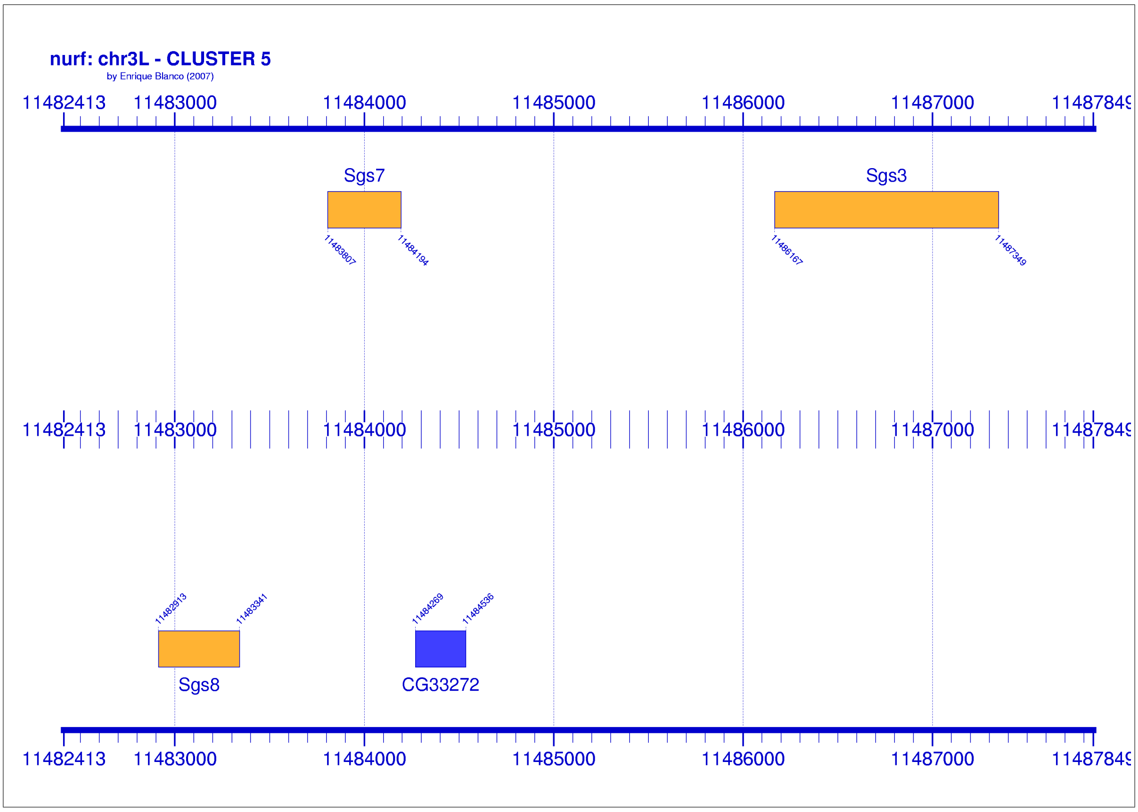

Enrique Blanco © 2007 — May 22, 2007

nurf – chr2R: 7902885 - 7921019

Genomic components: 3 coregulated genes, 5 genes

CHR	Strand	Start	End	RefSeq	Name	Exons	Description
CHR2R	+	7902885	7905964	NM_136928	CG8502	7	CG8502-PA, isoform A
CHR2R	-	7906941	7907561	NM_136929	CG8836	3	CG8836-PA
CHR2R	+	7909389	7910747	NM_078987	Or49a	4	Odorant receptor 49a CG13158-PA
CHR2R	+	7910848	7915011	NM_165895	CG30048	3	CG30048-PA, isoform A
CHR2R	+	7918121	7921019	NM_136930	CG8505	3	CG8505-PA

Cluster size: 18135 nucleotides

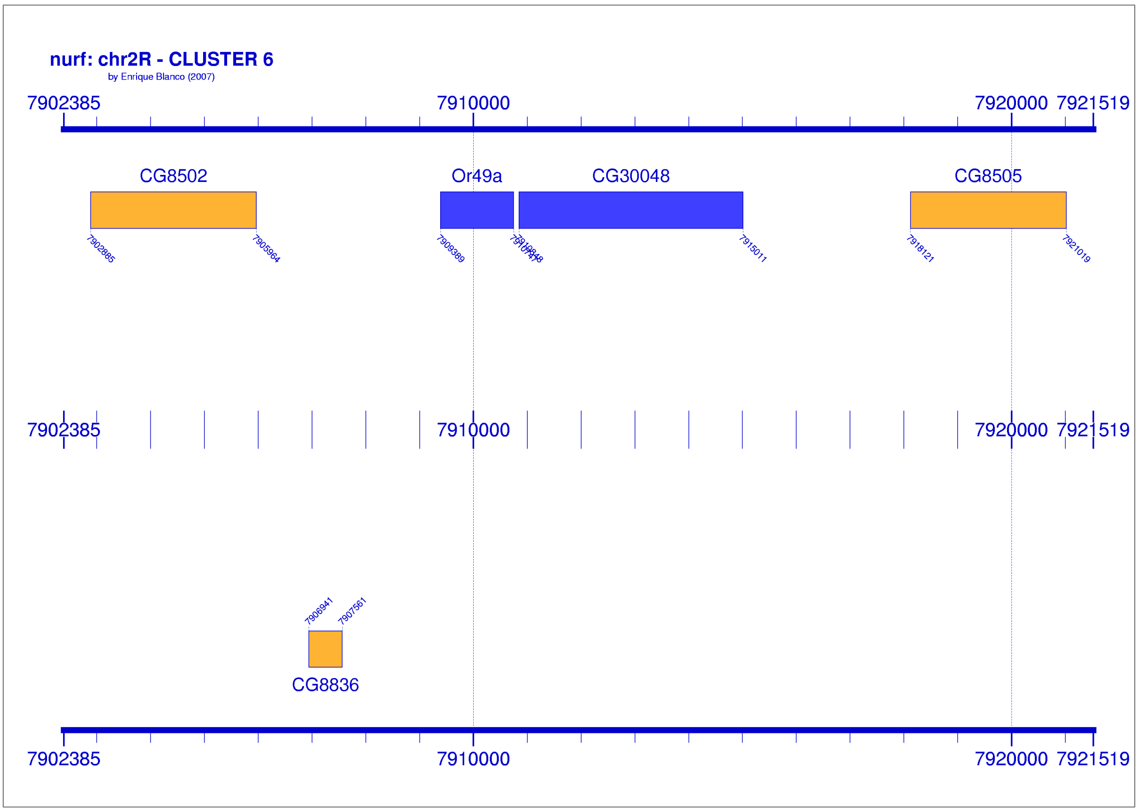

Enrique Blanco © 2007 — May 22, 2007

nurf – chrX: 4615792 - 4628201

Genomic components: 3 coregulated genes, 3 genes

Chr	Strand	Start	End	RefSeq	Name	Exons	Description
CHRX	-	4615792	4616754	NM_131957	Bteb2	1	Bteb2 CG2932-PA
CHRX	-	4621194	4622612	NM_131958	CG2871	1	CG2871-PA
CHRX	-	4627553	4628201	NM_131959	CG15471	2	CG15471-PA

Cluster size: 12410 nucleotides

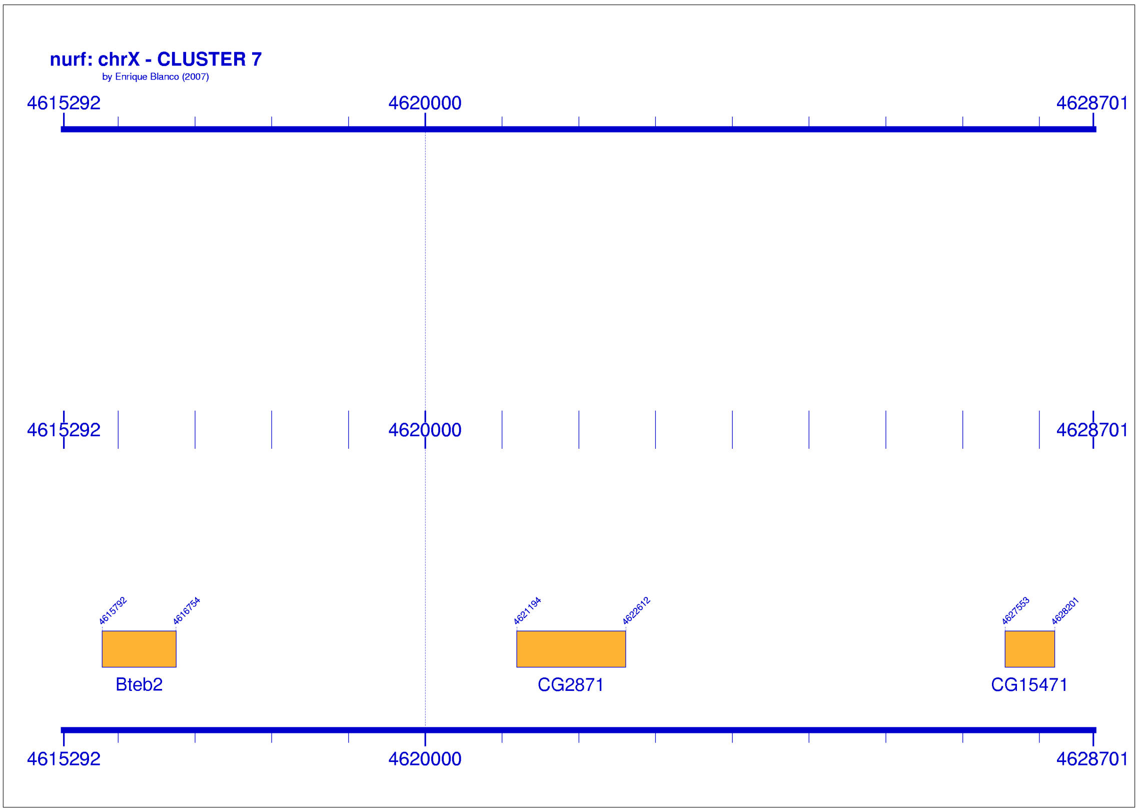

Enrique Blanco © 2007 — May 22, 2007

dmyc – chr2L: 7994833 - 8009459

Genomic components: 3 coregulated genes, 5 genes

CHR	Strand	Start	End	RefSeq	Name	Exons	Description
CHR2L	-	7994833	7998139	NM_135325	CG7227	6	CG7227-PA
CHR2L	+	7998933	7999743	NM_001038793	CG7224	1	CG7224-PB, isoform B
CHR2L	-	7999669	8000000	NM_135327	Wwox	1	Wwox CG7221-PA
CHR2L	-	8004650	8007132	NM_135328	CG7219	2	CG7219-PA
CHR2L	-	8008441	8009459	NM_135329	CG12560	4	CG12560-PA

Cluster size: 14627 nucleotides

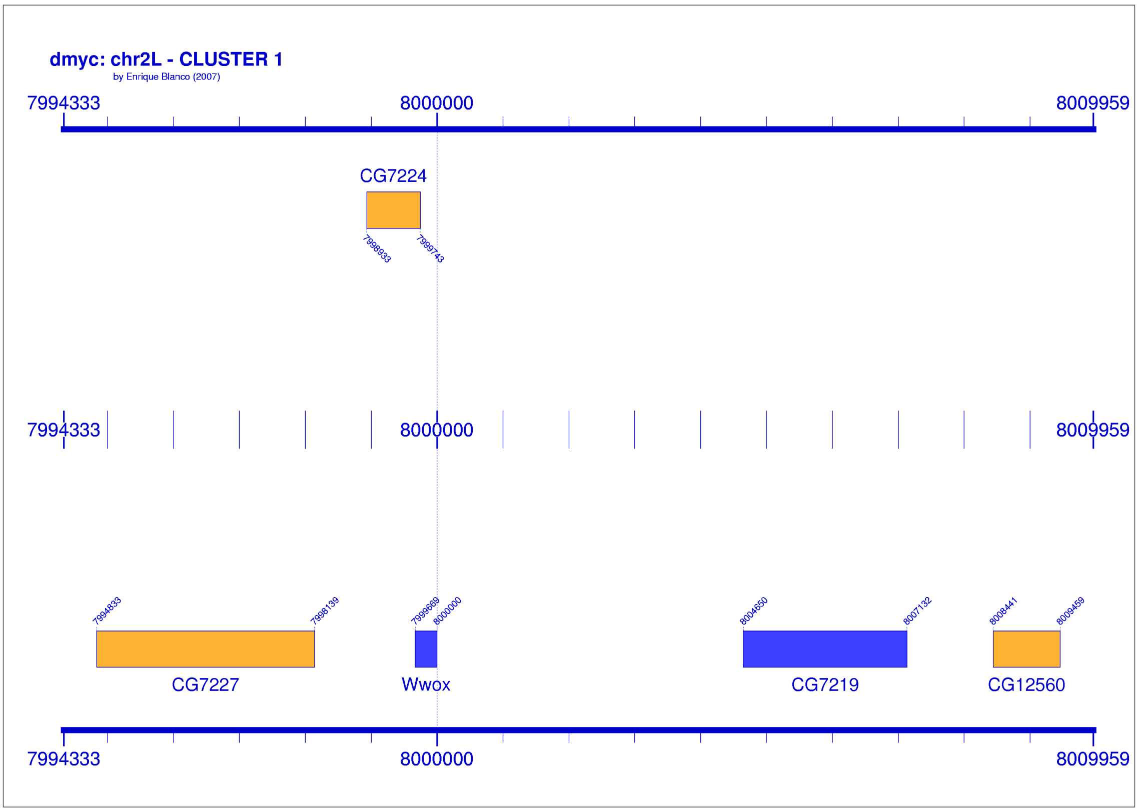

Enrique Blanco © 2007 — May 22, 2007

dmyc – chr3L: 4429174 - 4462334

Genomic components: 5 coregulated genes, 10 genes

CHR	Strand	Start	End	RefSeq	Name	Exons	Description
CHR3L	+	4429174	4430664	NM_139646	CG12607	3	CG12607-PB
CHR3L	-	4438168	4438983	NM_139647	CG11345	2	CG11345-PA
CHR3L	-	4440039	4441160	NM_139648	CG15022	2	CG15022-PA
CHR3L	-	4441593	4442099	NM_139649	CG15023	1	CG15023-PA
CHR3L	-	4443894	4444185	NM_144079	CG15024	1	CG15024-PA
CHR3L	-	4446072	4447704	NM_168087	CG32241	2	CG32241-PA
CHR3L	+	4449198	4451878	NM_168088	CG32249	2	CG32249-PA
CHR3L	+	4452493	4453229	NM_168089	CG32248	2	CG32248-PA
CHR3L	+	4453991	4455942	NM_139651	CG11349	1	CG11349-PA
CHR3L	+	4461128	4462334	NM_139652	CG7465	2	CG7465-PA

Cluster size: 33161 nucleotides

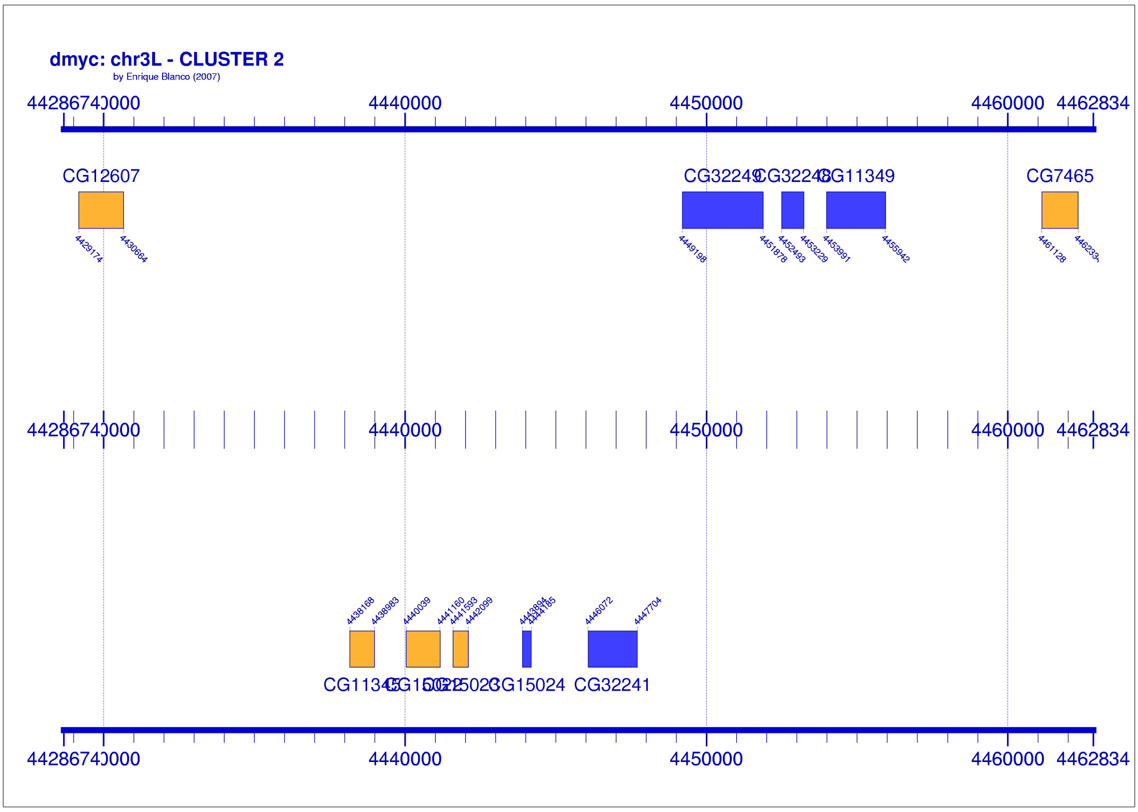

Enrique Blanco © 2007 — May 22, 2007

dmyc – chr3L: 6106936 - 6131320

Genomic components: 4 coregulated genes, 14 genes

CHR	Strand	Start	End	RefSeq	Name	Exons	Description
CHR3L	-	6106936	6107547	NM_057924	Lcp65Ag2	2	Lcp65Ag2 CG10534-PA
CHR3L	-	6108643	6109217	NM_057925	Lcp65Ag1	2	Lcp65Ag1 CG10530-PA
CHR3L	-	6110251	6110718	NM_057926	Lcp65Af	2	Lcp65Af CG10533-PA
CHR3L	-	6111667	6112025	NM_176290	Lcp65Ae	2	Lcp65Ae CG10529-PA
CHR3L	-	6113169	6113920	NM_168158	CG32405	2	CG32405-PA
CHR3L	-	6115912	6116330	NM_168159	CG32404	2	CG32404-PA
CHR3L	+	6117353	6117953	NM_057930	Lcp65Ad	2	Lcp65Ad CG6955-PA
CHR3L	+	6118770	6119345	NM_057931	Lcp65Ac	2	Lcp65Ac CG6956-PA
CHR3L	+	6120617	6121036	NM_176291	Lcp65Ab2	1	Lcp65Ab2 CG18773-PA
CHR3L	+	6120668	6120981	NM_080075	Lcp65Ab1	1	Lcp65Ab1 CG32400-PA
CHR3L	-	6121477	6121928	NM_144403	CG18777	2	CG18777-PA
CHR3L	+	6125279	6125586	NM_057932	Lcp65Aa	1	Lcp65Aa CG7287-PA
CHR3L	-	6125994	6126692	NM_057934	Acp65Aa	2	Acp65Aa CG10297-PA
CHR3L	-	6130541	6131320	NM_139773	CG13297	2	CG13297-PA

Cluster size: 24385 nucleotides

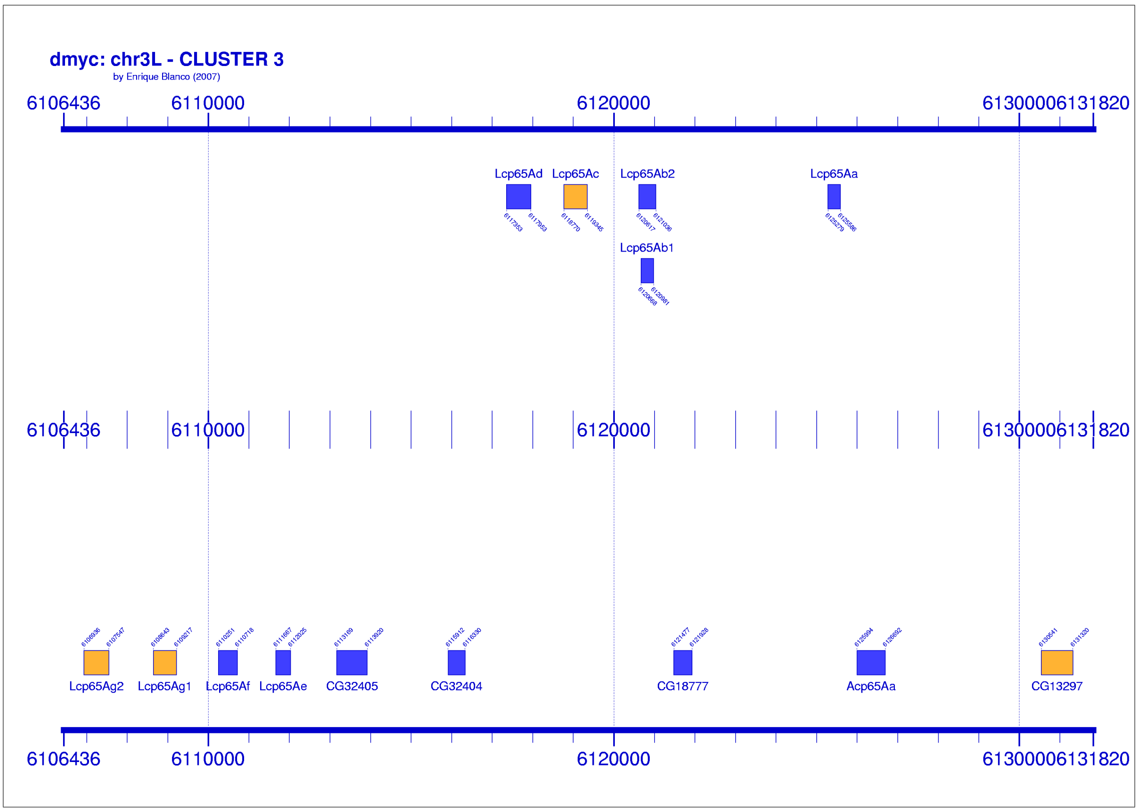

Enrique Blanco © 2007 — May 22, 2007

dmyc – chr3L: 16250025 - 16289521

Genomic components: 6 coregulated genes, 15 genes

CHR	Strand	Start	End	RefSeq	Name	Exons	Description
CHR3L	-	16250025	16251401	NM_140605	CG13047	2	CG13047-PA
CHR3L	-	16252973	16253474	NM_140606	CG13046	1	CG13046-PA
CHR3L	-	16255494	16256056	NM_140607	CG13045	1	CG13045-PA
CHR3L	-	16259969	16260799	NM_140608	CG4962	2	CG4962-PA
CHR3L	+	16266728	16267286	NM_140609	CG4982	2	CG4982-PA
CHR3L	-	16267749	16268419	NM_140610	CG13044	2	CG13044-PA
CHR3L	-	16270483	16271125	NM_140611	CG13043	1	CG13043-PA
CHR3L	+	16271905	16272355	NM_140612	CG13063	2	CG13063-PA
CHR3L	-	16273258	16273674	NM_140613	CG13042	2	CG13042-PA
CHR3L	+	16274198	16276577	NM_168658	CG32160	2	CG32160-PA
CHR3L	+	16279434	16280204	NM_140614	CG13062	3	CG13062-PA
CHR3L	+	16283283	16283949	NM_144453	Nplp3	2	Neuropeptide-like precursor 3 CG13061-PA
CHR3L	-	16284212	16284779	NM_140615	CG13041	2	CG13041-PA
CHR3L	+	16285119	16285721	NM_140616	CG13060	2	CG13060-PA
CHR3L	+	16288989	16289521	NM_140617	CG13059	2	CG13059-PA

Cluster size: 39497 nucleotides

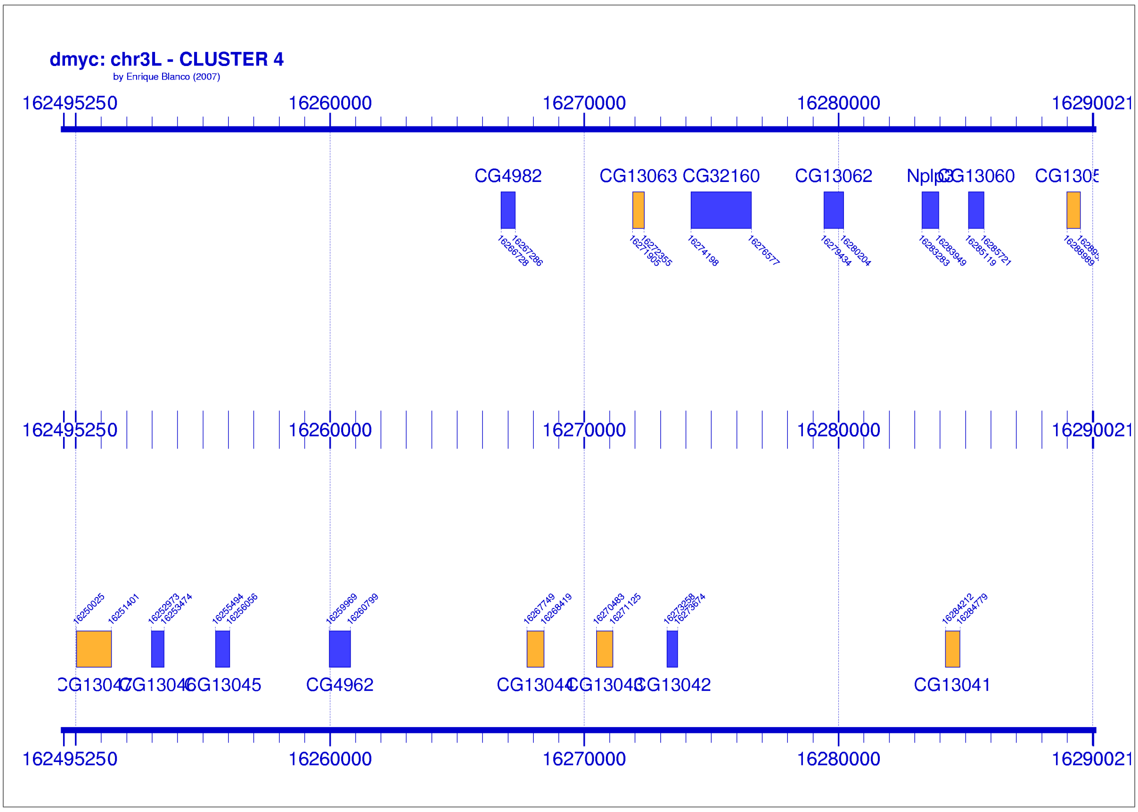

Enrique Blanco © 2007 — May 22, 2007

dmyc – chr3L: 21664564 - 21672175

Genomic components: 3 coregulated genes, 4 genes

CHR	Strand	Start	End	RefSeq	Name	Exons	Description
CHR3L	-	21664564	21665388	NM_141097	CG14569	1	CG14569-PA
CHR3L	-	21666650	21667325	NM_141098	CG14568	1	CG14568-PA
CHR3L	+	21667971	21668550	NM_141099	CG14573	1	CG14573-PA
CHR3L	-	21671604	21672175	NM_141100	CG14567	1	CG14567-PA

Cluster size: 7612 nucleotides

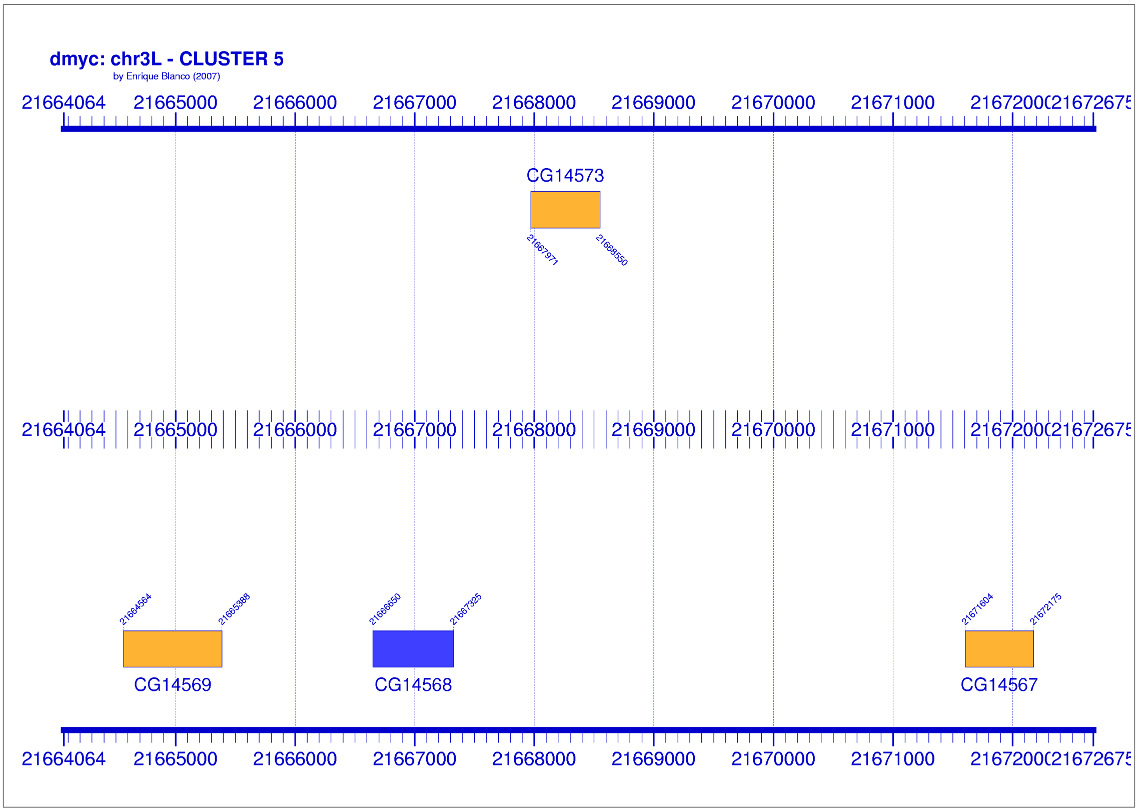

Enrique Blanco © 2007 — May 22, 2007

dmyc – chr3R: 22444844 - 22466776

Genomic components: 6 coregulated genes, 12 genes

CHR	Strand	Start	End	RefSeq	Name	Exons	Description
CHR3R	+	22444844	22445952	NM_143227	CG5468	2	CG5468-PA
CHR3R	-	22446329	22447082	NM_143228	CG14240	1	CG14240-PA
CHR3R	-	22448172	22449270	NM_143229	CG6478	2	CG6478-PA
CHR3R	-	22450301	22451374	NM_170279	CG6447	2	CG6447-PA, isoform A
CHR3R	-	22451731	22452419	NM_143230	CG6452	1	CG6452-PA
CHR3R	-	22453687	22454429	NM_143231	CG6460	1	CG6460-PA
CHR3R	+	22455578	22456500	NM_143232	CG5471	1	CG5471-PA
CHR3R	+	22457594	22458678	NM_170281	CG5476	1	CG5476-PA
CHR3R	+	22459455	22460436	NM_143233	CG31080	2	CG31080-PA
CHR3R	+	22460595	22461638	NM_170282	CG31081	2	CG31081-PA
CHR3R	+	22462435	22463182	NM_143234	CG14242	2	CG14242-PA
CHR3R	+	22465747	22466776	NM_143235	CG14243	2	CG14243-PA

Cluster size: 21933 nucleotides

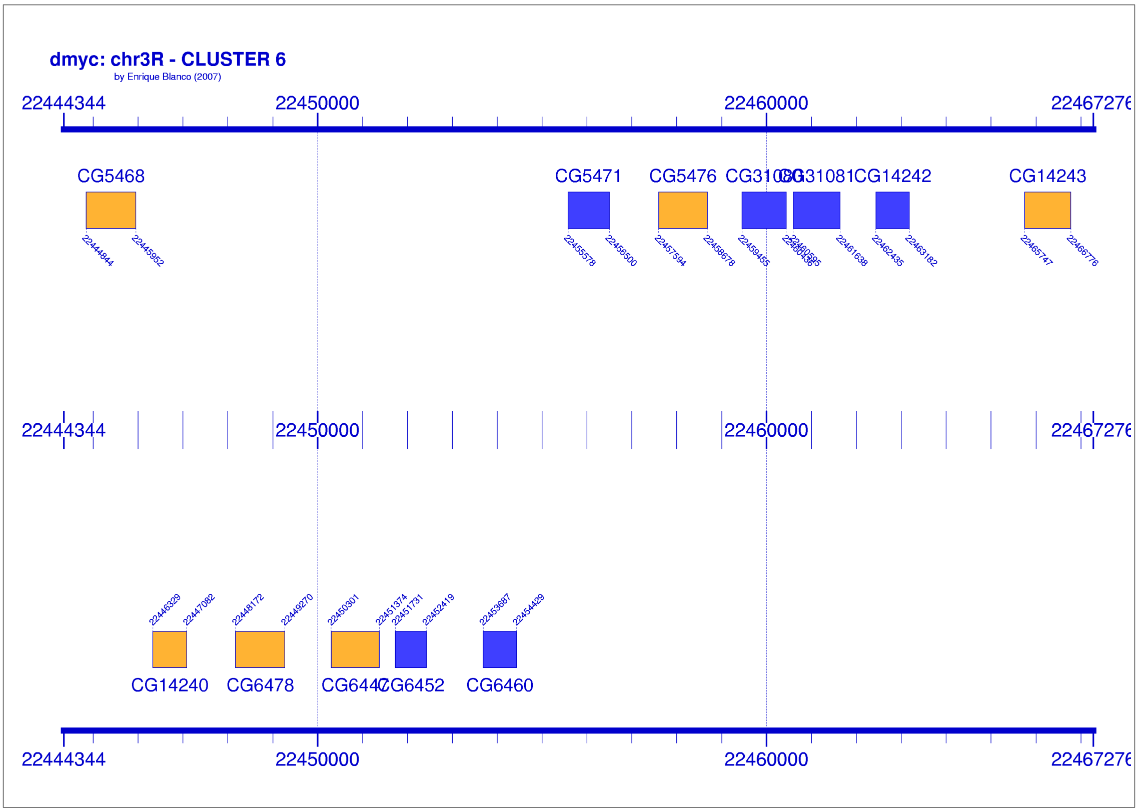

Enrique Blanco © 2007 — May 22, 2007

ash1U – chr3L: 6104332 - 6124799

Genomic components: 4 coregulated genes, 13 genes

Chr	Strand	Start	End	RefSeq	Name	Exons	Description
chr3L	-	6104332	6104933	NM_144405	CG18779	2	CG18779-PA
chr3L	-	6105259	6106056	NM_144404	CG18778	2	CG18778-PA
chr3L	-	6106936	6107547	NM_057924	Lcp65Ag2	2	Lcp65Ag2 CG10534-PA
chr3L	-	6108643	6109217	NM_057925	Lcp65Ag1	2	Lcp65Ag1 CG10530-PA
chr3L	-	6110251	6110718	NM_057926	Lcp65Af	2	Lcp65Af CG10533-PA
chr3L	-	6111667	6112025	NM_176290	Lcp65Ae	2	Lcp65Ae CG10529-PA
chr3L	-	6113169	6113920	NM_168158	CG32405	2	CG32405-PA
chr3L	-	6115912	6116330	NM_168159	CG32404	2	CG32404-PA
chr3L	+	6117353	6117953	NM_057930	Lcp65Ad	2	Lcp65Ad CG6955-PA
chr3L	+	6118770	6119345	NM_057931	Lcp65Ac	2	Lcp65Ac CG6956-PA
chr3L	+	6120617	6121036	NM_176291	Lcp65Ab2	1	Lcp65Ab2 CG18773-PA
chr3L	+	6120668	6120981	NM_080075	Lcp65Ab1	1	Lcp65Ab1 CG32400-PA
chr3L	-	6121477	6121928	NM_144403	CG18777	2	CG18777-PA

Cluster size: 20468 nucleotides

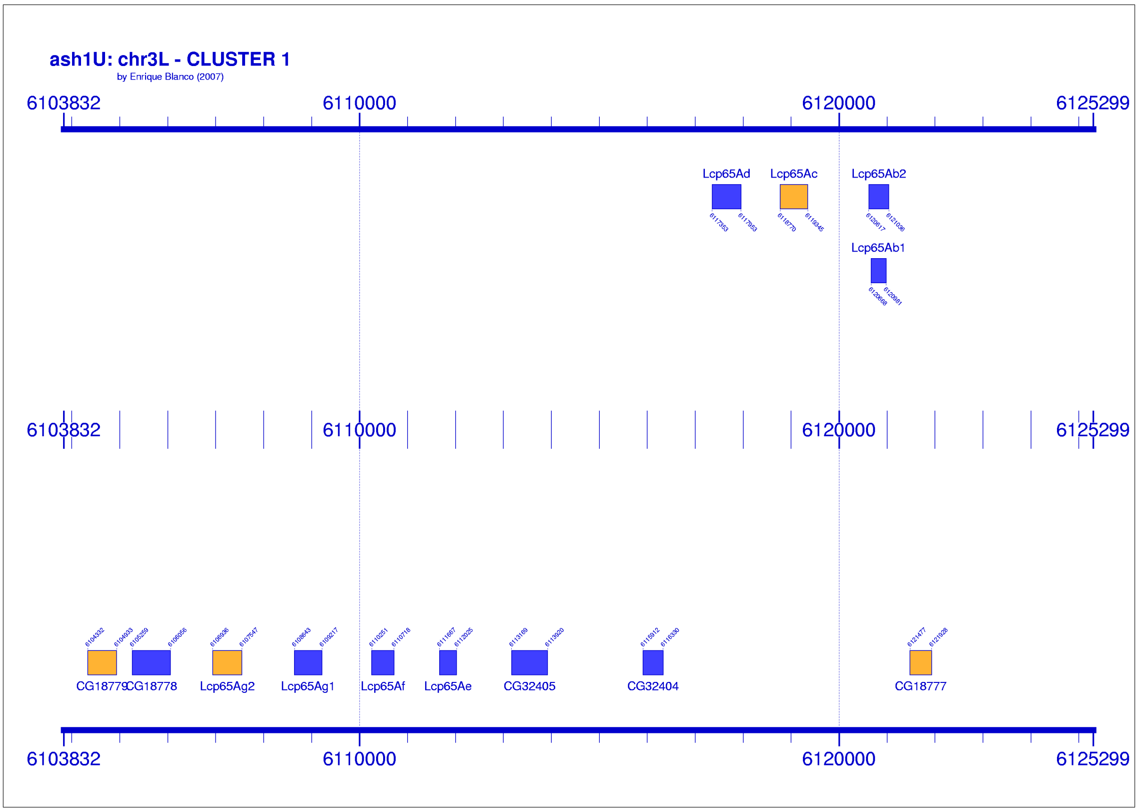

Enrique Blanco © 2007 — June 27, 2007

ash1U – chr3L: 15015516 - 15020824

Genomic components: 3 coregulated genes, 4 genes

CHR	Strand	Start	End	RefSeq	Name	Exons	Description
CHR3L	-	15015516	15016064	NM_140480	CG12310	1	CG12310-PA
CHR3L	-	15017322	15017734	NM_140481	CG13461	1	CG13461-PA
CHR3L	-	15018826	15019392	NM_140482	CG18649	1	CG18649-PA
CHR3L	+	15020029	15020824	NM_140483	CG13463	1	CG13463-PA

Cluster size: 5309 nucleotides

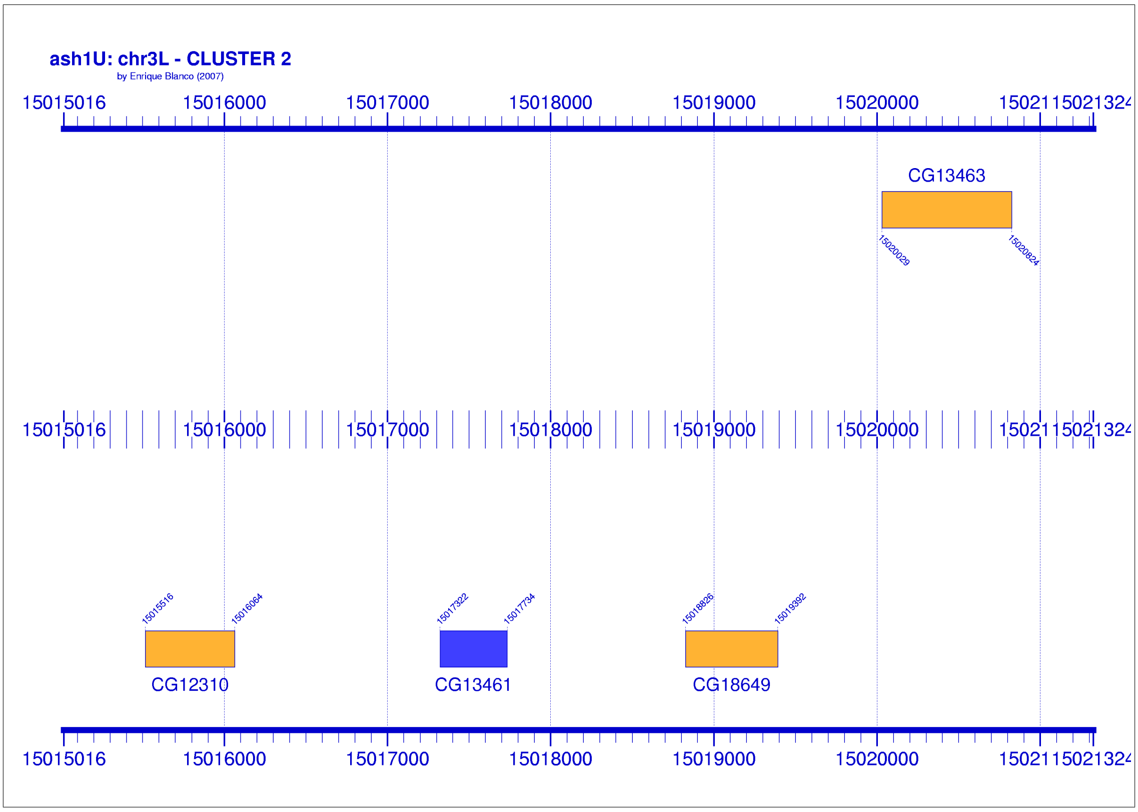

Enrique Blanco © 2007 — June 27, 2007

ash1U – chr2R: 3951207 - 3967595

Genomic components: 3 coregulated genes, 5 genes

CHR	Strand	Start	End	RefSeq	Name	Exons	Description
CHR2R	-	3951207	3954592	NM_136536	Cyp4ad1	9	Cyp4ad1 CG2110-PA
CHR2R	+	3956524	3958982	NM_057769	Cyp4e2	6	Cytochrome P450-4e2 CG2060-PA
CHR2R	+	3959281	3961558	NM_080032	Cyp4e1	6	Cytochrome P450-4e1 CG2062-PA
CHR2R	-	3961476	3963712	NM_057279	LvpH	3	Larval visceral protein H CG8696-PA
CHR2R	+	3965444	3967595	NM_057277	LvpD	3	Larval visceral protein D CG8694-PA

Cluster size: 16389 nucleotides

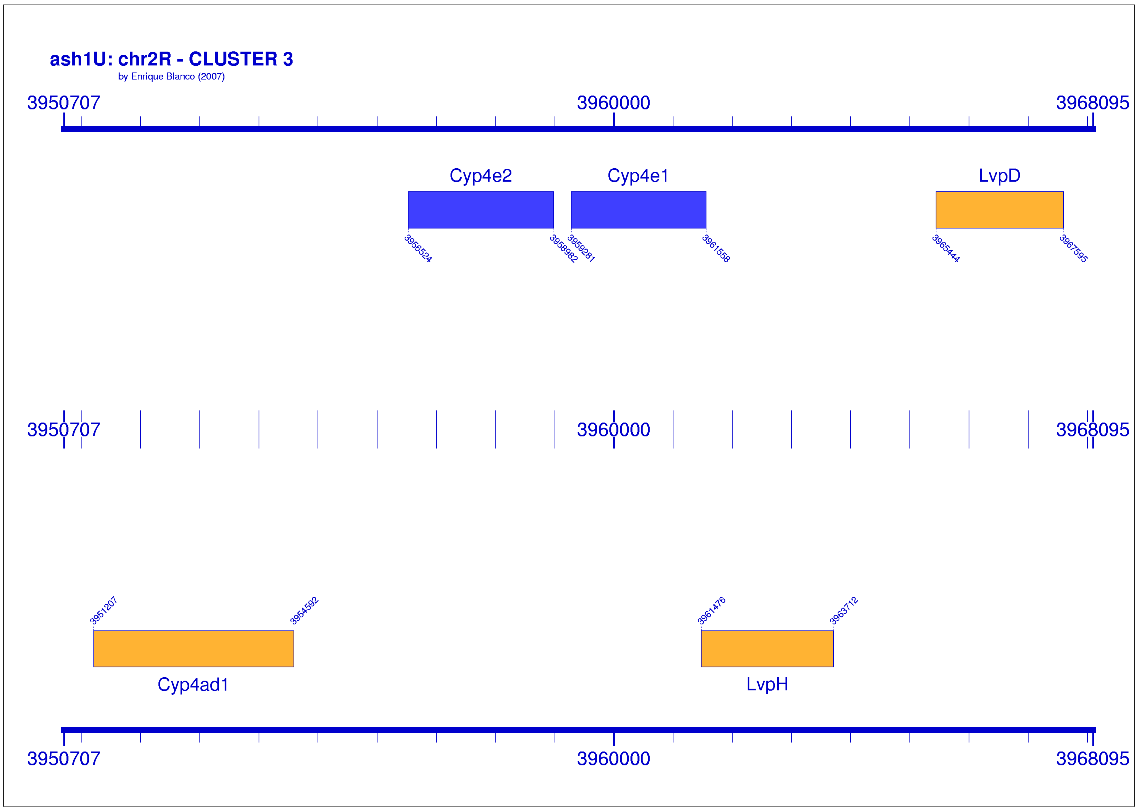

Enrique Blanco © 2007 — June 27, 2007

ash1U – chr3R: 8197719 - 8204940

Genomic components: 3 coregulated genes, 6 genes

Chr	Strand	Start	End	RefSeq	Name	Exons	Description
chr3R	+	8197719	8198366	NM_080173	GstD2	1	Glutathione S transferase D2 CG4181-PA
chr3R	+	8198785	8199535	NM_176479	GstD3	1	Glutathione S transferase D3 CG4381-PA
chr3R	+	8199823	8200561	NM_080174	GstD4	1	Glutathione S transferase D4 CG11512-PA
chr3R	+	8201485	8202134	NM_080175	GstD5	1	Glutathione S transferase D5 CG12242-PA
chr3R	+	8202893	8203539	NM_080176	GstD6	1	Glutathione S transferase D6 CG4423-PA
chr3R	+	8204267	8204940	NM_080375	GstD7	1	Glutathione S transferase D7 CG4371-PA

Cluster size: 7222 nucleotides

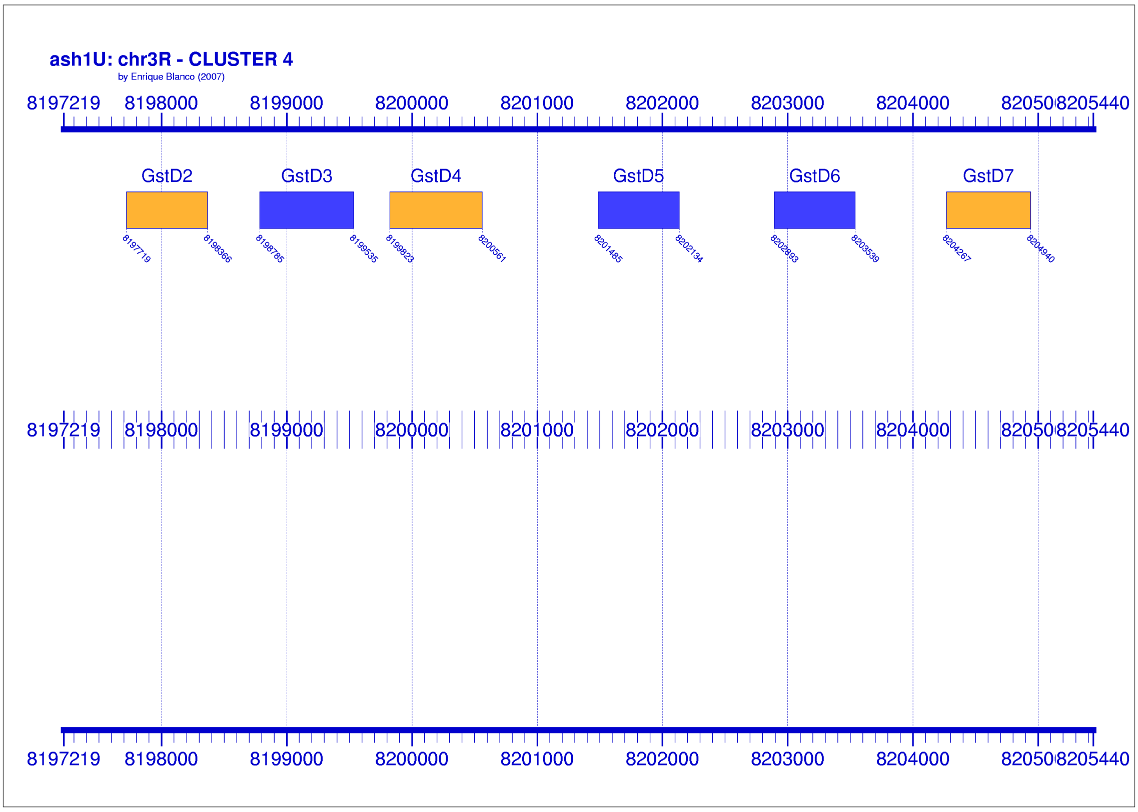

Enrique Blanco © 2007 — June 27, 2007

ash1U – chr3R: 14551120 - 14558756

Genomic components: 3 coregulated genes, 3 genes

CHR	Strand	Start	End	RefSeq	Name	Exons	Description
CHR3R	-	14551120	14552156	NM_142490	CG7714	2	CG7714-PA
CHR3R	-	14553294	14554190	NM_142491	CG7715	2	CG7715-PA
CHR3R	+	14558422	14558756	NM_142492	CG14302	2	CG14302-PA

Cluster size: 7637 nucleotides

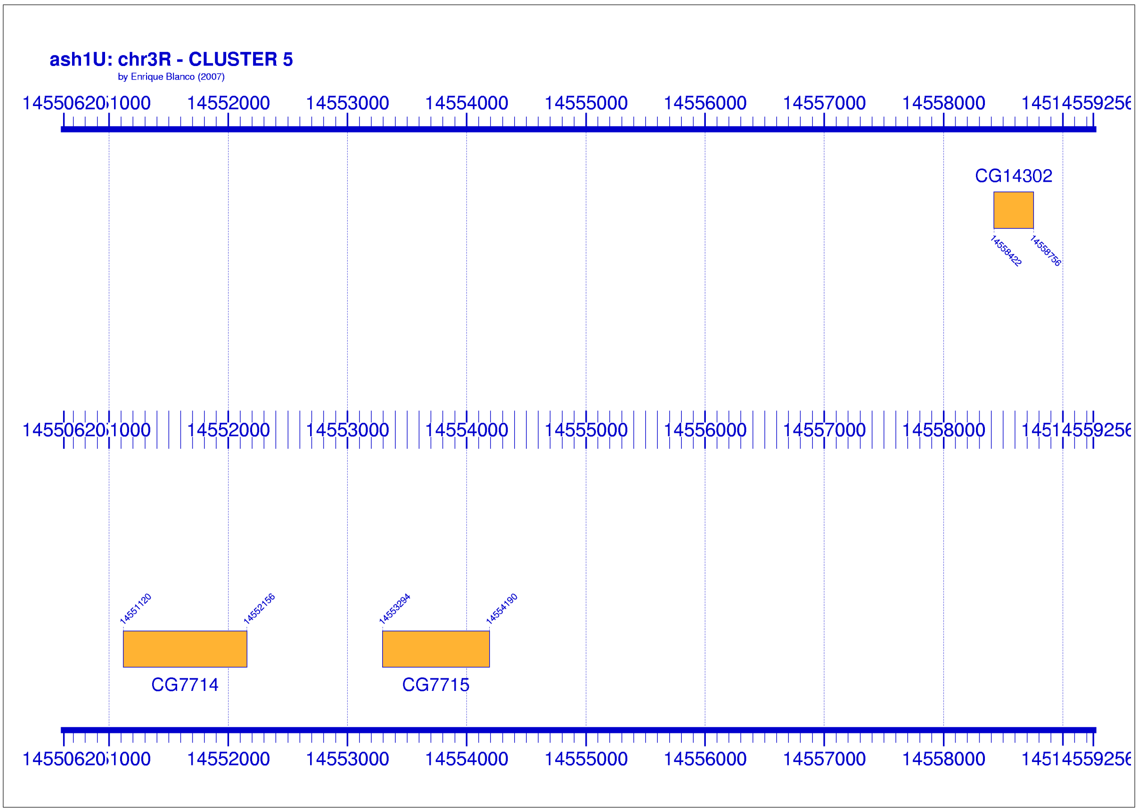

Enrique Blanco © 2007 — June 27, 2007

ash1U – chr3R: 14960880 - 14985924

Genomic components: 3 coregulated genes, 5 genes

CHR	Strand	Start	End	RefSeq	Name	Exons	Description
CHR3R	+	14960880	14962858	NM_142526	Cyp12a4	4	Cyp12a4 CG6042-PA
CHR3R	-	14964788	14973629	NM_142527	CG5629	5	CG5629-PB, isoform B
CHR3R	-	14974350	14985600	NM_001032023	CG11779	7	CG11779-PC, isoform C
CHR3R	-	14974353	14979402	NM_142528	CG5835	4	CG5835-PA
CHR3R	+	14982961	14985924	NM_057310	nos	3	nanos CG5637-PA

Cluster size: 25045 nucleotides

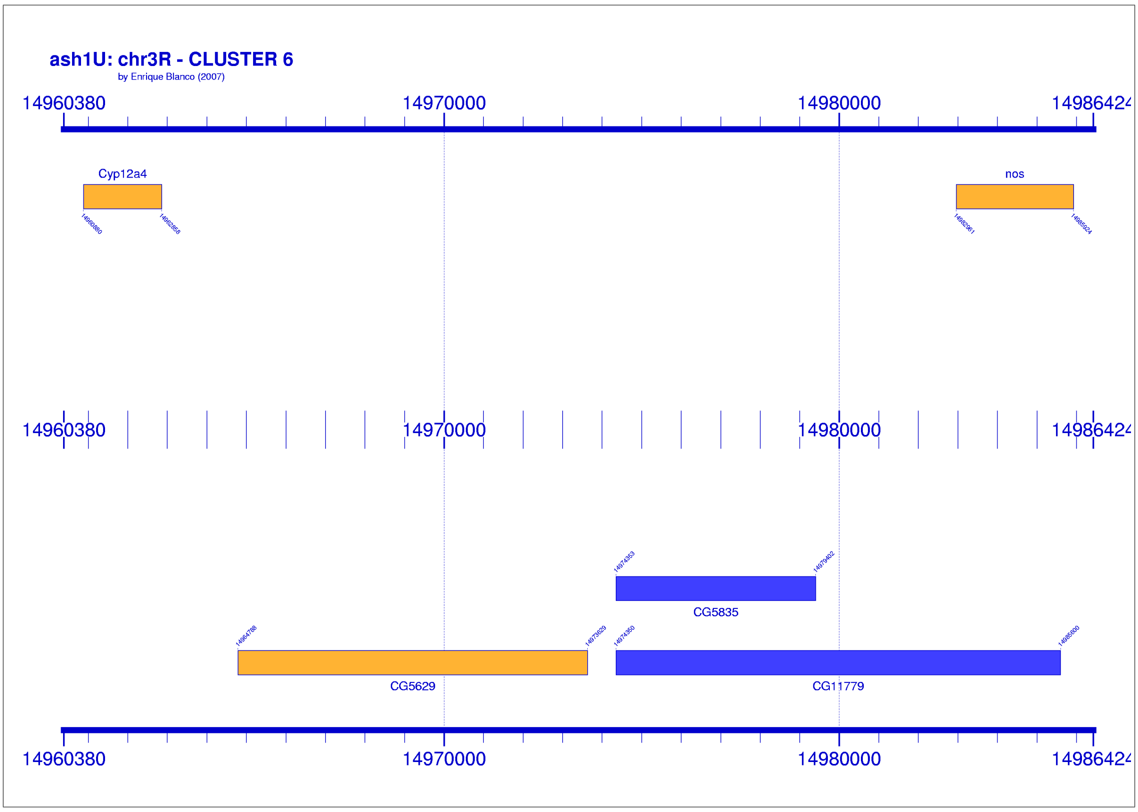

Enrique Blanco © 2007 — June 27, 2007

ash1U – chr3R: 22446329 - 22460436

Genomic components: 4 coregulated genes, 8 genes

Chr	Strand	Start	End	RefSeq	Name	Exons	Description
chr3R	-	22446329	22447082	NM_143228	CG14240	1	CG14240-PA
chr3R	-	22448172	22449270	NM_143229	CG6478	2	CG6478-PA
chr3R	-	22450301	22451374	NM_170279	CG6447	2	CG6447-PA, isoform A
chr3R	-	22451731	22452419	NM_143230	CG6452	1	CG6452-PA
chr3R	-	22453687	22454429	NM_143231	CG6460	1	CG6460-PA
chr3R	+	22455578	22456500	NM_143232	CG5471	1	CG5471-PA
chr3R	+	22457594	22458678	NM_170281	CG5476	1	CG5476-PA
chr3R	+	22459455	22460436	NM_143233	CG31080	2	CG31080-PA

Cluster size: 14108 nucleotides

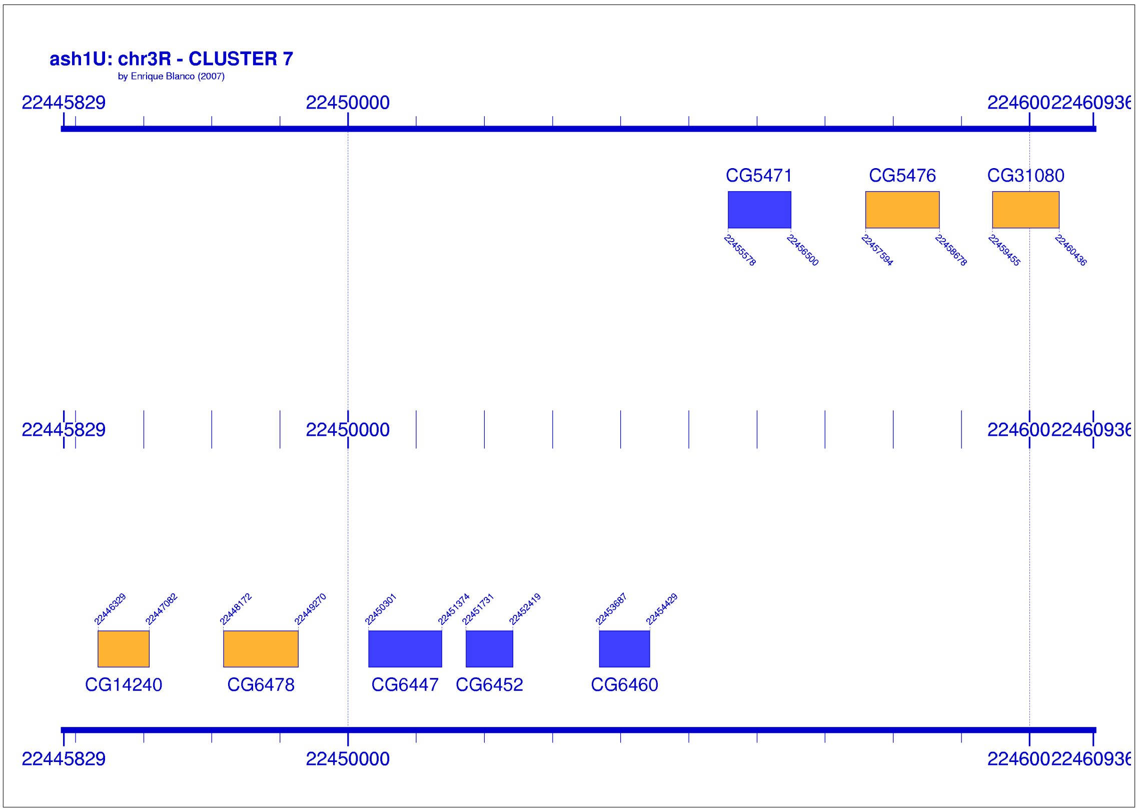

Enrique Blanco © 2007 — June 27, 2007

ash1D – chr3R: 2155971 - 2167150

Genomic components: 3 coregulated genes, 3 genes

CHR	Strand	Start	End	RefSeq	Name	Exons	Description
CHR3R	+	2155971	2157531	NM_141382	Osi18	2	Osiris 18 CG1169-PA
CHR3R	+	2161085	2162918	NM_141383	Osi19	4	Osiris 19 CG15189-PA
CHR3R	+	2165818	2167150	NM_141384	Osi20	2	Osiris 20 CG15188-PA

Cluster size: 11180 nucleotides

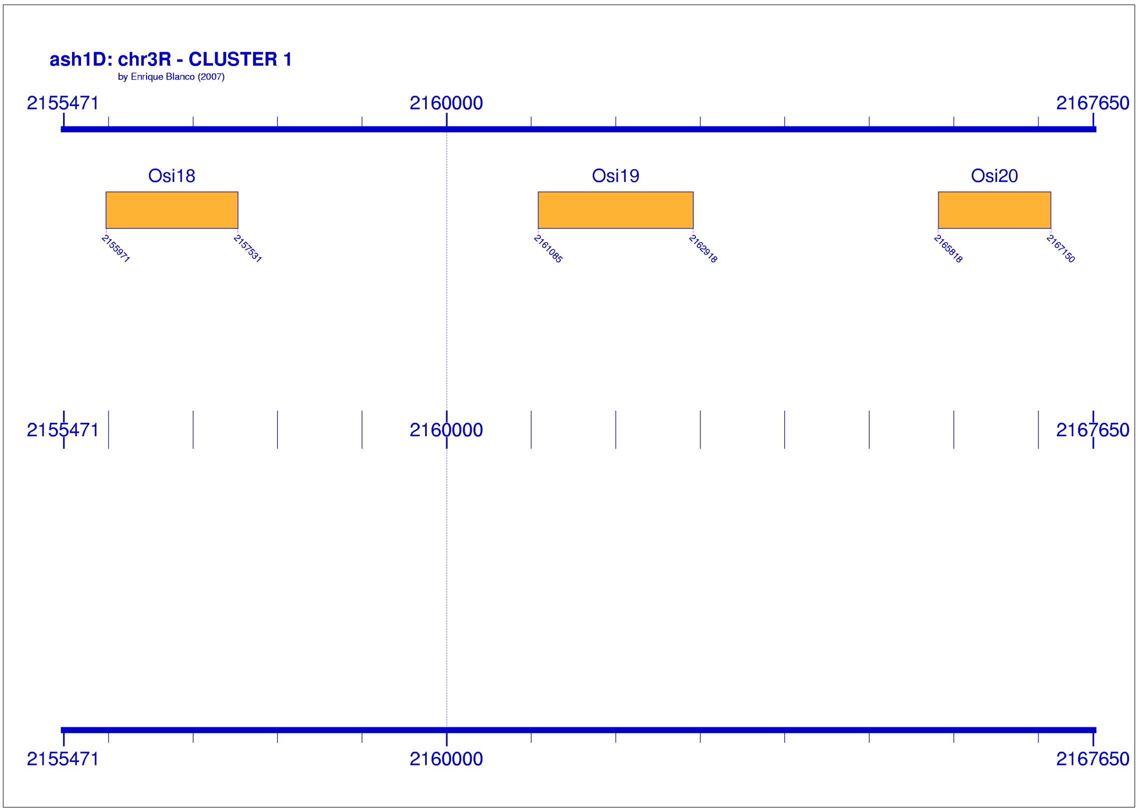

Enrique Blanco © 2007 — June 26, 2007
