# Supplementary material for: Conserved chromosomal clustering of genes governed by chromatin regulators in Drosophila
Source: Genome Biol. 2008 Sep 10;9(9):R134. doi: 10.1186/gb-2008-9-9-r134 (PMC2592712; doi:10.1186/gb-2008-9-9-r134)
Supplement: Additional data file 8 — Average lengths of the deregulated genes on each microarray (clustered or not). [file gb-2008-9-9-r134-S8.pdf]

Microarray	Avg. Size of all deregulated genes	Avg. Size of deregulated genes in Clusters	Avg. Size of deregulated genes Not in Clusters
Trithorax	2,965	946	3,416
ASH2	3,341	860	4,064
NURF	4,064	1,559	4,302
dMyc	3,343	930	3,711
ASH1	5,229	1,380	5,526
Total Avg.	3,788	1,135	4,204
