# Supplementary material for: Conserved chromosomal clustering of genes governed by chromatin regulators in Drosophila
Source: Genome Biol. 2008 Sep 10;9(9):R134. doi: 10.1186/gb-2008-9-9-r134 (PMC2592712; doi:10.1186/gb-2008-9-9-r134)
Supplement: Additional data file 9 — Clustering analysis in other microarrays of transcription factors. [file gb-2008-9-9-r134-S9.pdf]

TF	Genes ↑	Genes ↓	Clusters ↑	Clusters ↓	Reference
fkh	254	134	3	0	Insect Molecular Biology 17:91-101 (2008)
ey	254	-	0	-	Genome Research 16:466-476 (2006)
spdk	33	10	0	0	The EMBO Journal 24:4304-4315 (2005)
gcm	147	181	0	1	Developmental Biology 296:545-560 (2006)
Otd	287	-	0	-	Genome Biology 3:R15 (2002)
lab	48	48	0	0	Genome Biology 2:R15 (2001)
