# Supplementary material for: Conserved chromosomal clustering of genes governed by chromatin regulators in Drosophila
Source: Genome Biol. 2008 Sep 10;9(9):R134. doi: 10.1186/gb-2008-9-9-r134 (PMC2592712; doi:10.1186/gb-2008-9-9-r134)
Supplement: Additional data file 10 — Average gene size of deregulated genes in the microarrays analyzed in this study. [file gb-2008-9-9-r134-S10.pdf]

**Microarray**	**Avg. size of misexpressed genes**	**Clusters**	**Reference**	**Class**
**Rovers**	2,871	2	Riedl *et al.* (2005)	**CTRL**
**Trithorax**	2,965	25	-	**CHRE**
**ASH2**	3,341	8	-	**CHRE**
**dMyc**	3,343	6	Goodliffe *et al.* (2005)	**CHRE**
**Sitters**	3,425	3	Riedl *et al.* (2005)	**CTRL**
**Spotted-dick**	3,770	0	Page *et al.* (2005)	**SIXF**
**NURF**	4,064	7	Badenhorst *et al.* (2005)	**CHRE**
**Orthodenticle**	4,248	0	Montalta-He *et al.* (2002)	**SIXF**
**Larvae**	4,463	1	Arbeitman *et al.* (2002)	**CTRL**
**ASH1**	5,229	8	Goodliffe *et al.* (2007)	**CHRE**
**Gcm**	5,579	1	Althenheim *et al.* (2006)	**SIXF**
**Fork-head**	6,560	3	Liu *et al.* (2008)	**SIXF**
**Labial**	8,356	0	Leemans *et al.* (2001)	**SIXF**
**Eyeless**	9,985	0	Ostrin *et al.* (2006)	**SIXF**

CHRE: Chromatin remodeling factors
SIXF: Six TF (non-related to *trx*)
CTRL: Other microarrays (control)
