# Supplementary material for: Conserved chromosomal clustering of genes governed by chromatin regulators in Drosophila
Source: Genome Biol. 2008 Sep 10;9(9):R134. doi: 10.1186/gb-2008-9-9-r134 (PMC2592712; doi:10.1186/gb-2008-9-9-r134)
Supplement: Additional data file 12 — GO functional annotation of the trx clusters. [file gb-2008-9-9-r134-S12.pdf]

trxU – cluster 1

Genomic components:

NAME	RefSeq	Function	
LysB	NM_079158	GO:0003796	lysozyme activity
		GO:0005576	extracellular region
		GO:0006960	antimicrobial humoral response (sensu Protostomia)
		GO:0016998	cell wall catabolic process
LysC	NM_080130	GO:0003796	lysozyme activity
		GO:0005576	extracellular region
		GO:0006960	antimicrobial humoral response (sensu Protostomia)
		GO:0016998	cell wall catabolic process
LysD	NM_057475	GO:0003796	lysozyme activity
		GO:0004568	chitinase activity
		GO:0005576	extracellular region
		GO:0006960	antimicrobial humoral response (sensu Protostomia)
		GO:0016998	cell wall catabolic process
LysE	NM_057479	GO:0003796	lysozyme activity
		GO:0005576	extracellular region
		GO:0006960	antimicrobial humoral response (sensu Protostomia)
		GO:0016998	cell wall catabolic process
LysP	NM_057480	GO:0003796	lysozyme activity
		GO:0005576	extracellular region
		GO:0006960	antimicrobial humoral response (sensu Protostomia)
		GO:0016998	cell wall catabolic process
LysS	NM_057481	GO:0003796	lysozyme activity
		GO:0005576	extracellular region
		GO:0006960	antimicrobial humoral response (sensu Protostomia)
		GO:0016998	cell wall catabolic process

GO density (6 genes):

RANKING	GO id	Function	Frequency
1	GO:0005576	extracellular region	100 %
2	GO:0006960	antimicrobial humoral response (sensu Protostomia)	100 %
3	GO:0016998	cell wall catabolic process	100 %
4	GO:0003796	lysozyme activity	100 %
5	GO:0004568	chitinase activity	16 %

Enrique Blanco © 2007 — July 4, 2007

trxU – cluster 2

Genomic components:

NAME	RefSeq	Function	
HSP26	NM_079273	GO:0006457	protein folding
		GO:0006952	defense response
		GO:0008340	determination of adult life span
		GO:0009408	response to heat
HSP67BA	NM_079274	GO:0006457	protein folding
		GO:0006950	response to stress
		GO:0006952	defense response
		GO:0009408	response to heat
HSP23	NM_079275	GO:0003779	actin binding
		GO:0006457	protein folding
		GO:0006952	defense response
		GO:0009408	response to heat
HSP27	NM_079276	GO:0006457	protein folding
		GO:0006952	defense response
		GO:0008340	determination of adult life span
		GO:0009408	response to heat
		GO:0042026	protein refolding

GO density (4 genes):

RANKING	GO id	Function	Frequency
1	GO:0009408	response to heat	100 %
2	GO:0006952	defense response	100 %
3	GO:0006457	protein folding	100 %
4	GO:0008340	determination of adult life span	50 %
5	GO:0042026	protein refolding	25 %
6	GO:0006950	response to stress	25 %
7	GO:0003779	actin binding	25 %

Enrique Blanco © 2007 — July 4, 2007

trxU – cluster 3

Genomic components:

NAME	RefSeq	Function	
SGS8	NM_057371	GO:0005198	structural molecule activity
		GO:0005576	extracellular region
		GO:0007594	puparial adhesion
SGS7	NM_057370	GO:0005198	structural molecule activity
		GO:0005576	extracellular region
		GO:0007594	puparial adhesion
CG33272	NM_206323	GO:0003674	molecular_function
		GO:0005575	cellular_component
		GO:0008150	biological_process
SGS3	NM_079300	GO:0005198	structural molecule activity
		GO:0005576	extracellular region
		GO:0007594	puparial adhesion

GO density (4 genes):

RANKING	GO id	Function	Frequency
1	GO:0005198	structural molecule activity	75 %
2	GO:0005576	extracellular region	75 %
3	GO:0007594	puparial adhesion	75 %
4	GO:0008150	biological_process	25 %
5	GO:0003674	molecular_function	25 %
6	GO:0005575	cellular_component	25 %

Enrique Blanco © 2007 — July 4, 2007

trxU – cluster 4

Genomic components:

NAME	RefSeq	Function	
CG5883	NM_140269	GO:0005576	extracellular region
		GO:0006030	chitin metabolic process
		GO:0008061	chitin binding
		GO:0016490	structural constituent of peritrophic membrane
CG7252	NM_140270	GO:0005576	extracellular region
		GO:0006030	chitin metabolic process
		GO:0008061	chitin binding
		GO:0016490	structural constituent of peritrophic membrane
CG17826	NM_140271	GO:0005576	extracellular region
		GO:0006030	chitin metabolic process
		GO:0008061	chitin binding

GO density (3 genes):

RANKING	GO id	Function	Frequency
1	GO:0005576	extracellular region	100 %
2	GO:0006030	chitin metabolic process	100 %
3	GO:0008061	chitin binding	100 %
4	GO:0016490	structural constituent of peritrophic membrane	66 %

Enrique Blanco © 2007 — July 4, 2007

trxU – cluster 5

Genomic components:

NAME	RefSeq	Function
CG13461	NM_140481	
CG18649	NM_140482	
CG13463	NM_140483	
CG13460	NM_140484	

GO density (4 genes):

RANKING	GO id	Function	Frequency

Enrique Blanco © 2007 — July 4, 2007

trxU – cluster 6

Genomic components:

Name	RefSeq	Function	
CG7290	NM_140931	GO:0005576	extracellular region
		GO:0006030	chitin metabolic process
		GO:0008061	chitin binding
		GO:0016490	structural constituent of peritrophic membrane
CG6996	NM_140932	GO:0005576	extracellular region
		GO:0006030	chitin metabolic process
		GO:0008061	chitin binding
		GO:0016490	structural constituent of peritrophic membrane
CG32224	NM_168832		
CG7017	NM_140933	GO:0005576	extracellular region
		GO:0006030	chitin metabolic process
		GO:0008061	chitin binding
		GO:0016490	structural constituent of peritrophic membrane
CG6933	NM_140934	GO:0005576	extracellular region
		GO:0006030	chitin metabolic process
		GO:0008061	chitin binding
		GO:0016490	structural constituent of peritrophic membrane

GO density (5 genes):

Ranking	GO id	Function	Frequency
1	GO:0005576	extracellular region	80 %
2	GO:0016490	structural constituent of peritrophic membrane	80 %
3	GO:0006030	chitin metabolic process	80 %
4	GO:0008061	chitin binding	80 %

Enrique Blanco © 2007 — July 4, 2007

trxU – cluster 7

Genomic components:

NAME	RefSeq	Function	
CG9080	NM_136806		
CG13226	NM_136807		
CG30029	NM_165812		
CG7738	NM_136809		
OR47A	NM_078965	GO:0004984	olfactory receptor activity
		GO:0005549	odorant binding
		GO:0007608	sensory perception of smell
		GO:0016021	integral to membrane
CG9079	NM_136810	GO:0005214	structural constituent of chitin-based cuticle
CG13224	NM_136811	GO:0008010	structural constituent of chitin-based larval cuticle

GO density (7 genes):

RANKING	GO id	Function	Frequency
1	GO:0008010	structural constituent of chitin-based larval cuticle	14 %
2	GO:0005214	structural constituent of chitin-based cuticle	14 %
3	GO:0004984	olfactory receptor activity	14 %
4	GO:0005549	odorant binding	14 %
5	GO:0016021	integral to membrane	14 %
6	GO:0007608	sensory perception of smell	14 %

Enrique Blanco © 2007 — July 4, 2007

trxU – cluster 8

Genomic components:

NAME	RefSeq	Function	
CG18107	NM_145336		
CG15067	NM_137476		
IM2	NM_166277	GO:0005576	extracellular region
		GO:0006952	defense response
IM3	NM_144111	GO:0003674	molecular_function
		GO:0005576	extracellular region
		GO:0006952	defense response
		GO:0008063	Toll signaling pathway
		GO:0019731	antibacterial humoral response
CG16836	NM_144110		
CG15065	NM_144109		
CG15068	NM_176222		

GO density (7 genes):

RANKING	GO id	Function	Frequency
1	GO:0005576	extracellular region	28 %
2	GO:0006952	defense response	28 %
3	GO:0019731	antibacterial humoral response	14 %
4	GO:0008063	Toll signaling pathway	14 %
5	GO:0003674	molecular_function	14 %

Enrique Blanco © 2007 — July 4, 2007

trxU – cluster 9

Genomic components:

NAME	RefSeq	Function
CG14850	NM_142114	
CG14851	NM_142115	
CG8087	NM_169585	
CG14852	NM_142116	

GO density (4 genes):

RANKING	GO id	Function	Frequency

Enrique Blanco © 2007 — July 4, 2007

trxU – cluster 10

Genomic components:

NAME	RefSeq	Function
CG7714	NM_142490	
CG7715	NM_142491	
CG14302	NM_142492	

GO density (3 genes):

RANKING	GO id	Function	Frequency

Enrique Blanco © 2007 — July 4, 2007

trxD – cluster 1

Genomic components:

NAME	RefSeq	Function	
ACP1	NM_057767	GO:0008012	structural constituent of adult chitin-based cuticle
CG7214	NM_135297		
CG7211	NM_135298	GO:0005753	mitochondrial proton-transporting ATP synthase complex
		GO:0006118	electron transport
		GO:0006119	oxidative phosphorylation
		GO:0006812	cation transport
		GO:0008553	hydrogen-exporting ATPase activity, phosphorylative mechanism
		GO:0015986	ATP synthesis coupled proton transport
		GO:0046933	hydrogen ion transporting ATP synthase activity, rotational mechanism
		GO:0046961	hydrogen ion transporting ATPase activity, rotational mechanism
CG7203	NM_135299		

GO density (4 genes):

RANKING	GO id	Function	Frequency
1	GO:0046961	hydrogen ion transporting ATPase activity, rotational mechanism	25 %
2	GO:0046933	hydrogen ion transporting ATP synthase activity, rotational mechanism	25 %
3	GO:0008553	hydrogen-exporting ATPase activity, phosphorylative mechanism	25 %
4	GO:0015986	ATP synthesis coupled proton transport	25 %
5	GO:0006119	oxidative phosphorylation	25 %
6	GO:0006118	electron transport	25 %
7	GO:0006812	cation transport	25 %
8	GO:0005753	mitochondrial proton-transporting ATP synthase complex	25 %
9	GO:0008012	structural constituent of adult chitin-based cuticle	25 %

Enrique Blanco © 2007 — July 4, 2007

trxD – cluster 2

Genomic components:

Name	RefSeq	Function	
CG9149	NM_138250	GO:0003985	acetyl-CoA C-acetyltransferase activity
		GO:0005829	cytosol
CG2277	NM_138251	GO:0006139	nucleobase, nucleoside, nucleotide and nucleic acid metabolic process
		GO:0006144	purine base metabolic process
		GO:0019204	nucleotide phosphatase activity
CG2469	NM_176271	GO:0005488	binding
CG9186	NM_138253		

GO density (4 genes):

Ranking	GO id	Function	Frequency
1	GO:0003985	acetyl-CoA C-acetyltransferase activity	25 %
2	GO:0006144	purine base metabolic process	25 %
3	GO:0005829	cytosol	25 %
4	GO:0019204	nucleotide phosphatase activity	25 %
5	GO:0005488	binding	25 %
6	GO:0006139	nucleobase, nucleoside, nucleotide and nucleic acid metabolic process	25 %

Enrique Blanco © 2007 — July 4, 2007

trxD – cluster 3

Genomic components:

NAME	RefSeq	Function	
CG12607	NM_139646		
CG11345	NM_139647		
CG15022	NM_139648		
CG15023	NM_139649		
CG15024	NM_144079		
CG32241	NM_168087	GO:0003674<br>GO:0005575<br>GO:0008150	molecular_function<br>cellular_component<br>biological_process

GO density (6 genes):

RANKING	GO id	Function	Frequency
1	GO:0008150	biological_process	16 %
2	GO:0003674	molecular_function	16 %
3	GO:0005575	cellular_component	16 %

Enrique Blanco © 2007 — July 4, 2007

trxD – cluster 4

Genomic components:

NAME	RefSeq	Function	
L(3)MBN	NM_001014570	GO:0005737	cytoplasm
		GO:0005886	plasma membrane
		GO:0042302	structural constituent of cuticle
		GO:0042387	plasmatocyte differentiation
CG18779	NM_144405		
CG18778	NM_144404		
LCP65AG2	NM_057924	GO:0008010	structural constituent of chitin-based larval cuticle
LCP65AG1	NM_057925	GO:0005576	extracellular region
		GO:0008010	structural constituent of chitin-based larval cuticle
		GO:0008363	larval chitin-based cuticle development
LCP65AF	NM_057926	GO:0008010	structural constituent of chitin-based larval cuticle
LCP65AE	NM_176290	GO:0008010	structural constituent of chitin-based larval cuticle
CG32405	NM_168158	GO:0005214	structural constituent of chitin-based cuticle
CG32404	NM_168159	GO:0005214	structural constituent of chitin-based cuticle
LCP65AD	NM_057930	GO:0008010	structural constituent of chitin-based larval cuticle
LCP65AC	NM_057931	GO:0008010	structural constituent of chitin-based larval cuticle
LCP65AB2	NM_176291	GO:0008010	structural constituent of chitin-based larval cuticle
LCP65AB1	NM_080075	GO:0005576	extracellular region
		GO:0008010	structural constituent of chitin-based larval cuticle
		GO:0008363	larval chitin-based cuticle development
CG18777	NM_144403		
LCP65AA	NM_057932	GO:0008010	structural constituent of chitin-based larval cuticle

GO density (15 genes):

RANKING	GO id	Function	Frequency
1	GO:0008010	structural constituent of chitin-based larval cuticle	60 %
2	GO:0008363	larval chitin-based cuticle development	13 %
3	GO:0005576	extracellular region	13 %
4	GO:0005214	structural constituent of chitin-based cuticle	13 %
5	GO:0005886	plasma membrane	6 %
6	GO:0042387	plasmatocyte differentiation	6 %
7	GO:0005737	cytoplasm	6 %
8	GO:0042302	structural constituent of cuticle	6 %

Enrique Blanco © 2007 — July 4, 2007

trxD – cluster 5

Genomic components:

NAME	RefSeq	Function
CG8012	NM_144191	
CG13674	NM_139935	
CG13678	NM_139936	

GO density (3 genes):

RANKING	GO id	Function	Frequency

Enrique Blanco © 2007 — July 4, 2007

trxD – cluster 6

Genomic components:

NAME	RefSeq	Function	
CG7628	NM_140184	GO:0005315	inorganic phosphate transporter activity
		GO:0006796	phosphate metabolic process
		GO:0006817	phosphate transport
		GO:0015114	phosphate transporter activity
		GO:0016020	membrane
NOL	NM_080275	GO:0005576	extracellular region
		GO:0007405	neuroblast proliferation
CG32074	NM_168447	GO:0003674	molecular_function
		GO:0005575	cellular_component
		GO:0008150	biological_process
CG14143	NM_140185		

GO density (4 genes):

RANKING	GO id	Function	Frequency
1	GO:0006796	phosphate metabolic process	25 %
2	GO:0008150	biological_process	25 %
3	GO:0005576	extracellular region	25 %
4	GO:0005315	inorganic phosphate transporter activity	25 %
5	GO:0006817	phosphate transport	25 %
6	GO:0007405	neuroblast proliferation	25 %
7	GO:0003674	molecular_function	25 %
8	GO:0015114	phosphate transporter activity	25 %
9	GO:0016020	membrane	25 %
10	GO:0005575	cellular_component	25 %

Enrique Blanco © 2007 — July 4, 2007

trxD – cluster 7

Genomic components:

Name	RefSeq	Function	
CG13069	NM_144161		
CG4950	NM_140598	GO:0004872	receptor activity
		GO:0006952	defense response
		GO:0007155	cell adhesion
		GO:0007165	signal transduction
		GO:0007166	cell surface receptor linked signal transduction
		GO:0019221	cytokine and chemokine mediated signaling pathway
		GO:0019226	transmission of nerve impulse
CG13068	NM_140599		
CG13067	NM_206383		
CG13066	NM_144160		
CG13065	NM_140601		
CG13050	NM_140602		
CG13064	NM_206384		
CG13049	NM_140603		
CG13048	NM_140604		
CG13047	NM_140605		
CG13046	NM_140606		
CG13045	NM_140607		
CG4962	NM_140608		

GO density (14 genes):

Ranking	GO id	Function	Frequency
1	GO:0019221	cytokine and chemokine mediated signaling pathway	7 %
2	GO:0006952	defense response	7 %
3	GO:0007155	cell adhesion	7 %
4	GO:0007166	cell surface receptor linked signal transduction	7 %
5	GO:0004872	receptor activity	7 %
6	GO:0019226	transmission of nerve impulse	7 %
7	GO:0007165	signal transduction	7 %

Enrique Blanco © 2007 — July 4, 2007

trxD – cluster 8

Genomic components:

NAME	RefSeq	Function	
CG4982	NM_140609		
CG13044	NM_140610		
CG13043	NM_140611		
CG13063	NM_140612		
CG13042	NM_140613		
CG32160	NM_168658	GO:0003674<br>GO:0005575<br>GO:0008150	molecular_function<br>cellular_component<br>biological_process
CG13062	NM_140614		
NPLP3	NM_144453	GO:0005184<br>GO:0005576<br>GO:0007218	neuropeptide hormone activity<br>extracellular region<br>neuropeptide signaling pathway
CG13041	NM_140615		
CG13060	NM_140616		
CG13059	NM_140617		

GO density (11 genes):

RANKING	GO id	Function	Frequency
1	GO:0008150	biological_process	9 %
2	GO:0007218	neuropeptide signaling pathway	9 %
3	GO:0005576	extracellular region	9 %
4	GO:0003674	molecular_function	9 %
5	GO:0005184	neuropeptide hormone activity	9 %
6	GO:0005575	cellular_component	9 %

Enrique Blanco © 2007 — July 4, 2007

trxD – cluster 9

Genomic components:

NAME	RefSeq	Function	
CG11310	NM_141041	GO:0042302	structural constituent of cuticle
CG7663	NM_141042	GO:0042302	structural constituent of cuticle
EDG78E	NM_079474	GO:0008011	structural constituent of pupal chitin-based cuticle
CG7658	NM_141043	GO:0008010	structural constituent of chitin-based larval cuticle

GO density (4 genes):

RANKING	GO id	Function	Frequency
1	GO:0042302	structural constituent of cuticle	50 %
2	GO:0008010	structural constituent of chitin-based larval cuticle	25 %
3	GO:0008011	structural constituent of pupal chitin-based cuticle	25 %

Enrique Blanco © 2007 — July 4, 2007

trxD – cluster 10

Genomic components:

NAME	RefSeq	Function	
CG14569	NM_141097		
CG14568	NM_141098		
CG14573	NM_141099		
CG14567	NM_141100		
CG14566	NM_141101		
CG14572	NM_141102		
CG14565	NM_141103		
SYN1	NM_168937	GO:0005200	structural constituent of cytoskeleton
		GO:0005215	transporter activity
		GO:0006810	transport
		GO:0006936	muscle contraction
		GO:0007165	signal transduction
		GO:0007268	synaptic transmission
		GO:0008092	cytoskeletal protein binding
		GO:0008307	structural constituent of muscle
		GO:0016013	syntrophin complex
CG14564	NM_141104		

GO density (9 genes):

RANKING	GO id	Function	Frequency
1	GO:0005215	transporter activity	11 %
2	GO:0016013	syntrophin complex	11 %
3	GO:0008307	structural constituent of muscle	11 %
4	GO:0007268	synaptic transmission	11 %
5	GO:0005200	structural constituent of cytoskeleton	11 %
6	GO:0006936	muscle contraction	11 %
7	GO:0008092	cytoskeletal protein binding	11 %
8	GO:0007165	signal transduction	11 %
9	GO:0006810	transport	11 %

Enrique Blanco © 2007 — July 4, 2007

trxD – cluster 11

Genomic components:

Name	RefSeq	Function	
CG8836	NM_136929	GO:0008010	structural constituent of chitin-based larval cuticle
Or49a	NM_078987	GO:0004984	olfactory receptor activity
		GO:0005549	odorant binding
		GO:0007608	sensory perception of smell
		GO:0016021	integral to membrane
		GO:0042048	olfactory behavior
CG30048	NM_165895	GO:0003674	molecular_function
		GO:0005575	cellular_component
		GO:0008150	biological_process
CG8505	NM_136930	GO:0005214	structural constituent of chitin-based cuticle
CG8510	NM_136931	GO:0042302	structural constituent of cuticle
CG8511	NM_136932	GO:0005214	structural constituent of chitin-based cuticle
CG30050	NM_165897	GO:0003674	molecular_function
		GO:0005575	cellular_component
		GO:0008150	biological_process
CG33626	NM_001032234	GO:0003674	molecular_function
		GO:0005575	cellular_component
		GO:0008150	biological_process
CG33627	NM_001032235	GO:0003674	molecular_function
		GO:0005575	cellular_component
		GO:0008150	biological_process
CG8515	NM_136933	GO:0005214	structural constituent of chitin-based cuticle
CG13157	NM_136934		
CG8834	NM_136935	GO:0008152	metabolic process
		GO:0016207	4-coumarate-CoA ligase activity
CG8520	NM_136936	GO:0016887	ATPase activity

GO density (13 genes):

Ranking	GO id	Function	Frequency
1	GO:0008150	biological_process	30 %
2	GO:0003674	molecular_function	30 %
3	GO:0005575	cellular_component	30 %
4	GO:0005214	structural constituent of chitin-based cuticle	23 %
5	GO:0016887	ATPase activity	7 %
6	GO:0008010	structural constituent of chitin-based larval cuticle	7 %
7	GO:0016021	integral to membrane	7 %
8	GO:0042048	olfactory behavior	7 %
9	GO:0016207	4-coumarate-CoA ligase activity	7 %
10	GO:0004984	olfactory receptor activity	7 %
11	GO:0005549	odorant binding	7 %
12	GO:0008152	metabolic process	7 %
13	GO:0007608	sensory perception of smell	7 %
14	GO:0042302	structural constituent of cuticle	7 %

Enrique Blanco © 2007 — July 4, 2007

trxD – cluster 12

Genomic components:

NAME	RefSeq	Function
CG30458	NM_166220	
CG30457	NM_137354	
CG10953	NM_137355	

GO density (3 genes):

RANKING	GO id	Function	Frequency

Enrique Blanco © 2007 — July 4, 2007

trxD – cluster 13

Genomic components:

Name	RefSeq	Function	
Ccp84Ag	NM_141420	GO:0008010	structural constituent of chitin-based larval cuticle
Ccp84Af	NM_141421	GO:0008010	structural constituent of chitin-based larval cuticle
Ccp84Ae	NM_141422	GO:0008010	structural constituent of chitin-based larval cuticle
Ccp84Ad	NM_141423	GO:0008010	structural constituent of chitin-based larval cuticle
Ccp84Ac	NM_141424	GO:0008010	structural constituent of chitin-based larval cuticle
Ccp84Ab	NM_141425	GO:0008010	structural constituent of chitin-based larval cuticle
Ccp84Aa	NM_141426	GO:0008010	structural constituent of chitin-based larval cuticle

GO density (7 genes):

Ranking	GO id	Function	Frequency
1	GO:0008010	structural constituent of chitin-based larval cuticle	100 %

Enrique Blanco © 2007 — July 4, 2007

trxD – cluster 14

Genomic components:

NAME	RefSeq	Function
CG5468	NM_143227	
CG14240	NM_143228	
CG6478	NM_143229	
CG6447	NM_170279	
CG6452	NM_143230	
CG6460	NM_143231	
CG5471	NM_143232	
CG5476	NM_170281	

GO density (8 genes):

RANKING	GO id	Function	Frequency

Enrique Blanco © 2007 — July 4, 2007

trxD – cluster 15

Genomic components:

Name	RefSeq	Function	
CG32564	NM_167559		
CG32563	NM_167560		
CG12995	NM_132972		
CG18258	NM_132973	GO:0006629	lipid metabolic process
		GO:0006644	phospholipid metabolic process
		GO:0007275	multicellular organismal development
		GO:0007276	gamete generation
		GO:0007292	female gamete generation
		GO:0016298	lipase activity
CG5162	NM_132974	GO:0005198	structural molecule activity
		GO:0006644	phospholipid metabolic process
		GO:0007292	female gamete generation
		GO:0016298	lipase activity
CG12998	NM_132975		
CG5172	NM_132976		
CG12997	NM_132977		
CG10598	NM_132978		
CG10597	NM_132979		

GO density (10 genes):

Ranking	GO id	Function	Frequency
1	GO:0016298	lipase activity	20 %
2	GO:0006644	phospholipid metabolic process	20 %
3	GO:0007292	female gamete generation	20 %
4	GO:0006629	lipid metabolic process	10 %
5	GO:0007276	gamete generation	10 %
6	GO:0005198	structural molecule activity	10 %
7	GO:0007275	multicellular organismal development	10 %

Enrique Blanco © 2007 — July 4, 2007
