# Supplementary material for: Conserved chromosomal clustering of genes governed by chromatin regulators in Drosophila
Source: Genome Biol. 2008 Sep 10;9(9):R134. doi: 10.1186/gb-2008-9-9-r134 (PMC2592712; doi:10.1186/gb-2008-9-9-r134)
Supplement: Additional data file 13 — Clusters of genes in the genome associated with similar categories (cuticle/chitin binding). [file gb-2008-9-9-r134-S13.pdf]

cuticle – chr3L: 1807953 - 1825612

Genomic components: 3 coregulated genes, 3 genes

CHR	Strand	Start	End	RefSeq	Name	Exons	Description
CHR3L	-	1807953	1808733	NM_139409	CG13934	2	CG13934-PA
CHR3L	-	1816167	1818693	NM_139410	CG13935	5	CG13935-PA
CHR3L	-	1823811	1825612	NM_139411	CG1919	3	CG1919-PA

Cluster size: 17660 nucleotides

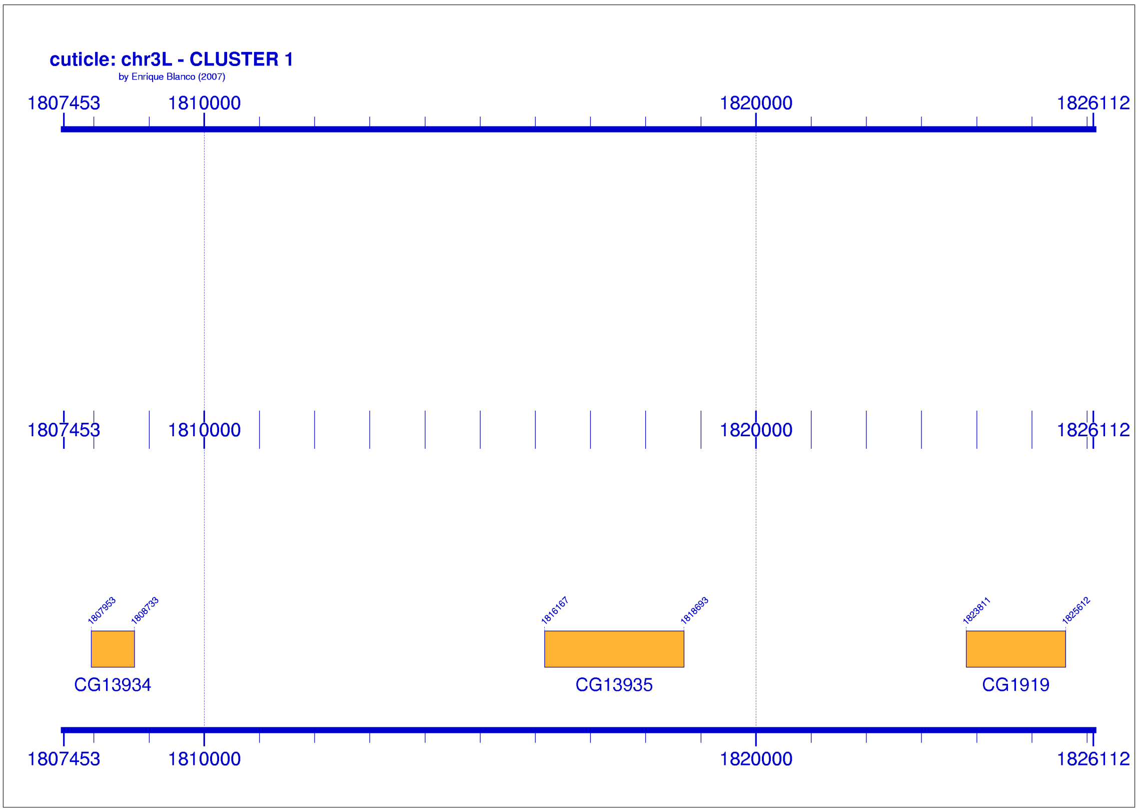

Enrique Blanco © 2007 — July 4, 2007

cuticle – chr3L: 4191367 - 4199946

Genomic components: 4 coregulated genes, 4 genes

CHR	Strand	Start	End	RefSeq	Name	Exons	Description
CHR3L	-	4191367	4192718	NM_139615	CG15006	2	CG15006-PA
CHR3L	-	4194534	4195085	NM_139616	CG15007	2	CG15007-PA
CHR3L	-	4195776	4196405	NM_139617	CG15008	2	CG15008-PA
CHR3L	+	4198915	4199946	NM_139618	CG1259	2	CG1259-PB

Cluster size: 8580 nucleotides

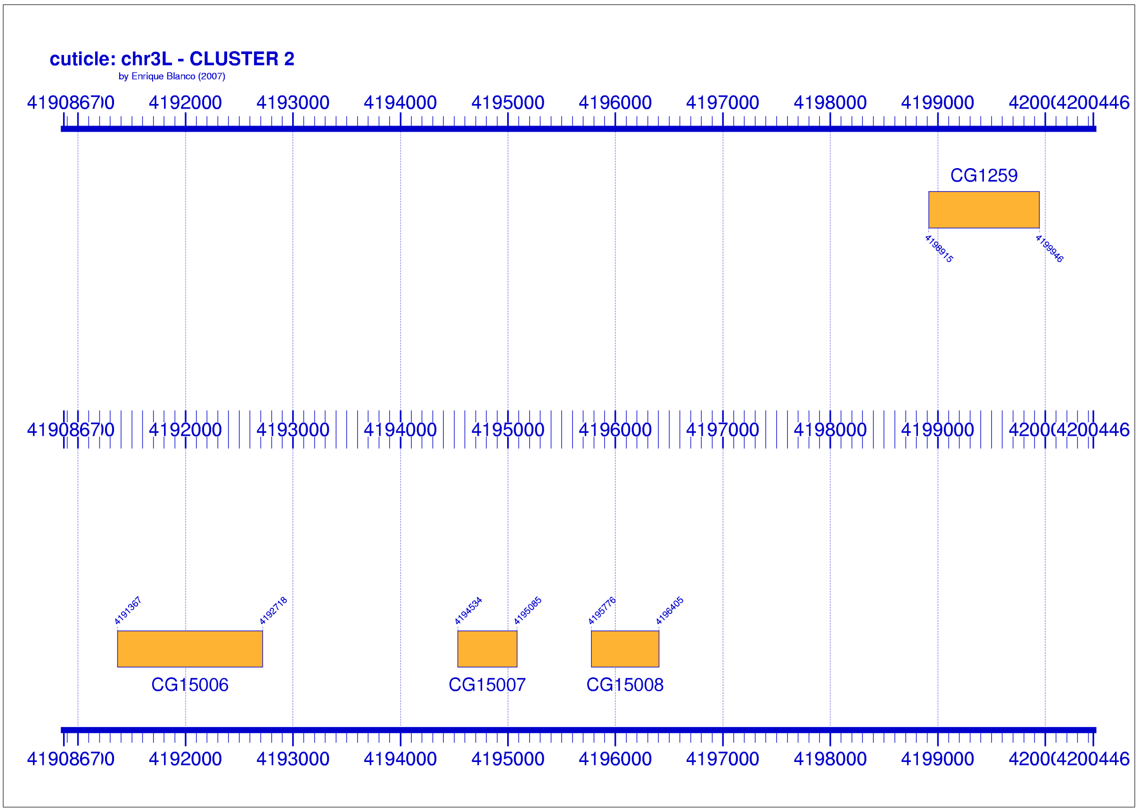

Enrique Blanco © 2007 — July 4, 2007

cuticle – chr3L: 6097832 - 6135534

Genomic components: 14 coregulated genes, 18 genes

CHR	Strand	Start	End	RefSeq	Name	Exons	Description
CHR3L	-	6097832	6102566	NM_001014570	l(3)mbn	7	lethal (3) malignant blood neoplasm CG12755-PC,
CHR3L	-	6104332	6104933	NM_144405	CG18779	2	CG18779-PA
CHR3L	-	6105259	6106056	NM_144404	CG18778	2	CG18778-PA
CHR3L	-	6106936	6107547	NM_057924	Lcp65Ag2	2	Lcp65Ag2 CG10534-PA
CHR3L	-	6108643	6109217	NM_057925	Lcp65Ag1	2	Lcp65Ag1 CG10530-PA
CHR3L	-	6110251	6110718	NM_057926	Lcp65Af	2	Lcp65Af CG10533-PA
CHR3L	-	6111667	6112025	NM_176290	Lcp65Ae	2	Lcp65Ae CG10529-PA
CHR3L	-	6113169	6113920	NM_168158	CG32405	2	CG32405-PA
CHR3L	-	6115912	6116330	NM_168159	CG32404	2	CG32404-PA
CHR3L	+	6117353	6117953	NM_057930	Lcp65Ad	2	Lcp65Ad CG6955-PA
CHR3L	+	6118770	6119345	NM_057931	Lcp65Ac	2	Lcp65Ac CG6956-PA
CHR3L	+	6120617	6121036	NM_176291	Lcp65Ab2	1	Lcp65Ab2 CG18773-PA
CHR3L	+	6120668	6120981	NM_080075	Lcp65Ab1	1	Lcp65Ab1 CG32400-PA
CHR3L	-	6121477	6121928	NM_144403	CG18777	2	CG18777-PA
CHR3L	+	6125279	6125586	NM_057932	Lcp65Aa	1	Lcp65Aa CG7287-PA
CHR3L	-	6125994	6126692	NM_057934	Acp65Aa	2	Acp65Aa CG10297-PA
CHR3L	-	6130541	6131320	NM_139773	CG13297	2	CG13297-PA
CHR3L	+	6134760	6135534	NM_139774	CG12330	2	CG12330-PA

Cluster size: 37703 nucleotides

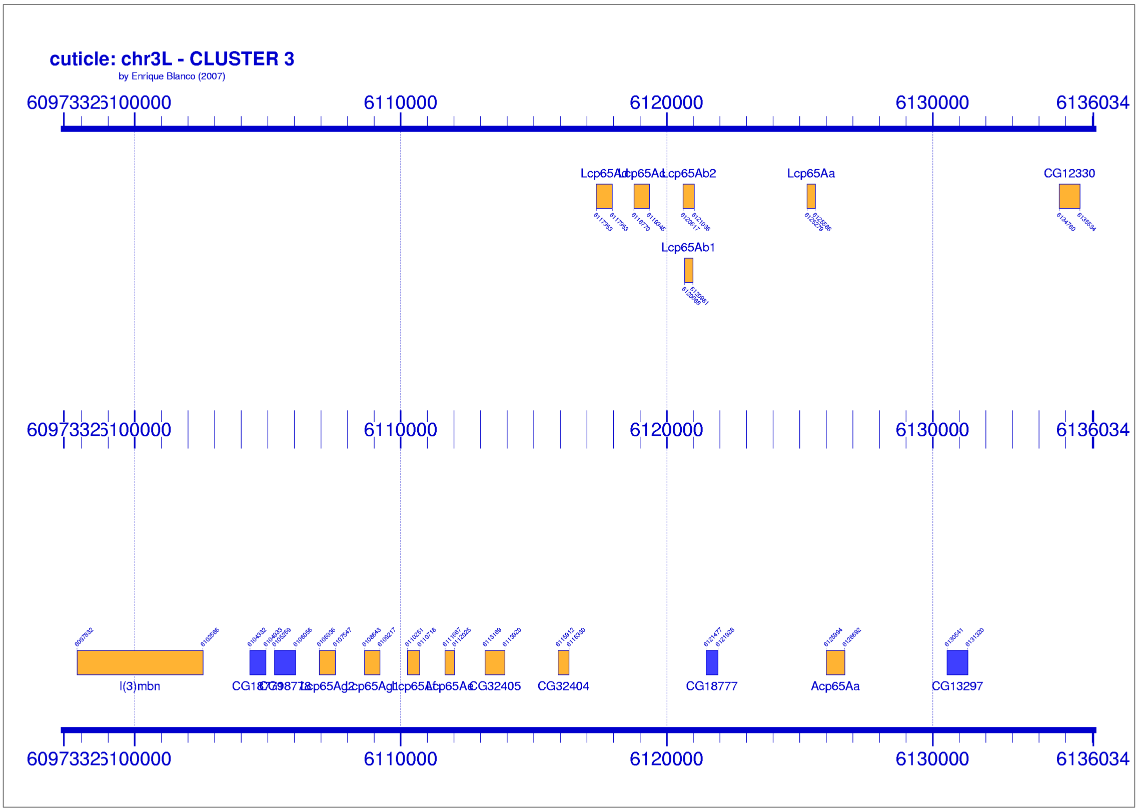

Enrique Blanco © 2007 — July 4, 2007

cuticle – chr3L: 7060065 - 7067788

Genomic components: 3 coregulated genes, 3 genes

Chr	Strand	Start	End	RefSeq	Name	Exons	Description
chr3L	-	7060065	7060515	NM_139818	CG8640	2	CG8640-PA
chr3L	-	7061653	7062732	NM_139819	CG8638	2	CG8638-PA
chr3L	+	7067155	7067788	NM_139820	CG8634	2	CG8634-PA

Cluster size: 7724 nucleotides

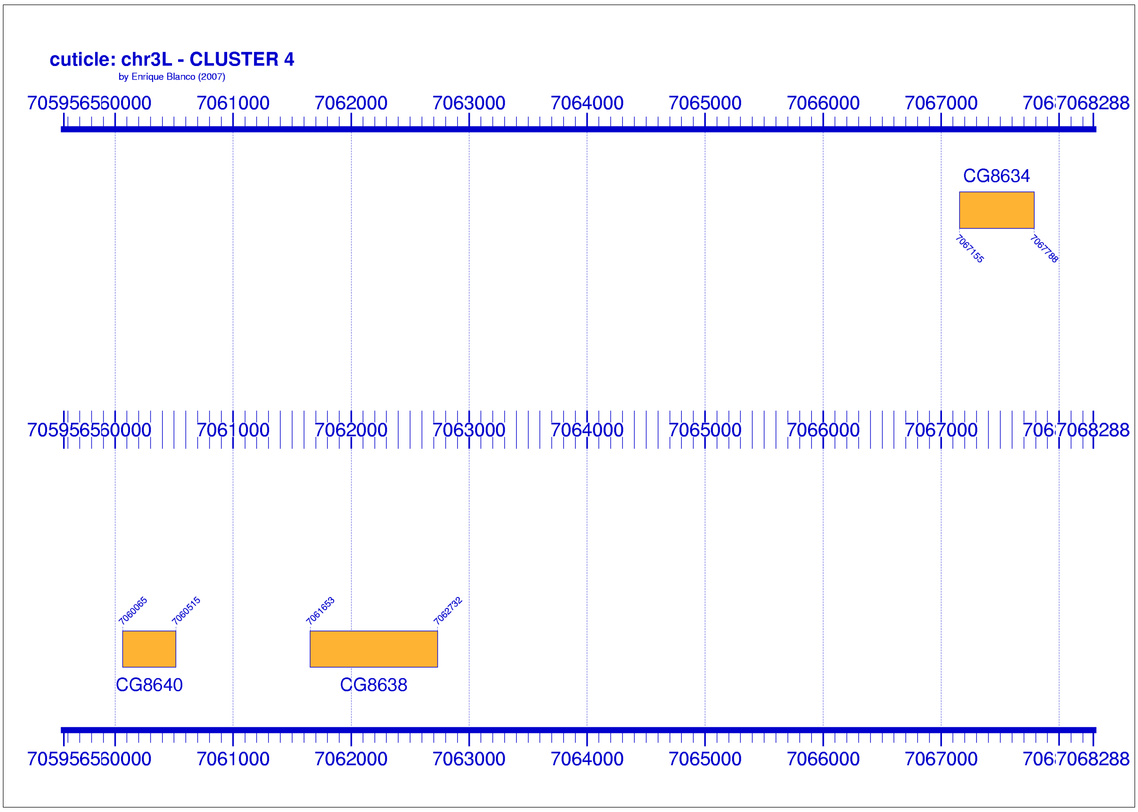

Enrique Blanco © 2007 — July 4, 2007

cuticle – chr3L: 8294782 - 8312093

Genomic components: 3 coregulated genes, 3 genes

CHR	Strand	Start	End	RefSeq	Name	Exons	Description
CHR3L	+	8294782	8296141	NM_139950	CG13670	2	CG13670-PA
CHR3L	+	8299602	8304061	NM_139951	CG7072	4	CG7072-PA
CHR3L	+	8310683	8312093	NM_139952	CG7076	3	CG7076-PA

Cluster size: 17312 nucleotides

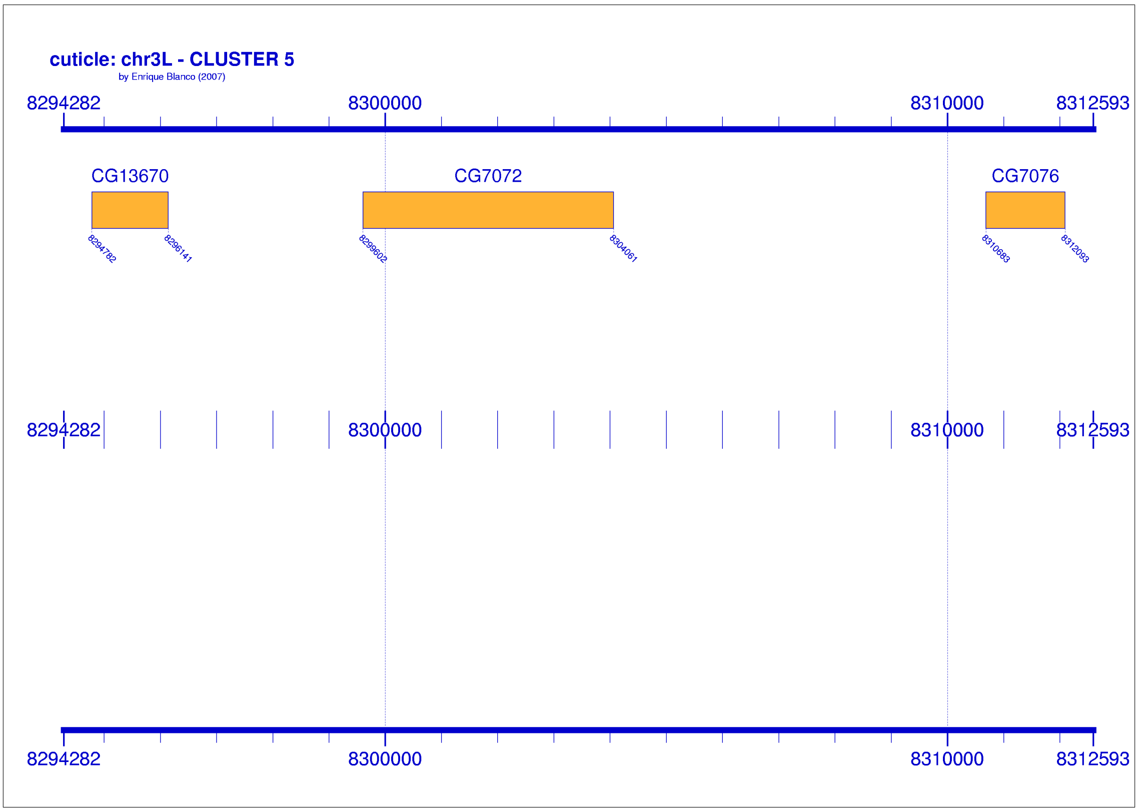

Enrique Blanco © 2007 — July 4, 2007

cuticle – chr3L: 16327861 - 16332585

Genomic components: 3 coregulated genes, 3 genes

CHR	Strand	Start	End	RefSeq	Name	Exons	Description
CHR3L	+	16327861	16328939	NM_140625	CG4818	1	CG4818-PA
CHR3L	+	16330255	16330907	NM_140626	CG12255	1	CG12255-PA
CHR3L	-	16331152	16332585	NM_140627	CG4784	1	CG4784-PA

Cluster size: 4725 nucleotides

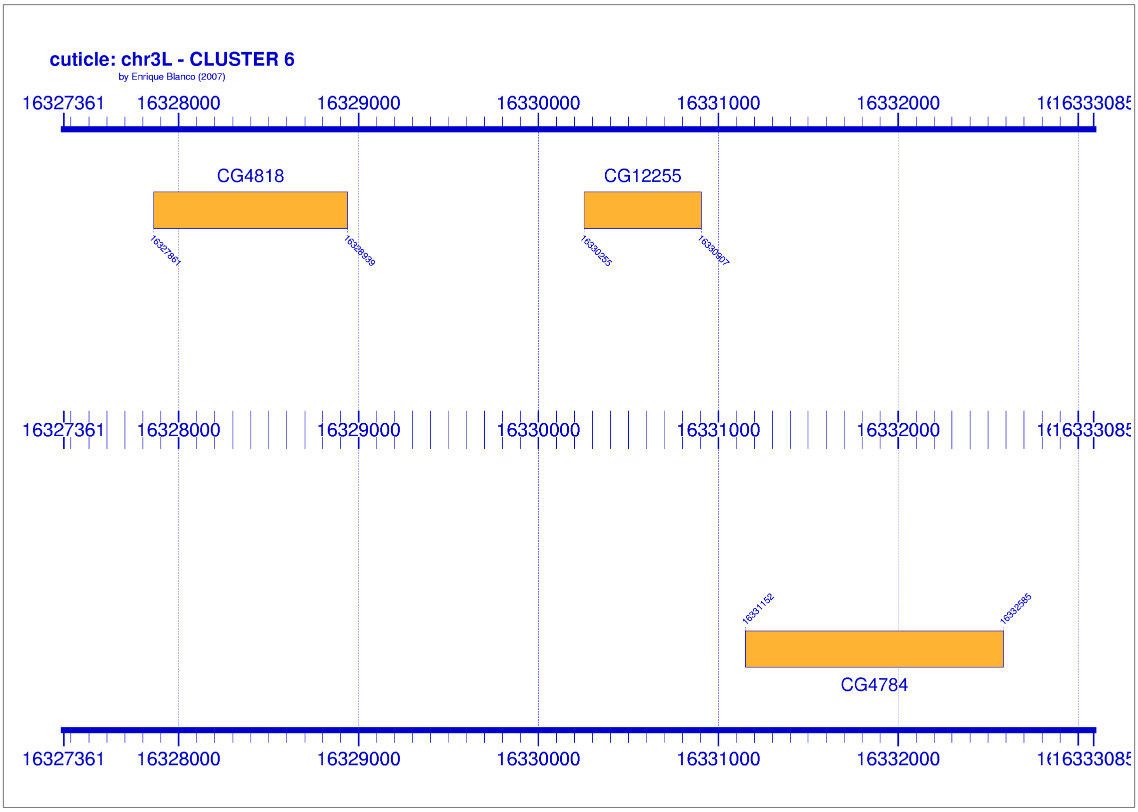

Enrique Blanco © 2007 — July 4, 2007

cuticle – chr3L: 19482919 - 19502671

Genomic components: 4 coregulated genes, 4 genes

CHR	Strand	Start	End	RefSeq	Name	Exons	Description
CHR3L	-	19482919	19483534	NM_140863	CG9283	1	CG9283-PA
CHR3L	+	19484953	19485548	NM_140864	CG9290	1	CG9290-PA
CHR3L	+	19490729	19492403	NM_140865	CG9295	2	CG9295-PB
CHR3L	+	19498464	19502671	NM_140866	CG9299	4	CG9299-PA

Cluster size: 19753 nucleotides

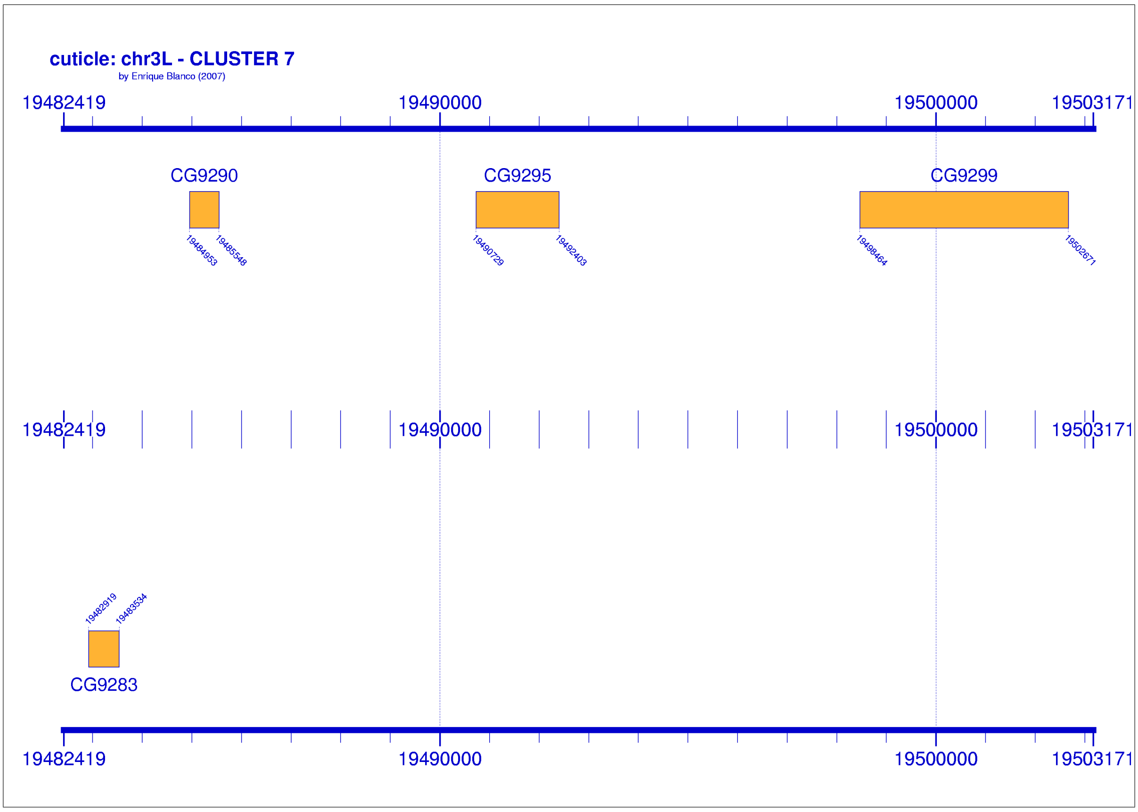

Enrique Blanco © 2007 — July 4, 2007

cuticle – chr3L: 21226060 - 21235012

Genomic components: 4 coregulated genes, 4 genes

CHR	Strand	Start	End	RefSeq	Name	Exons	Description
CHR3L	-	21226060	21226508	NM_141041	CG11310	2	CG11310-PA
CHR3L	+	21229241	21230539	NM_141042	CG7663	2	CG7663-PA
CHR3L	-	21230603	21231644	NM_079474	Edg78E	2	Ecdysone-dependent gene 78E CG7673-PA
CHR3L	+	21234428	21235012	NM_141043	CG7658	2	CG7658-PA

Cluster size: 8953 nucleotides

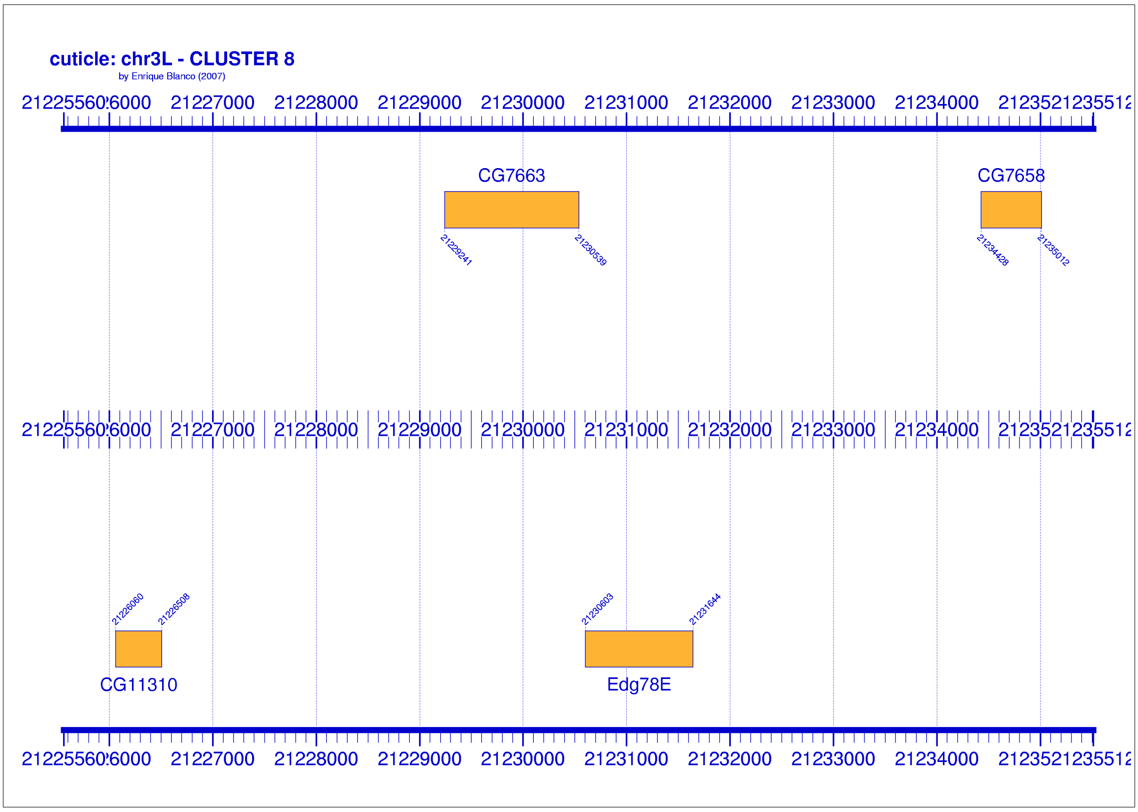

Enrique Blanco © 2007 — July 4, 2007

cuticle – chr2R: 3942446 - 3950215

Genomic components: 4 coregulated genes, 4 genes

CHR	Strand	Start	End	RefSeq	Name	Exons	Description
CHR2R	-	3942446	3943045	NM_057271	Lcp1	2	Larval cuticle protein 1 CG11650-PA
CHR2R	-	3945779	3946388	NM_057272	Lcp2	2	Larval cuticle protein 2 CG8697-PA
CHR2R	+	3947256	3947829	NM_057273	Lcp3	2	Larval cuticle protein 3 CG2043-PA
CHR2R	+	3949469	3950215	NM_057274	Lcp4	2	Larval cuticle protein 4 CG2044-PA

Cluster size: 7770 nucleotides

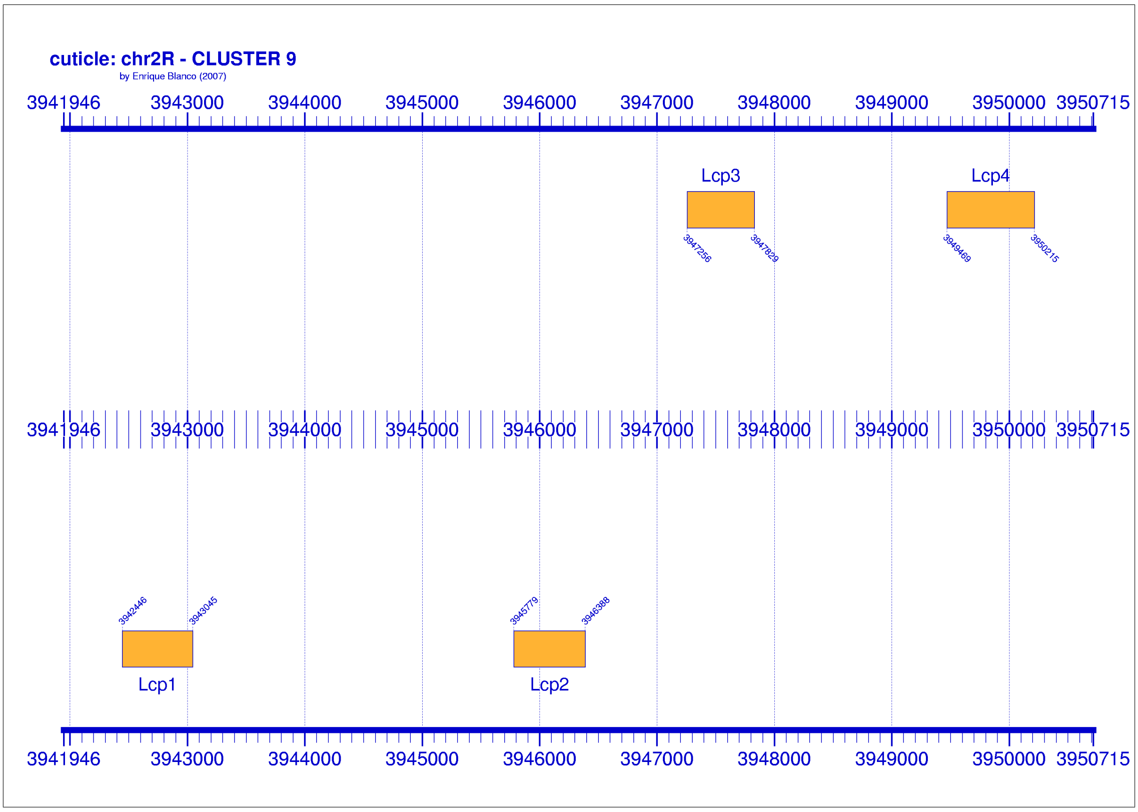

Enrique Blanco © 2007 — July 4, 2007

cuticle – chr2R: 6768035 - 6794348

Genomic components: 7 coregulated genes, 10 genes

CHR	Strand	Start	End	RefSeq	Name	Exons	Description
CHR2R	-	6768035	6768798	NM_136810	CG9079	3	CG9079-PA
CHR2R	+	6771075	6771890	NM_136811	CG13224	1	CG13224-PA
CHR2R	+	6772495	6774581	NM_136812	CG13223	7	CG13223-PA
CHR2R	-	6775223	6775746	NM_136813	CG9077	2	CG9077-PA
CHR2R	-	6777220	6777697	NM_136814	CG9076	2	CG9076-PA
CHR2R	+	6781031	6782767	NM_136815	CG13222	2	CG13222-PA
CHR2R	-	6782998	6786650	NM_136816	CG13214	17	CG13214-PA, isoform A
CHR2R	-	6788256	6788945	NM_165813	CG30027	1	CG30027-PA
CHR2R	-	6789942	6791198	NM_057620	TpnC47D	4	Troponin C at 47D CG9073-PA
CHR2R	-	6793857	6794348	NM_136817	CG9070	2	CG9070-PA

Cluster size: 26314 nucleotides

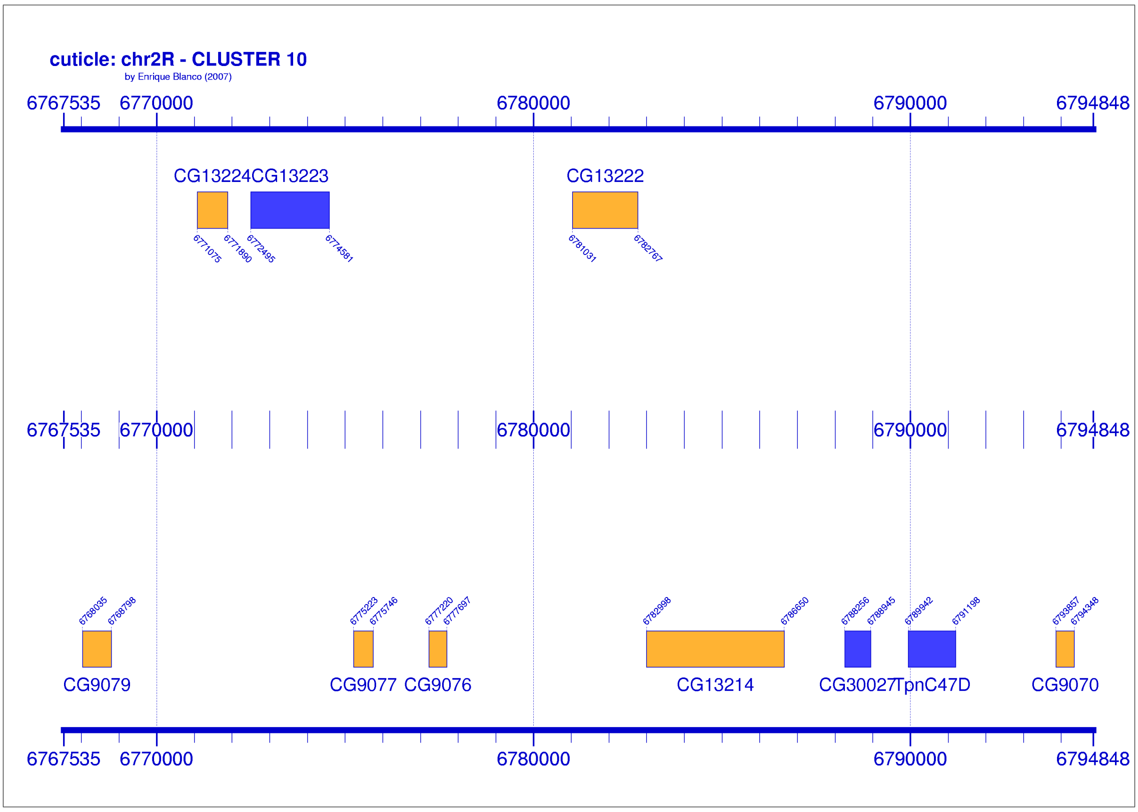

Enrique Blanco © 2007 — July 4, 2007

cuticle – chr2R: 7902885 - 7930760

Genomic components: 6 coregulated genes, 11 genes

Chr	Strand	Start	End	RefSeq	Name	Exons	Description
CHR2R	+	7902885	7905964	NM_136928	CG8502	7	CG8502-PA, isoform A
CHR2R	-	7906941	7907561	NM_136929	CG8836	3	CG8836-PA
CHR2R	+	7909389	7910747	NM_078987	Or49a	4	Odorant receptor 49a CG13158-PA
CHR2R	+	7910848	7915011	NM_165895	CG30048	3	CG30048-PA, isoform A
CHR2R	+	7918121	7921019	NM_136930	CG8505	3	CG8505-PA
CHR2R	+	7921911	7922348	NM_136931	CG8510	2	CG8510-PA
CHR2R	+	7923742	7924438	NM_136932	CG8511	2	CG8511-PA
CHR2R	+	7924802	7925559	NM_165897	CG30050	3	CG30050-PA
CHR2R	+	7925985	7926601	NM_001032234	CG33626	3	CG33626-PA
CHR2R	+	7926917	7927583	NM_001032235	CG33627	3	CG33627-PA
CHR2R	+	7929968	7930760	NM_136933	CG8515	2	CG8515-PA

Cluster size: 27876 nucleotides

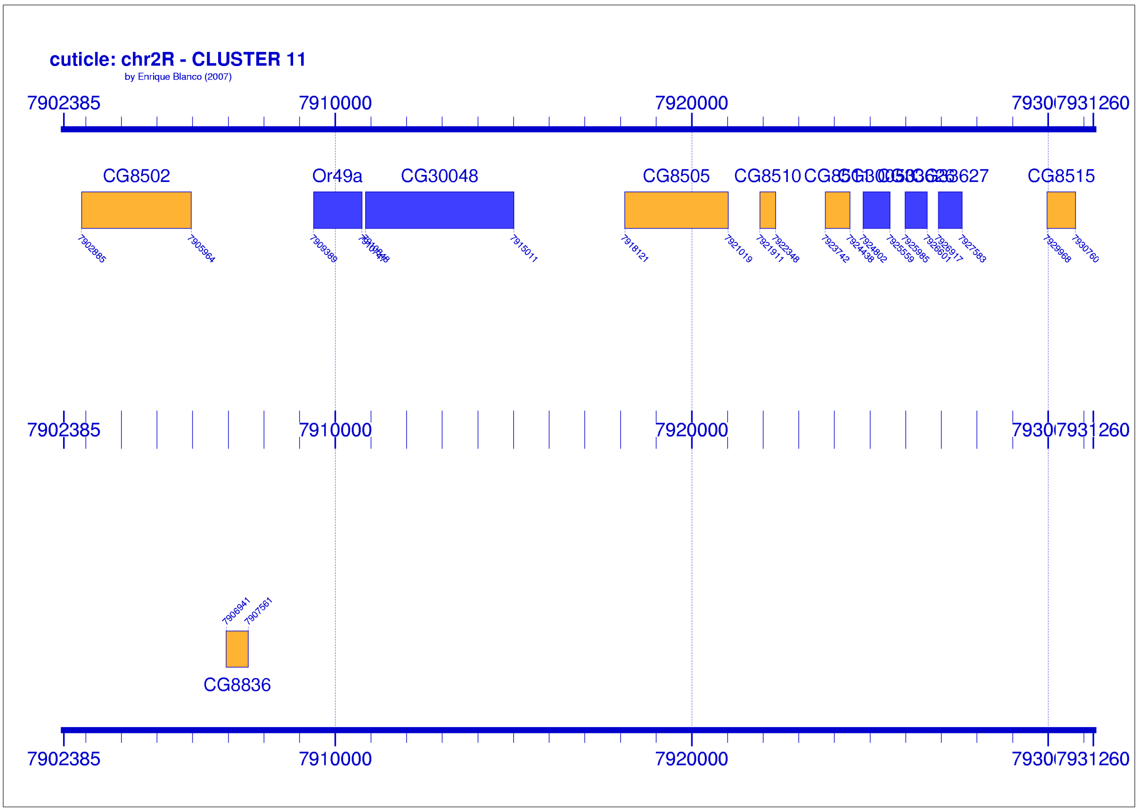

Enrique Blanco © 2007 — July 4, 2007

cuticle – chr3R: 2510171 - 2530625

Genomic components: 8 coregulated genes, 8 genes

CHR	Strand	Start	End	RefSeq	Name	Exons	Description
CHR3R	+	2510171	2510855	NM_079523	Edg84A	2	Ecdysone-dependent gene 84A CG2345-PA
CHR3R	+	2512449	2513761	NM_141420	Ccp84Ag	2	Ccp84Ag CG2342-PA
CHR3R	-	2515394	2515908	NM_141421	Ccp84Af	2	Ccp84Af CG1331-PA
CHR3R	-	2516611	2517480	NM_141422	Ccp84Ae	2	Ccp84Ae CG1330-PA
CHR3R	+	2518519	2519178	NM_141423	Ccp84Ad	2	Ccp84Ad CG2341-PA
CHR3R	-	2521188	2521993	NM_141424	Ccp84Ac	2	Ccp84Ac CG1327-PA
CHR3R	-	2527689	2528599	NM_141425	Ccp84Ab	2	Ccp84Ab CG1252-PA
CHR3R	+	2529803	2530625	NM_141426	Ccp84Aa	2	Ccp84Aa CG2360-PA

Cluster size: 20455 nucleotides

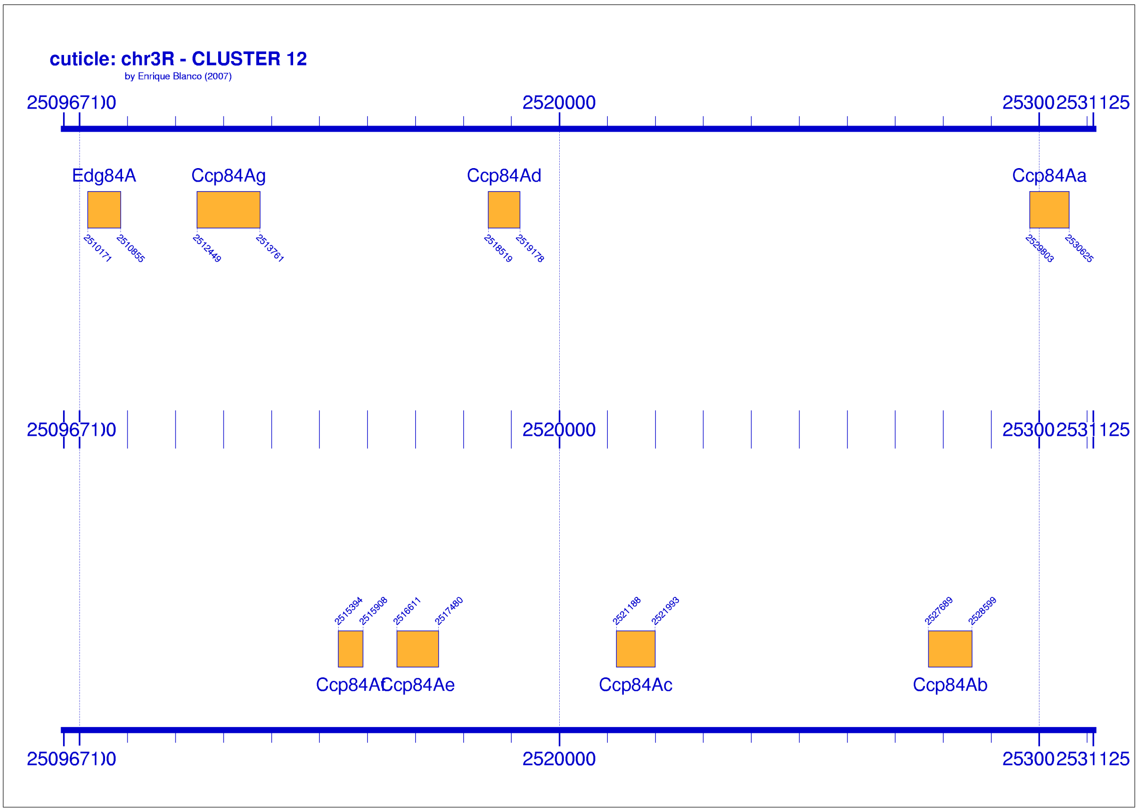

Enrique Blanco © 2007 — July 4, 2007

chitinbinding – chr3L: 2249925 - 2253369

Genomic components: 3 coregulated genes, 3 genes

CHR	Strand	Start	End	RefSeq	Name	Exons	Description
CHR3L	+	2249925	2251174	NM_139451	CG13806	2	CG13806-PA
CHR3L	+	2251401	2252060	NM_167943	CG32304	1	CG32304-PA
CHR3L	+	2252358	2253369	NM_167944	CG32302	1	CG32302-PA

Cluster size: 3445 nucleotides

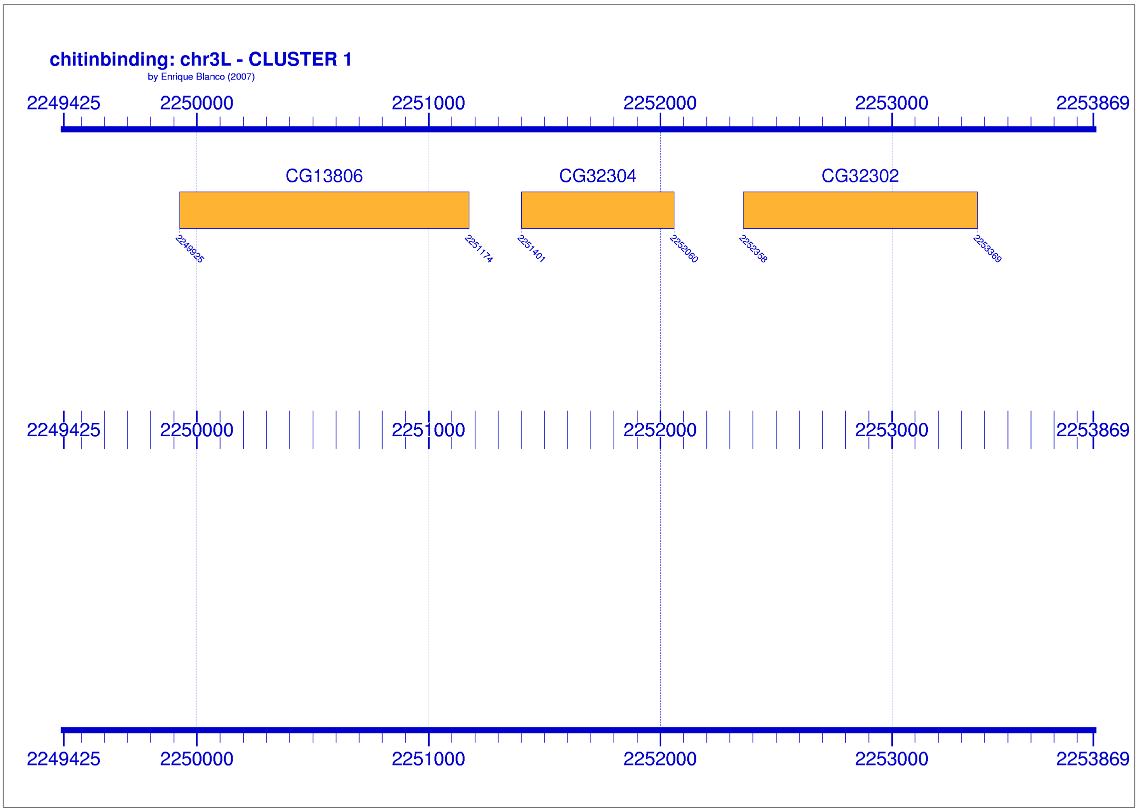

Enrique Blanco © 2007 — July 5, 2007

chitinbinding – chr3L: 11917595 - 11954877

Genomic components: 7 coregulated genes, 10 genes

CHR	Strand	Start	End	RefSeq	Name	Exons	Description
CHR3L	-	11917595	11918793	NM_140269	CG5883	2	CG5883-PA
CHR3L	+	11921632	11923195	NM_140270	CG7252	2	CG7252-PA
CHR3L	+	11923787	11926172	NM_140271	CG17826	2	CG17826-PA
CHR3L	-	11926347	11927240	NM_140272	CG9781	2	CG9781-PA
CHR3L	+	11928032	11930731	NM_140273	CG7248	2	CG7248-PA
CHR3L	-	11930761	11931438	NM_140274	CG11570	1	CG11570-PA
CHR3L	-	11932819	11940078	NM_079919	prc	9	pericardin CG5700-PB
CHR3L	-	11941528	11946926	NM_206342	CG33265	1	CG33265-PA
CHR3L	+	11948856	11949704	NM_140275	CG14125	2	CG14125-PA
CHR3L	+	11950060	11954877	NM_140276	CG6947	10	CG6947-PA

Cluster size: 37283 nucleotides

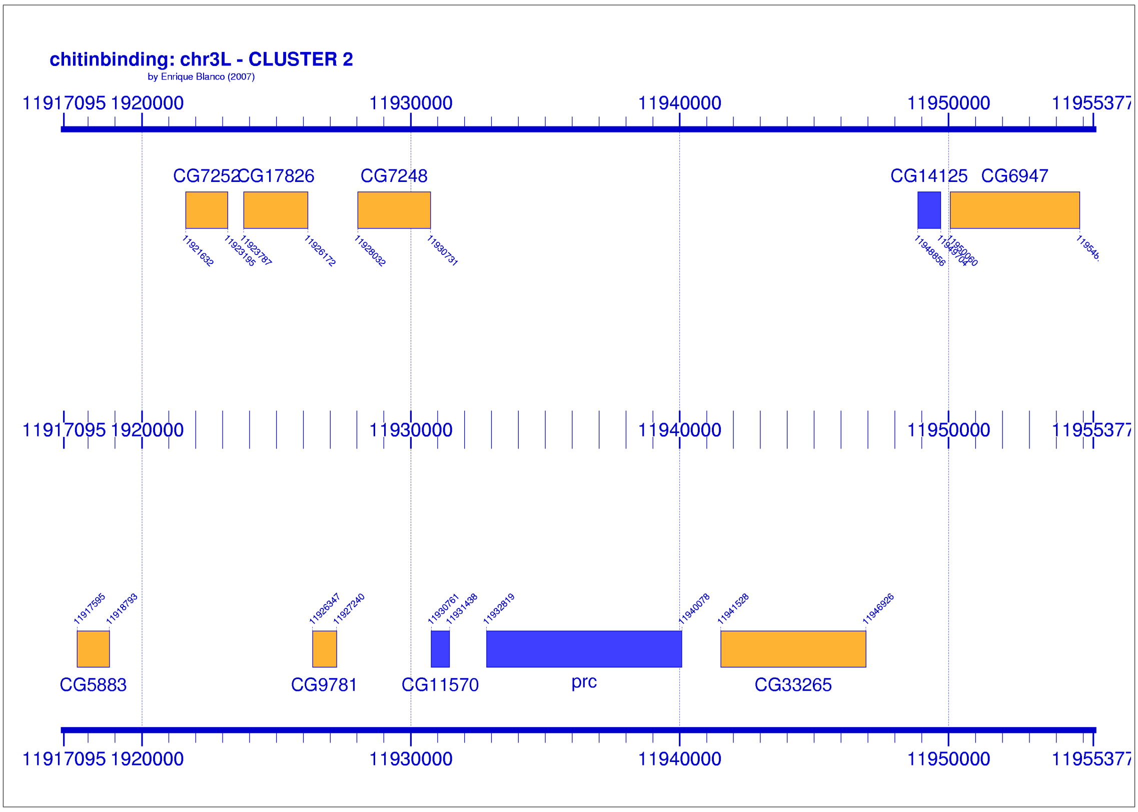

Enrique Blanco © 2007 — July 5, 2007

chitinbinding – chr3L: 11941528 - 11957487

Genomic components: 3 coregulated genes, 4 genes

CHR	Strand	Start	End	RefSeq	Name	Exons	Description
CHR3L	-	11941528	11946926	NM_206342	CG33265	1	CG33265-PA
CHR3L	+	11948856	11949704	NM_140275	CG14125	2	CG14125-PA
CHR3L	+	11950060	11954877	NM_140276	CG6947	10	CG6947-PA
CHR3L	+	11955074	11957487	NM_140277	CG17824	1	CG17824-PA

Cluster size: 15960 nucleotides

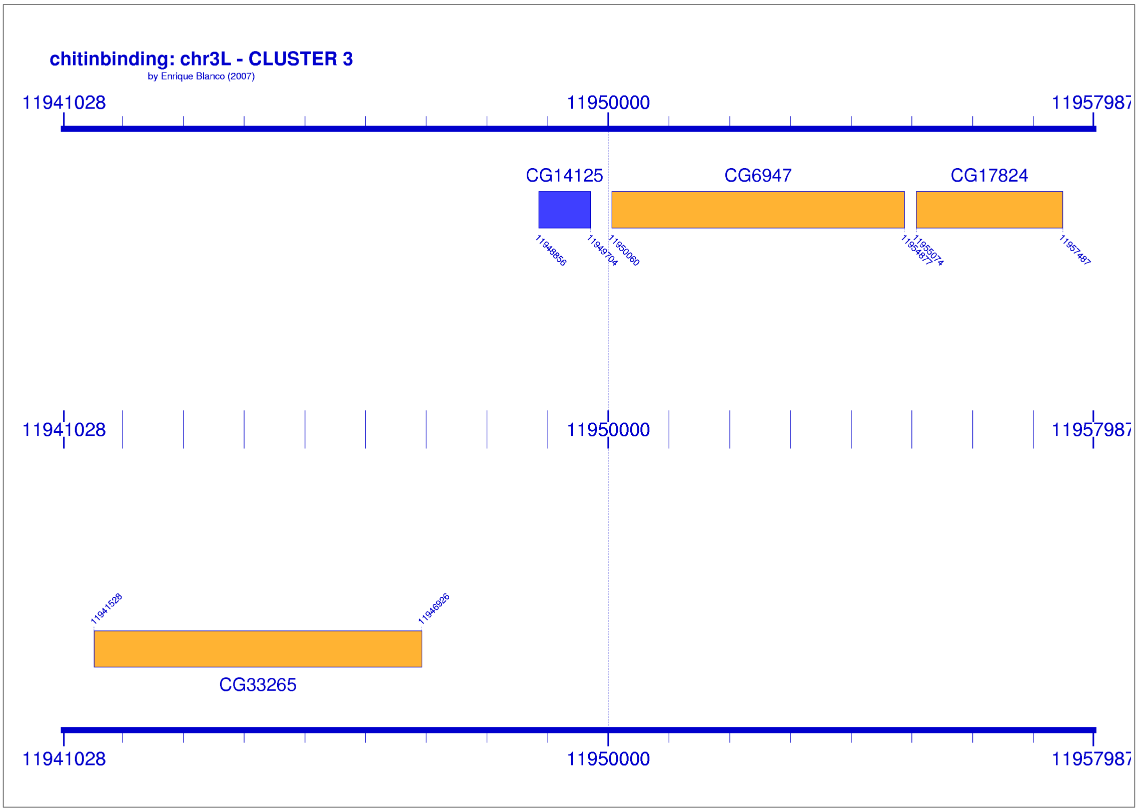

Enrique Blanco © 2007 — July 5, 2007

chitinbinding – chr3L: 13397431 - 13414382

Genomic components: 4 coregulated genes, 7 genes

CHR	Strand	Start	End	RefSeq	Name	Exons	Description
CHR3L	-	13397431	13398115	NM_206350	CG33263	1	CG33263-PA
CHR3L	-	13398517	13399615	NM_140386	CG14106	1	CG14106-PA
CHR3L	-	13399908	13400583	NM_140387	CG14105	3	CG14105-PA
CHR3L	-	13403377	13409176	NM_140388	CG10713	3	CG10713-PA
CHR3L	-	13409769	13411080	NM_140389	CG10154	4	CG10154-PA
CHR3L	+	13411905	13412956	NM_140390	CG10725	4	CG10725-PB
CHR3L	-	13413305	13414382	NM_140391	CG10140	4	CG10140-PA

Cluster size: 16952 nucleotides

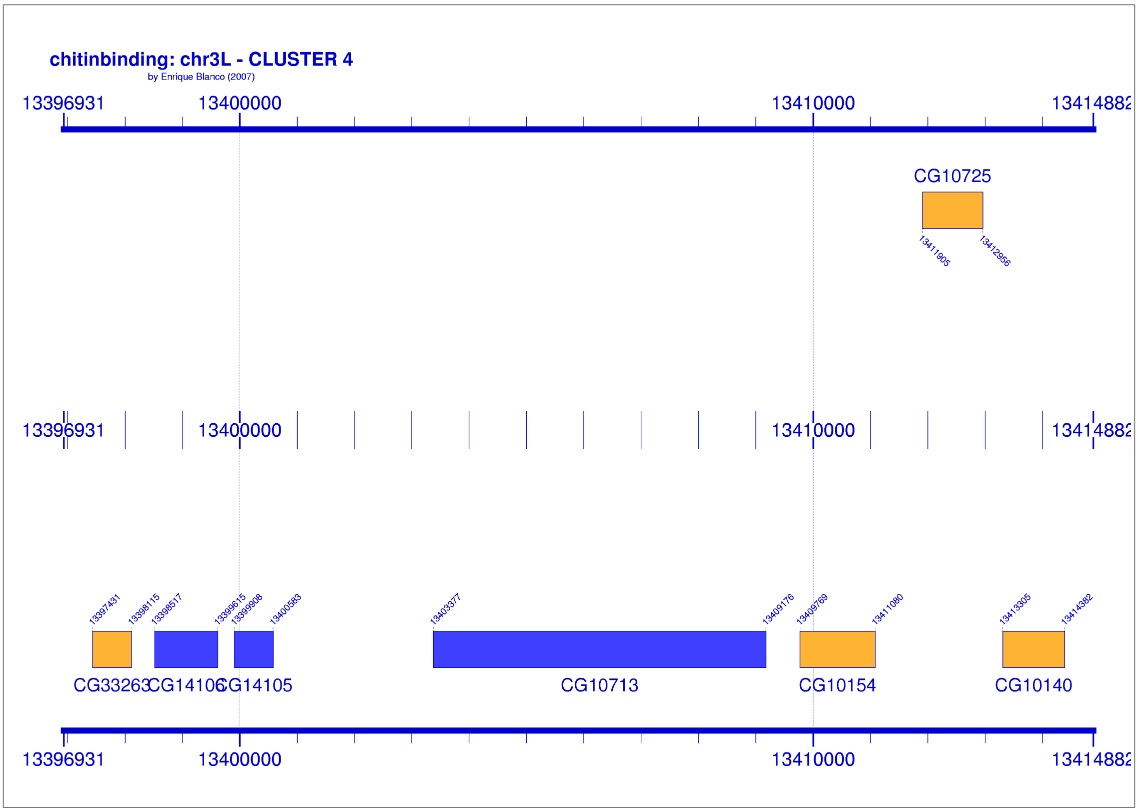

Enrique Blanco © 2007 — July 5, 2007

chitinbinding – chr3L: 15489590 - 15494489

Genomic components: 4 coregulated genes, 4 genes

CHR	Strand	Start	End	RefSeq	Name	Exons	Description
CHR3L	-	15489590	15490430	NM_001038928	CG33986	1	CG33986-PA
CHR3L	-	15491172	15492066	NM_001038929	CG33985	2	CG33985-PA
CHR3L	-	15492211	15493269	NM_001038930	CG33984	2	CG33984-PA
CHR3L	-	15493617	15494489	NM_001038931	CG33983	2	CG33983-PA

Cluster size: 4900 nucleotides

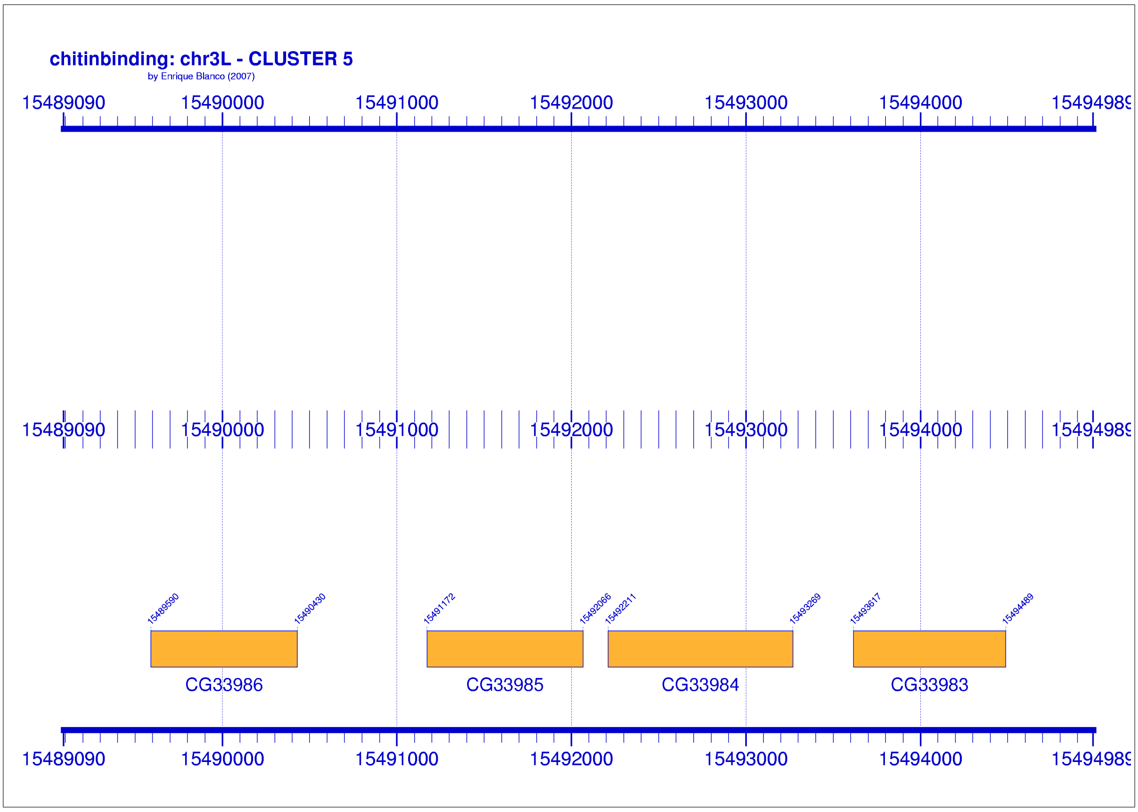

Enrique Blanco © 2007 — July 5, 2007

chitinbinding – chr3L: 20134283 - 20159132

Genomic components: 8 coregulated genes, 9 genes

CHR	Strand	Start	End	RefSeq	Name	Exons	Description
CHR3L	-	20134283	20135479	NM_140929	CG7306	2	CG7306-PA
CHR3L	-	20135905	20137527	NM_140930	CG7298	3	CG7298-PA
CHR3L	-	20138463	20139884	NM_140931	CG7290	2	CG7290-PA
CHR3L	+	20141740	20142798	NM_140932	CG6996	1	CG6996-PA
CHR3L	-	20141782	20143320	NM_168832	CG32224	2	CG32224-PA
CHR3L	-	20144276	20145959	NM_140933	CG7017	2	CG7017-PA
CHR3L	-	20152342	20154238	NM_140934	CG6933	2	CG6933-PA, isoform A
CHR3L	+	20155656	20156807	NM_140935	CG17145	2	CG17145-PA
CHR3L	+	20157729	20159132	NM_140936	CG17147	2	CG17147-PA

Cluster size: 24850 nucleotides

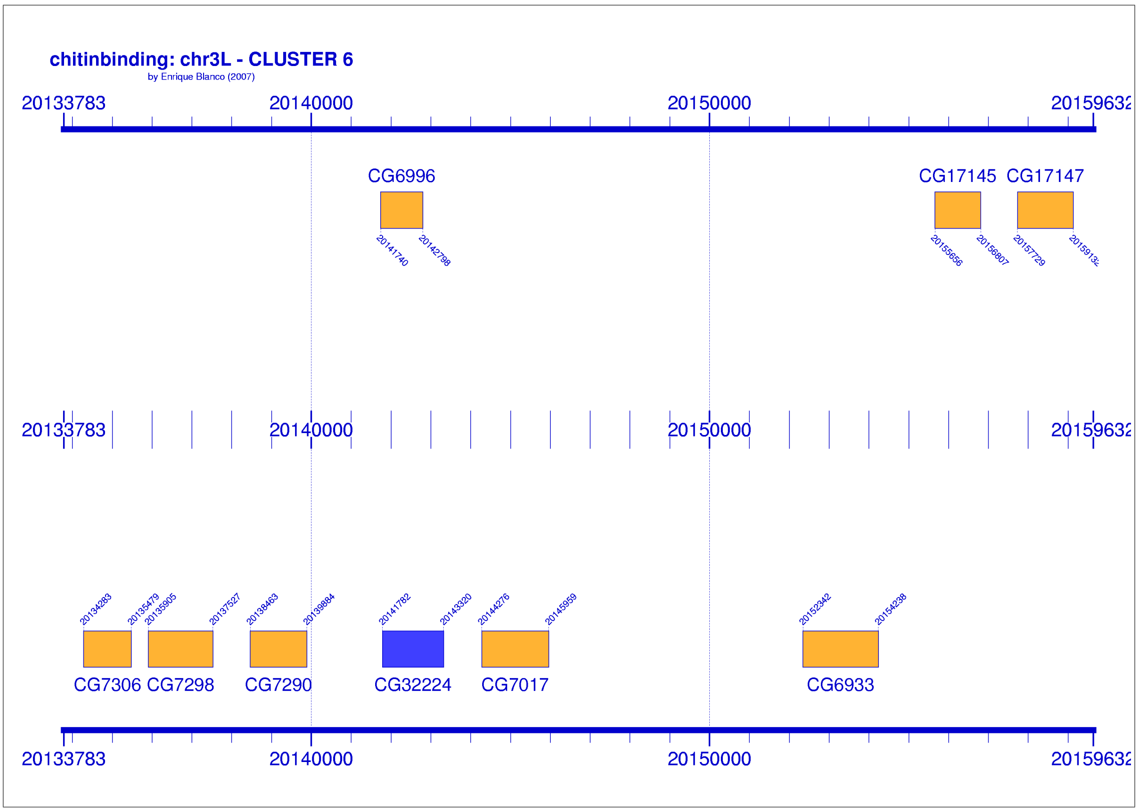

Enrique Blanco © 2007 — July 5, 2007

chitinbinding – chr2R: 16569227 - 16574669

Genomic components: 3 coregulated genes, 3 genes

CHR	Strand	Start	End	RefSeq	Name	Exons	Description
CHR2R	+	16569227	16570794	NM_137698	CG9357	3	CG9357-PA
CHR2R	-	16570759	16572545	NM_166420	CG30293	4	CG30293-PA
CHR2R	-	16572963	16574669	NM_080223	Cht4	5	Chitinase 4 CG3986-PA

Cluster size: 5443 nucleotides

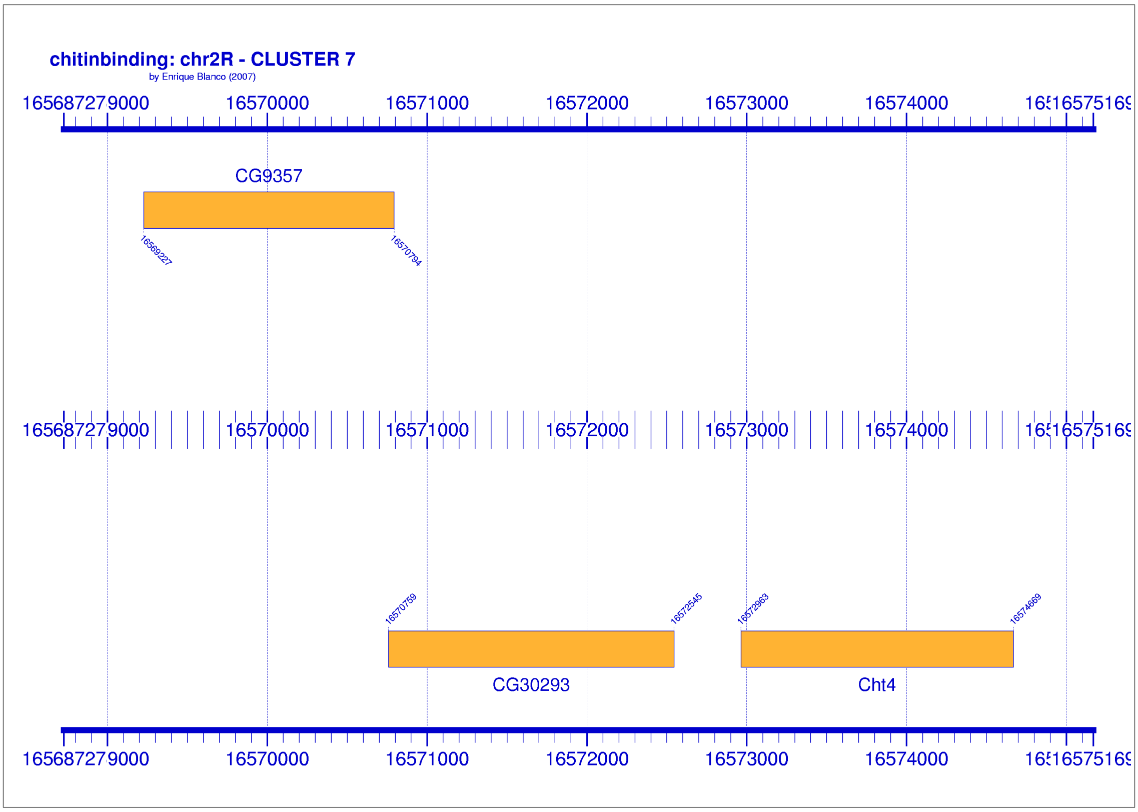

Enrique Blanco © 2007 — July 5, 2007
