# Supplementary material for: Conserved chromosomal clustering of genes governed by chromatin regulators in Drosophila
Source: Genome Biol. 2008 Sep 10;9(9):R134. doi: 10.1186/gb-2008-9-9-r134 (PMC2592712; doi:10.1186/gb-2008-9-9-r134)
Supplement: Additional data file 14 — Intersection between the trx clusters, the ChIP-on-chip information and the PRE/TRE predictions. [file gb-2008-9-9-r134-S14.pdf]

CLUSTER	Schwartz *et al.* (2006)	Tolhuis *et al.* (2006)	Ringrose *et al.* (2004)
1			
2		X	X
3		X	
4			
5			X
6	X		X
7	X		X
8			
9			X
10			
11			
12	X		
13			X
14			
15			
16			
17			
18			
19	X		
20	X		
21	X		X
22			
23			X
24			X
25	X		X
