# Supplementary material for: Conserved chromosomal clustering of genes governed by chromatin regulators in Drosophila
Source: Genome Biol. 2008 Sep 10;9(9):R134. doi: 10.1186/gb-2008-9-9-r134 (PMC2592712; doi:10.1186/gb-2008-9-9-r134)
Supplement: Additional data file 15 — Chromatin immunoprecipitation using anti-TRX and H3K4me3 specific antibodies to test the binding of TRX to the predicted PRE/TREs in clusters 9 and 25. [file gb-2008-9-9-r134-S15.pdf]

Trx binding sites in co-regulated clusters

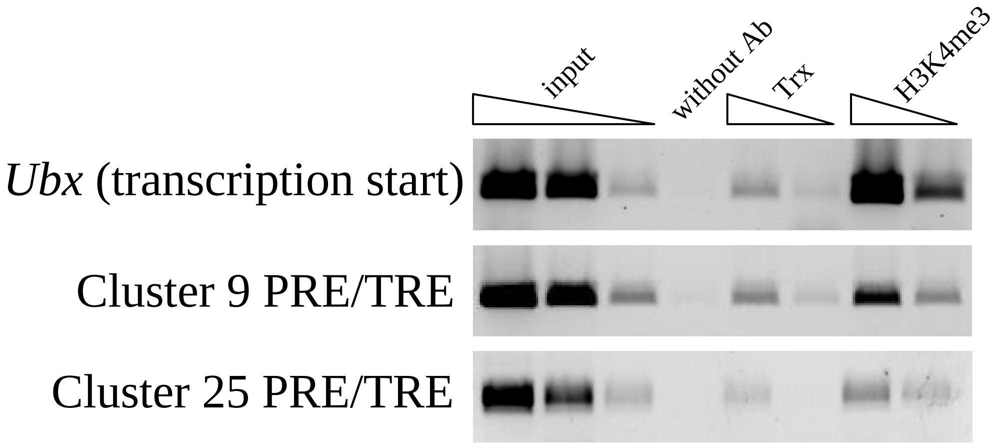

Chromatin immunoprecipitation of third instar larvae using anti-Trx and H3K4me3 specific antibodies. To test the binding of Trx to the predicted PRE/TREs we amplified regions of about 350 bp closely located to clusters 9 and 25. *Ubx* was used as a positive control.
