# Supplementary material for: Conserved chromosomal clustering of genes governed by chromatin regulators in Drosophila
Source: Genome Biol. 2008 Sep 10;9(9):R134. doi: 10.1186/gb-2008-9-9-r134 (PMC2592712; doi:10.1186/gb-2008-9-9-r134)
Supplement: Additional data file 16 — Examples of clusters and transfrags that detect similar patterns of expression. [file gb-2008-9-9-r134-S16.pdf]

cluster7

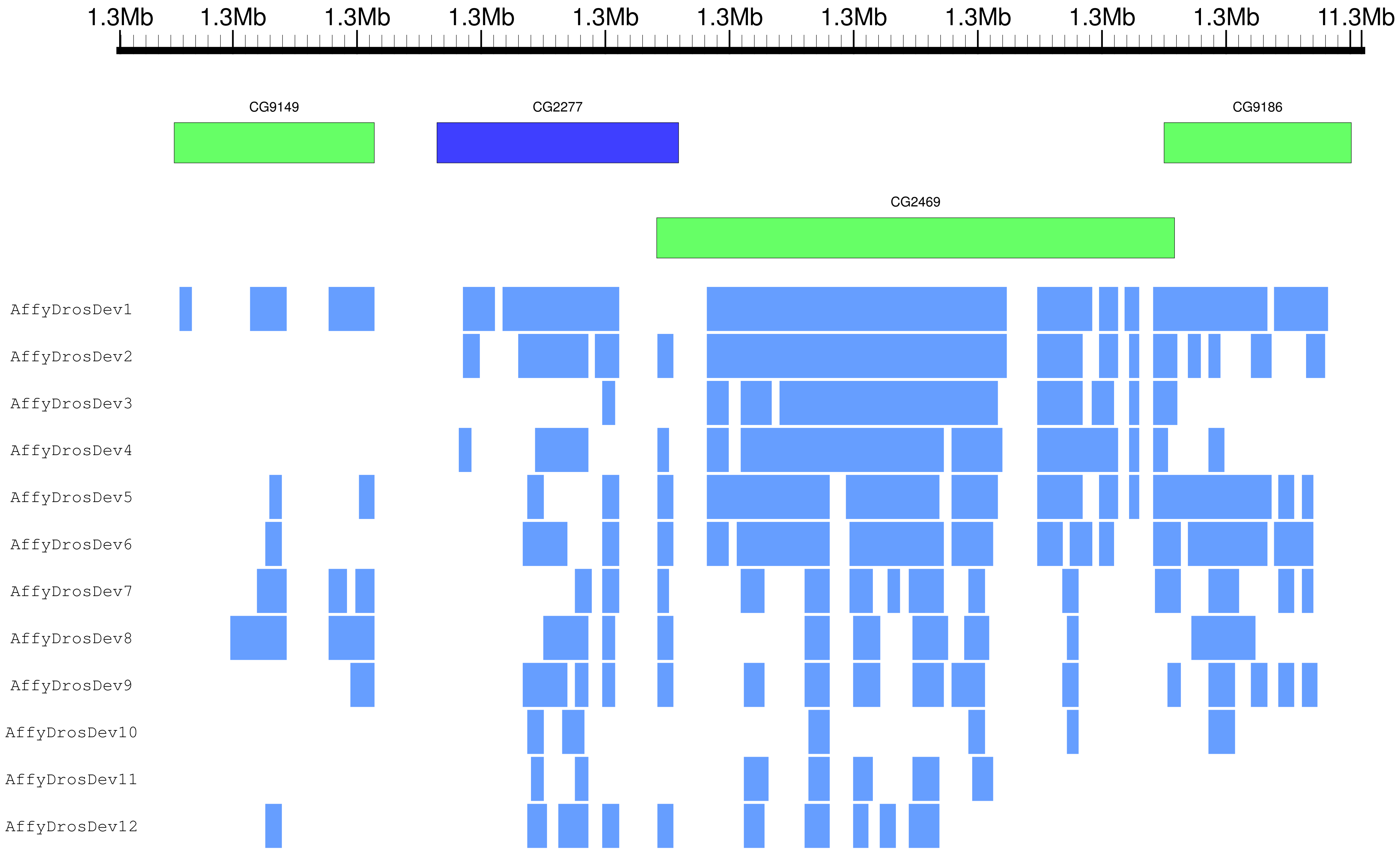

This plot has been obtained using gff2ps. The most recent version of gff2ps is freely available at "http://www1.imim.es/software/gfftools/GFF2PS.html". Copyright © 1999 by Josep F. Abril & Roderic Guigo

cluster9

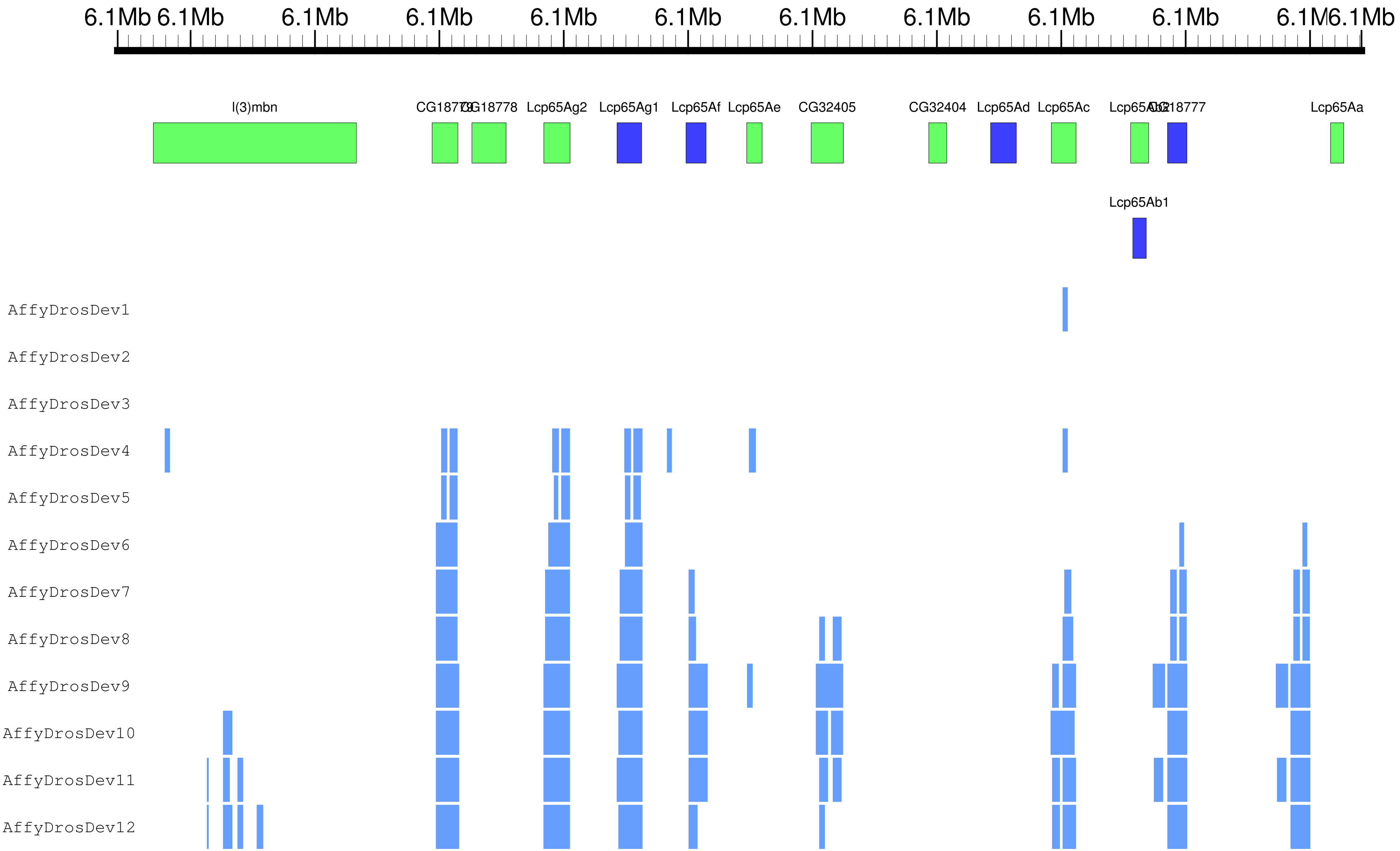

This plot has been obtained using gff2ps. The most recent version of gff2ps is freely available at "http://www1.imim.es/software/gfftools/GFF2PS.html". Copyright © 1999 by Josep F. Abril & Roderic Guigo

cluster24

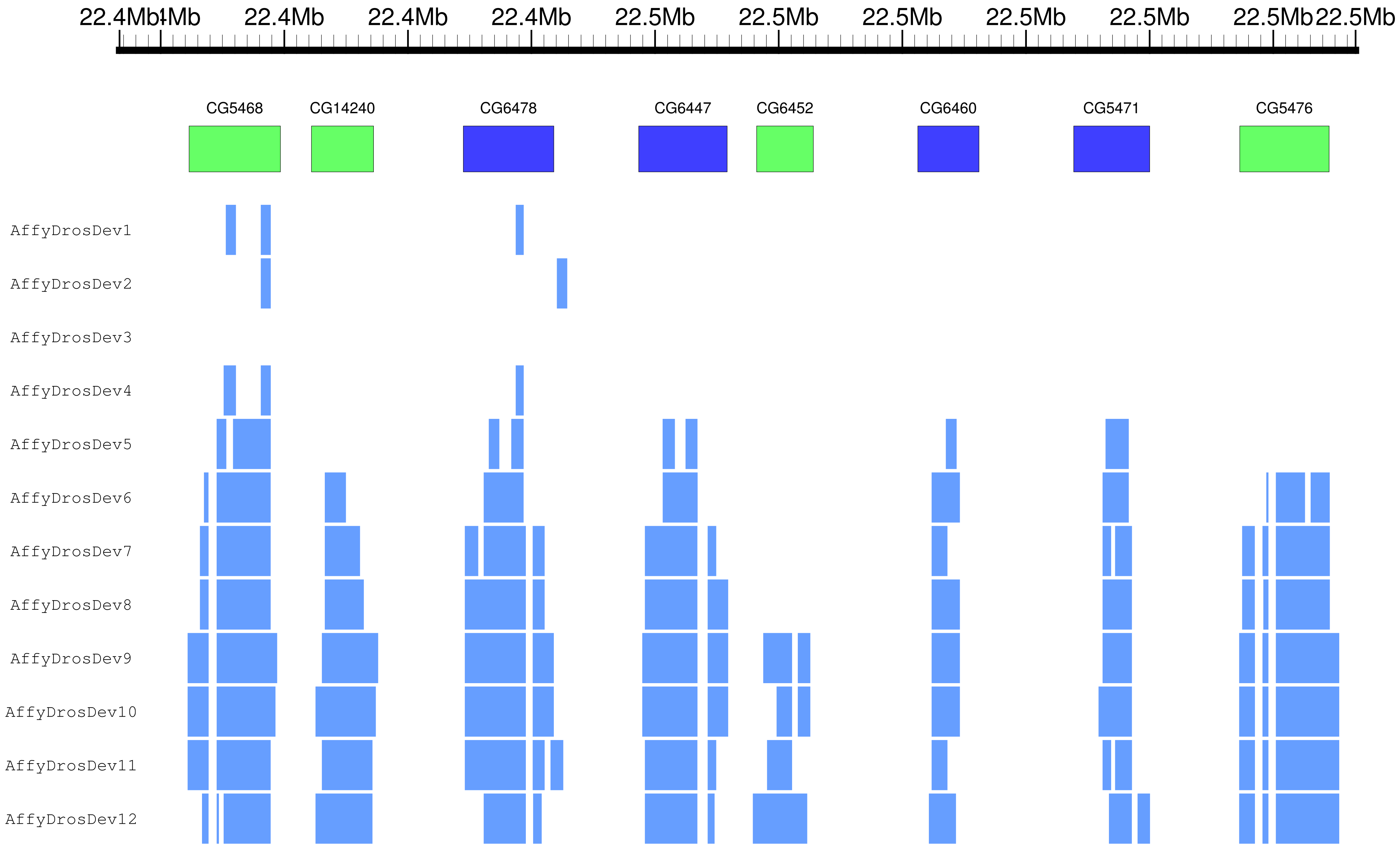

This plot has been obtained using gff2ps. The most recent version of gff2ps is freely available at "http://www1.imim.es/software/gfftools/GFF2PS.html". Copyright © 1999 by Josep F. Abril & Roderic Guigo
