# Supplementary material for: Conserved chromosomal clustering of genes governed by chromatin regulators in Drosophila
Source: Genome Biol. 2008 Sep 10;9(9):R134. doi: 10.1186/gb-2008-9-9-r134 (PMC2592712; doi:10.1186/gb-2008-9-9-r134)
Supplement: Additional data file 20 — Alternative overlap analysis among the clusters of genes regulated by different chromatin remodelers (only genes affected in the same way are considered). [file gb-2008-9-9-r134-S20.pdf]

	Common Clusters (1)		Common Clusters (2)	
Trithorax-ASH2	6	75%	4	50%
Trithorax-NURF	4	57%	2	29%
Trithorax-dMyc	6	100%	6	100%
Trithorax-ASH1	4	50%	2	25%
		71%		57%

Common clusters (1): cluster overlap considering genes affected in the same or the opposite way in the mutants analyzed. Common clusters (2): cluster overlap only considering genes affected in the same way in the mutants analyzed.
